# A Live-Cell NanoBRET Assay to Monitor RNA–Protein Interactions and Their Inhibition by Small Molecules

**DOI:** 10.1021/acscentsci.5c00705

**Published:** 2025-09-25

**Authors:** Jingsong Shan, Amirhossein Taghavi, Elizabeth A. Caine, Ryuichi Sekioka, Veronika Rajchin, James M. Burke, J. Monty Watkins, Jessica L. Childs-Disney, Matthew D. Disney

**Affiliations:** † Department of Chemistry, 4356 The Scripps Research Institute, 130 Scripps Way, Jupiter, Florida 33458, United States; ‡ Department of Chemistry, 145764 The Herbert Wertheim UF Scripps Institute for Biomedical Innovation and Technology, 130 Scripps Way, Jupiter, Florida 33458, United States; § 5240 Promega Corporation, 2800 Woods Hollow Road, Madison, Wisconsin 53711, United States; ∥ Department of Molecular Medicine, The Herbert Wertheim UF Scripps Institute for Biomedical Innovation and Technology, 130 Scripps Way, Jupiter, Florida 33458, United States

## Abstract

RNA–protein interactions are critical for cellular processes, including translation, pre-mRNA splicing, post-transcriptional modifications, and RNA stability. Their dysregulation is implicated in diseases such as myotonic dystrophy type 1 (DM1) and amyotrophic lateral sclerosis (ALS). To investigate RNA–protein interactions, here is described a live-cell NanoBioluminescence Resonance Energy Transfer (NanoBRET) assay to study the interaction between expanded r­(CUG) repeats [r­(CUG)^exp^] and muscleblind-like 1 (MBNL1), central to DM1 pathogenesis. This r­(CUG)^exp^ sequesters MBNL1, a regulator of alternative pre-mRNA splicing, in nuclear foci causing splicing dysregulation. In the NanoBRET assay, r­(CUG)^exp^ acts as a scaffold to bring into proximity a BRET pair, MBNL1–NanoLuciferase (NanoLuc) and MBNL1–HaloTag, enabling a quantitative readout of RNA–protein interactions. Following assay optimization, an RNA-focused small molecule library was screened, identifying ten compounds with shared chemotypes that disrupt the r­(CUG)^exp^–MBNL1 complex. Nuclear magnetic resonance (NMR) studies revealed these inhibitors bind to the 1 × 1 UU internal loops formed when r­(CUG)^exp^ folds. Five of these molecules rescued two cellular hallmarks of DM1 in patient-derived myotubes, alternative pre-mRNA splicing defects and formation of nuclear r­(CUG)/MBNL1-positive foci. These results demonstrate that the NanoBRET assay is a powerful tool to study RNA–protein interactions in live cells and to identify small molecules that alleviate RNA-mediated cellular pathology.

## Graphical abstract

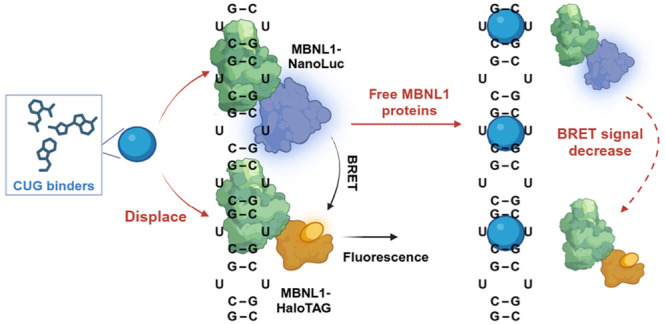

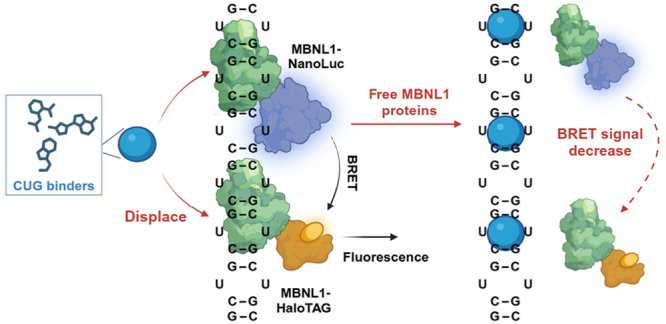

## Introduction

Noncoding (nc)­RNAs often elicit a biological response by interactions with RNA-binding proteins (RBPs). Many cellular processes depend upon the formation of RNA–protein interactions,^1^ including translation,^2,3^ alternative pre-mRNA splicing,^4–6^ post-transcriptional modifications (editing),^7–11^ cellular localization/trafficking,^1,12^ RNA quality control,^1,13–15^ and regulation of RNA lifetime.^1,15^ Both aberrant disruption and formation of RNA–protein interactions can cause diseases,^16^ including cancer, β-thalassemia,^17^ frontotemporal dementia,^18,19^ tauopathies,^20^ Prader-Willi Syndrome,^21^ and others. Indeed, various methods have been developed to identify and study RNA–protein interactions,^22^ including cross-linking methods such as cross-linking immunoprecipitation (CLIP)^23^ and variations thereof,^22,24,25^ chromatin isolation by RNA purification (ChIRP),^26^ and capture hybridization analysis of RNA targets (CHART).^27^ These methods, however, typically involve laborious workflows and require steps performed outside of a living cell. As such, these methods may not accurately reflect the dynamic and transient nature of RNA–protein interactions that occur within a cellular environment.

Microsatellite disorders are a class of >40 neuromuscular diseases caused by aberrant RNA–protein interactions mediated by RNA repeat expansions.^28,29^ The pathogenic mechanisms of repeat expansion disorders are multifaceted and are influenced by the location of the repeat within the gene, e.g. the 5′ or 3′ untranslated regions (UTRs), open reading frames (ORFs), or introns. In general, repeat expansions harbored in ORFs are translated and lead to the generation of toxic proteins that are often aggregation-prone.^30,31^ In contrast, expansions in noncoding regions such as UTRs and introns often exert toxicity at the RNA level. In these cases, the structured repeat has a toxic gain-of-function where they bind and sequester RBPs or can be translated by repeat-associated non-ATG (RAN) translation.^28,32^

A series of studies traced the cause of myotonic dystrophy type 1 (DM1) to the presence of an expanded trinucleotide repeat expansion, r­(CUG)^exp^, in the 3′ UTR of *dystrophia myotonica protein kinase* (*DMPK*) mRNA.^33–41^ When repeat length exceeds >50 repeats, the RNA folds upon itself forming a periodic array of 1 × 1 nucleotide UU internal loops that are high affinity binding sites for muscleblind-like 1 (MBNL1) and other RBPs (Figure 1). Sequestration of these protein by the RNA in nuclear foci^34,35,42,43^ leads to their inactivation, and, in the case of MBNL1, aberrant alternative pre-mRNA splicing of its substrates.^40,44–46^ As multiple copies of MBNL1 bind to r­(CUG)^exp^ repeats, each with nM affinity,^47^ this system was hypothesized to be suitable for development of a NanoBioluminescence Resonance Energy Transfer (NanoBRET) assay^48^ that reports on the toxic RNA–protein interaction that drives DM1 pathology in live cells, allowing interrogation and modulation of these complexes in an intact biological system. Herein, the development of such an assay is reported that was then used to identify small molecules that alleviate DM1-associated cellular phenotypes in patient-derived myotubes. In brief, the assay utilizes a cell line that stably expresses the repeat expansion, which is transfected with MBNL1–NanoLuciferase (NanoLuc) and MBNL1–HaloTag that produce a BRET signal upon binding to r­(CUG)^exp^.

**Figure 1:**
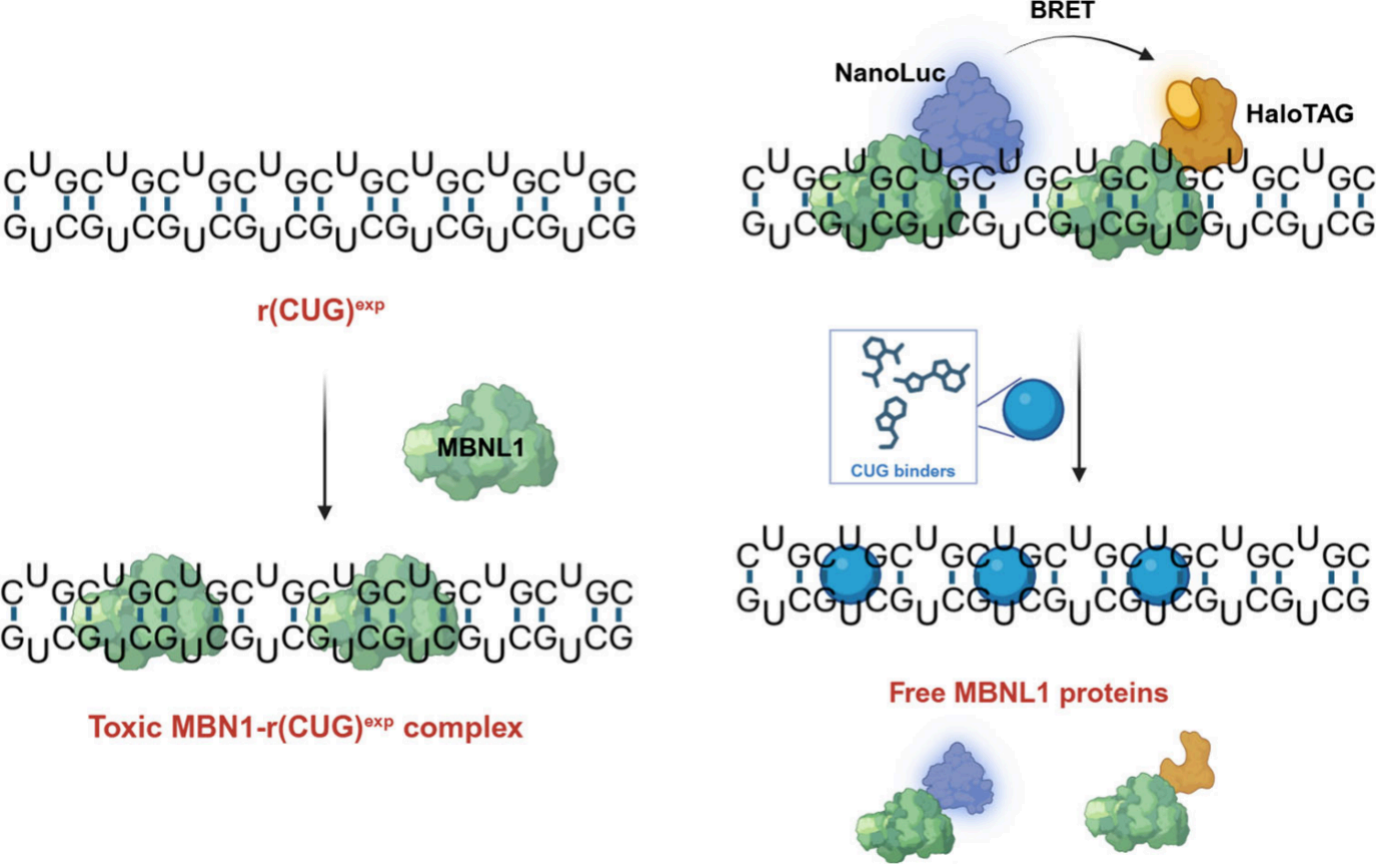
**Schematic of expanded r­(CUG) repeats [r­(CUG)** ^**exp**^ **] forming toxic complexes with MBNL1 and their disruption by small molecules**. (Left) Expanded r­(CUG) repeats [r­(CUG)^exp^] form stable secondary structures that are high affinity binding sites for MBNL1. Sequestration of MBNL1 causes a loss of function that leads to dysregulation of alternative pre-mRNA splicing and hence DM1-associated cellular phenotypes. (Right) The binding of MBNL1 proteins tagged with NanoLuc (blue) and HaloTag (orange) to r­(CUG)^exp^ generates a bioluminescence resonance energy transfer (BRET) signal. Upon treatment with small molecules that bind r­(CUG)^exp^ (turquoise), the toxic r­(CUG)^exp^–MBNL1 complex is disrupted, reducing the BRET signal.

## Results

### Design of Constructs and Cell Line Selection to Implement a NanoBRET Assay to Study r­(CUG)^exp^–MBNL1 Complexes

As mentioned above, when the expanded trinucleotide repeat expansion r­(CUG)^exp^ folds, it forms 1 × 1 nucleotide UU internal loops (5′CUG/3′GUC) that bind multiple copies of MBNL1 (Figure 1). Previous studies have shown that approximately two 5′CUG/3′GUC motifs bind to one copy of MBNL1.^47^ As the average repeat length in DM1-affected patients is ∼500,^49^ MBNL1 accumulates in nuclear foci containing long repeats in a pathogenic setting.^39,42,47,50^ It was therefore hypothesized that two MBNL1 molecules brought into proximity by r­(CUG)^exp^ could generate a BRET signal.

To determine the optimal fusion proteins for live cell BRET, four different constructs were designed: MBNL1 fused to NanoLuciferase on either N- (NanoLuc–MBNL1) or the C-terminus (MBNL1–NanoLuc) and MBNL1 fused to a HaloTag also on either the N- (HaloTag–MBNL1) or the C-terminus (MBNL1–HaloTag) (see Table S1 for sequences). A HaloTag is a modified haloalkane dehalogenase that forms a covalent bond with exogenously added HaloTag ligands, here attached to a fluorescent dye through a chloroalkane linker.^51^ A BRET signal would therefore be produced in the presence of the HaloTag ligand when NanoLuc and HaloTag MBNL1 fusions are within a BRET radius, ∼50 Å,^52^ afforded by binding to r­(CUG)^exp^ (Figure 1). To simplify the cellular reporter assay, HeLa cells that stably express a mutant allele harboring an interrupted r­(CUG)_480_ tract of 24 tandem modules of [(CUG)_20_(CUCGA)], and a wild type allele, r­(CUG)_0_, were employed, dubbed HeLa480.^53^ Expression of the interrupted repeat cause alternative pre-mRNA splicing defects and formation of nuclear foci,^53^ hallmarks of DM1 pathology. As a single MBNL1 protein binds about two 5′CUG/3′GUC motifs^47^ and assuming that the interrupted module folds into a CUCGA hairpin with ten 5′CUG/3′GUC motifs, approximately five MBNL1 proteins could bind to each interrupted repeat tract. Thus, despite the interrupted nature, MBNL1 proteins can bind within a NanoBRET radius (3.08–9.23 nm).^54^ Moreover, MBNL1 binding to adjacent RNA molecules in foci could also generate a BRET signal, where a reduction in BRET signal would suggest that the foci have been dissolved/reduced in size to some degree. This cell line was designed such that the two alleles can be distinguished by RT-qPCR by using TaqMan probes unique to each (Table S2).

A C-terminal deletion of MBNL1 was used in each fusion to prevent self-dimerization, which would confound analysis.^55^ Exons 5, 6, and 7 have been reported to contribute to the nuclear localization of MBNL1.^56,57^ Exons 6 and 7 are part of the C-terminal deletion employed in this study, and while its removal alters the distribution of MBNL1, immunocytochemistry (IHC) of MBNL1–NanoLuc and single molecule fluorescence *in situ* hybridization (smFISH) imaging of r­(CUG)^exp^ in the HeLa480 cell line confirmed both the nuclear localization of MBNL1–NanoLuc and its colocalization with RNA expansion repeats, although MBNL1 was also detected in the cytosol (Figure 2).

**Figure 2:**
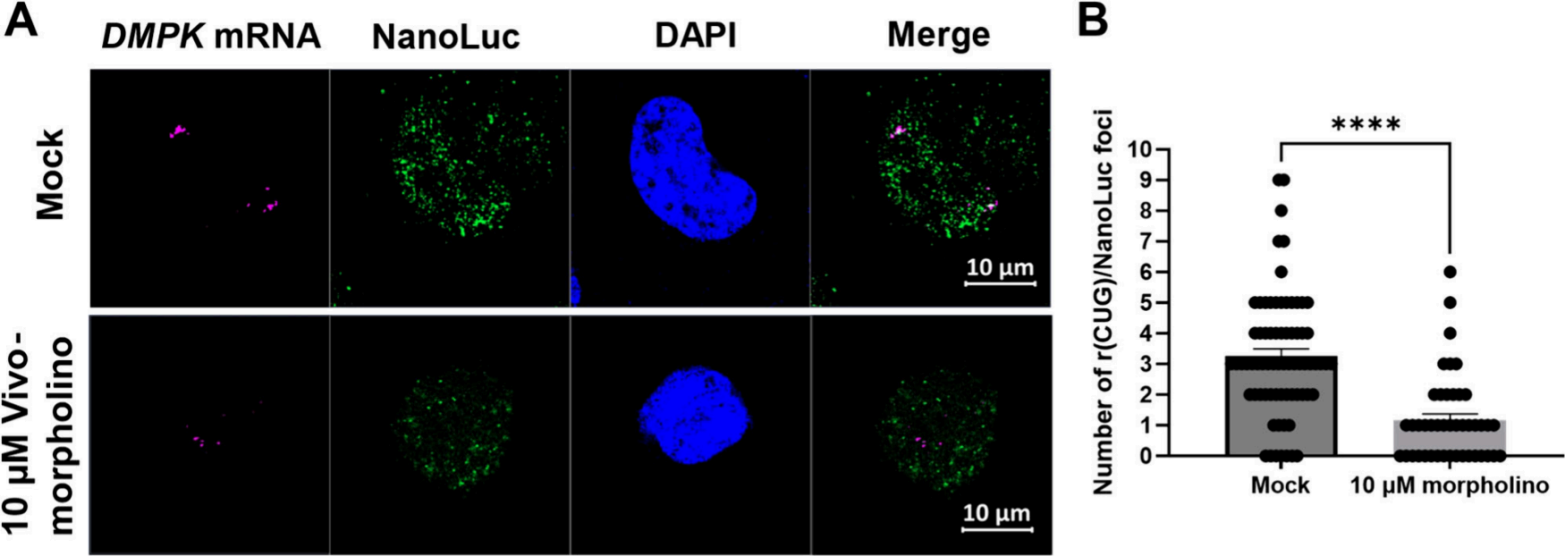
**Colocalization of MBNL1–NanoLuc with nuclear foci and quantification of foci number upon Vivo-Morpholino treatment**. (A) Immunofluorescence assay (IFA) for NanoLuc (green) and smRNA-FISH for *DMPK mRNA* (magenta). Representative images showing the colocalization of MBNL1–NanoLuc with r­(CUG)^exp^ in HeLa480 cells. MBNL1–NanoLuc was detected using an anti-NanoLuc antibody (green), and r­(CUG)^exp^ was imaged using smFISH probes targeting *DMPK* exons 11–15. DAPI staining (blue) was used to visualize nuclei. In mock-treated cells (top row), localization of MBNL1–NanoLuc to nuclear foci containing r­(CUG)^exp^ is observed, as indicated by white signals in the merged images. Treatment with 10 μM Vivo-Morpholino (bottom row) significantly reduces the number and intensity of nuclear foci, resulting in a more diffuse nuclear distribution of MBNL1–NanoLuc. Scale bar is 10 μm. (B) Quantification of the number of r­(CUG)^exp^-NanoLuc foci (white in merged images) per cell under mock and 10 μM CAG25 Vivo-Morpholino treatment conditions (with 40 nuclei quantified/replicate; *n* = 3 biological replicates). Data are reported as mean ± SEM, with statistical significance determined by a two-tailed unpaired Student’s *t* test. Significance thresholds; *, *p* < 0.05; **, *p* < 0.01; ***, *p* < 0.001; ****, *p* < 0.0001.

### Assay Optimization and Validation

To identify conditions that provide robust BRET signal, the ratio of the plasmids encoding the two C-terminal fusions (NanoLuc and HaloTag) were systematically altered, where the ratio of plasmid amount used in the transfection ranged from 1:10 to 1:200. Here, three factors were assessed: (i) donor luminescence signal; (ii) the observed BRET signal generated by the juxtaposition of NanoLuc- and HaloTag-fused MBNL1 proteins; and (iii) maximal BRET signal reduction achievable, as assessed by knock-down of r­(CUG)^exp^ abundance using a complementary Vivo-Morpholino (Gene Tools, LLC) antisense oligonucleotide (ASO; CAG25 Vivo-Morpholino).^41^ This oligonucleotide contains an octaguanidine dendrimer that facilitates cellular uptake, allowing it to be added directly to cell culture medium without the need for transfection. Because luminescence from extracellular NanoLuc can contribute to excess donor signal, thereby reducing BRET ratio, an extracellular NanoLuc inhibitor was employed. This membrane-impermeable molecule selectively quenches NanoLuc signal from the extracellular environment to ensure that only intracellular MBNL1–NanoLuc luminescence was detected.^58^ The heatmap in Figure 3B illustrates the window size for each ratio of MBNL1–NanoLuc: MBNL1–HaloTag in the presence and absence of 10 μM of CAG25 Vivo-Morpholino. The largest window size (reduction of ∼15 mBU in the correct BRET ratio; *p* = 0.0002) was observed at a plasmid ratio of 1:200 MBNL1–NanoLuc: MBNL1–HaloTag (12.5 ng of the former and 2500 ng of the latter). However, the donor (NanoLuc) luminescence signal at this ratio (∼1 × 10^4^) was below the optimal range for reliable detection (∼1 × 10^5^).^59^ Comparatively, plasmid ratios of 1:100 and 1:50 of MBNL1–NanoLuc: MBNL1–HaloTag (25 ng: 2500 ng and 50 ng: 2500 ng, respectively) produced similar window sizes (∼13.5; *p* = 0.0001), but they differed dramatically in the donor luminescence intensity. The 1:50 (50 ng: 2500 ng) ratio yielded a donor luminescence signal of ∼1 × 10^5^, which falls within the optimal range and ensures a donor luminescence signal of >1,000-fold above background and is essential for confidently measuring energy transfer to the acceptor HaloTag.^60^

**Figure 3:**
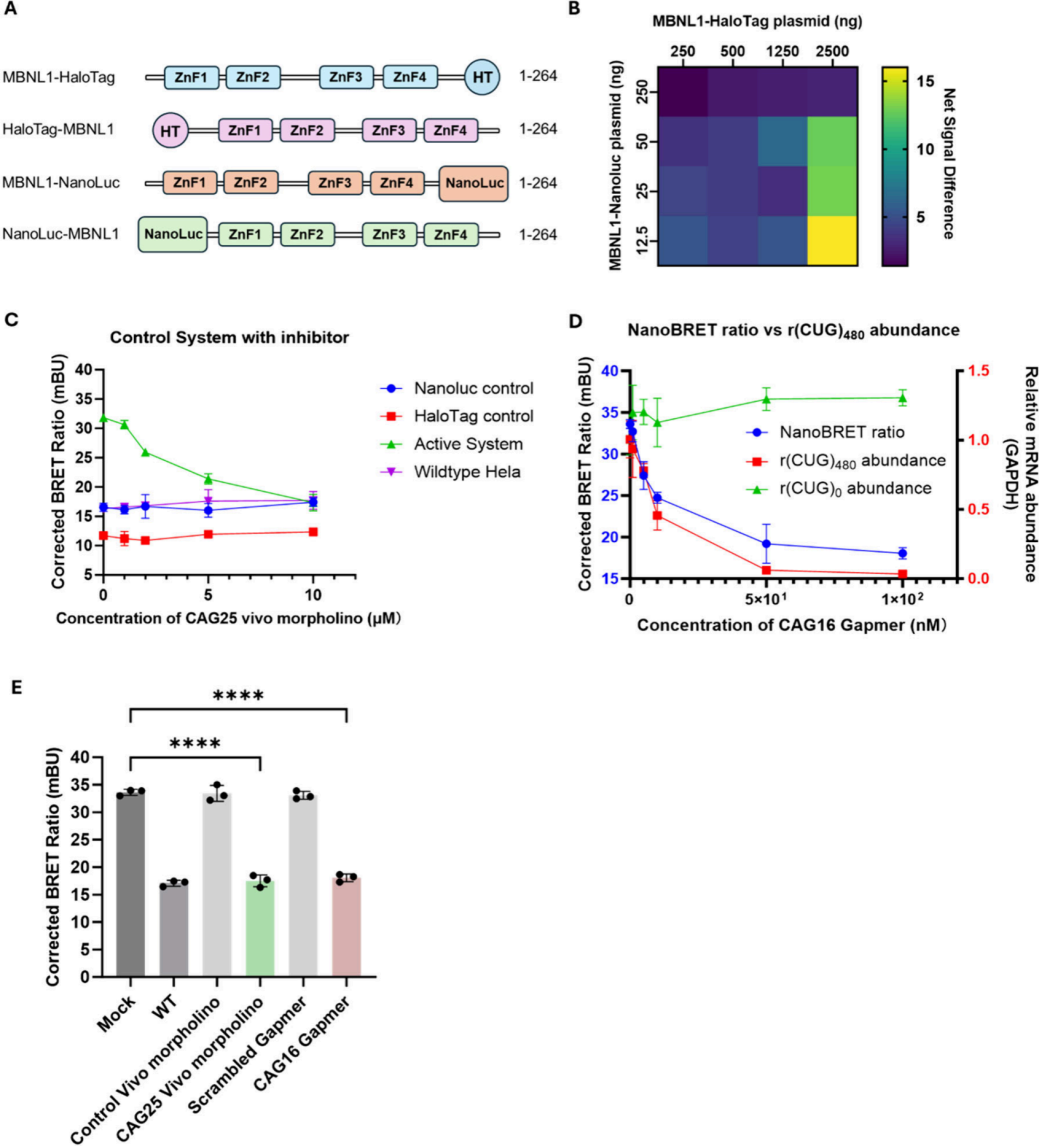
**Optimization and characterization of a NanoBRET assay to detect RNA–protein interactions, as applied to MBNL1 and r­(CUG)** ^**exp**^. (A) Schematic of fusion constructs used in the NanoBRET assay. MBNL1 was fused to either HaloTag (HT) or NanoLuciferase (NanoLuc) at the N- or C-terminus, affording four protein fusions: (i) MBNL1–HaloTag; (ii) HaloTag–MBNL1; (iii) MBNL1–NanoLuc, and (iv) NanoLuc–MBNL1. The four zinc finger domains (ZnF1–ZnF4), which bind RNA, are indicated to illustrate the structural layout of MBNL1 in each construct. (B) Heatmap showing the window size, defined as the reduction in the BRET signal following treatment with 10 μM of CAG25 Vivo-Morpholino, across different plasmid ratios of MBNL1–HaloTag:MBNL1–NanoLuc. A combination of 50 ng MBNL1–Nanoluc and 2500 ng MBNL1–HaloTag was selected for subsequent assays due to its window size (∼13.5 mBU) and optimal donor luminescence signal (∼100,000 RLU). Data are reported as mean ± SD (*n* = 3 biological replicates). (C) Effect of CAG25 Vivo-Morpholino on the NanoBRET signal in control systems: (i) HeLa480 cells transfected with MBNL1–HaloTag + NanoLuc (no MBNL1 fusion; blue); (ii) HeLa480 cells transfected with HaloTag (no MBNL1) + MBNL1–NanoLuc (red); and (iii) WT HeLa cells [no r­(CUG)^exp^] transfected with MBNL1–NanoLuc and MBNL1–HaloTag (purple). “Active System” indicates HeLa480 cells were transfected with MBNL1–NanoLuc and MBNL1–HaloTag (green). (D) Effect of CAG16 Gapmer (induces RNase H degradation of r­(CUG)^exp^) on NanoBRET signal (blue, left *y*-axis), on r­(CUG)_480_ (mutant allele) abundance normalized to *GAPDH* (red, right *y*-axis), and on r­(CUG)_0_ (wild type allele) abundance normalized to *GAPDH* (green, right *y*-axis) in HeLa480 cells. A strong correlation (Pearson r = 0.98) was observed, with RNA degradation and NanoBRET signal reduction reaching baseline levels at higher concentrations of the ASO (50–100 nM), reflecting that the r­(CUG)^exp^–MBNL1 protein interaction is no longer present. Data are reported as mean ± SD (*n* = 3 biological replicates). (E) Specificity of NanoBRET signal reduction with targeted antisense oligonucleotides. HeLa480 cells were treated with 10 μM CAG25 Vivo-Morpholino, 100 nM CAG16 Gapmer, and their respective scrambled controls at same concentration. Data are reported as mean ± SD (*n* = 3 biological replicates).

Following plasmid ratio optimization, the next step involved evaluating all possible combinations of N- and C-terminal fusions of MBNL1 with HaloTag and NanoLuc at a fixed 1:50 plasmid ratio (Figure 3A). While all orientations produced donor luminescence suitable for NanoBRET (Figure S1A), the greatest reduction in the NanoBRET signal upon treatment with 5 μM and 10 μM CAG25 Vivo-Morpholino was observed using the C-terminal fusions MBNL1–HaloTag and MBNL1–NanoLuc, compared to untreated samples (Figure S1B). The BRET ratio was reduced from 30.3 ± 0.4 to 19.3 ± 0.5 (*p* < 0.0001) at the 10 μM dose, demonstrating a reasonable signal-to-noise ratio with low standard deviation. Thus, all subsequent assay development was completed at a ratio of 1:50 MBNL1–HaloTag:MBNL1–NanoLuc, which did not affect cell viability (Figure S1C).

To verify that the NanoBRET signal generated was indeed reporting on the binding of MBNL1 to r­(CUG)^exp^, HeLa480 cells were cotransfected with MBNL1–HaloTag and NanoLuc (lacking MBNL1) or HaloTag (lacking MBNL1) and MBNL1–NanoLuc at ratios of 1:50. Although basal BRET ratios varied due to differences in plasmid expression and cell backgrounds, a reduction in the NanoBRET signal was only observed in HeLa480 cells treated with the CAG25 Vivo-Morpholino targeting the MBNL1–NanoLuc/MBNL1–HaloTag (active) system. Notably, the CAG25 Vivo-Morpholino had no effect on wild-type HeLa cells, which do not express r­(CUG)^exp^, under the same assay conditions (Figure 3C). All donor luminescence values remained within the optimal range for NanoBRET detection, confirming the robustness of the assay (Figure S2A). Additionally, background BRET ratios measured in control samples without addition of the HaloTag NanoBRET 618 ligand remained consistent across all constructs (Figure S2B). These findings confirm the assay’s specificity in detecting MBNL1 binding to r­(CUG)^exp^. To further validate the assay’s ability to detect disruption of the r­(CUG)^exp^–MBNL1 interaction via direct engagement with MBNL1, a previously reported small molecule that binds MBNL1 and disrupts its interaction with r­(CUG)^exp61^ was evaluated. Treatment with the MBNL1 binder led to a dose-dependent reduction in NanoBRET signal, suggesting that the NanoBRET assay can also report on r­(CUG)^exp^–MBNL1 complex disruption by binding to MBNL1 (Figure S3).

To add further rigor in the establishment of the assay, the effect of a CAG16 Gapmer that induces RNase H degradation of the r­(CUG)^exp^ mRNA was also measured in the HeLa480 NanoBRET assay. A dose dependent reduction of the NanoBRET signal was observed after transfection and overnight incubation with increasing concentrations of the Gapmer, with a maximal effect at 0.1 μM where the signal was reduced from 34 ± 0.4 mBU to 18 ± 1 mBU (where background BRET is 17 ± 0.4 mBU as determined from WT HeLa cells; *p* < 0.0001; Figures 3D and 3E). The observed reduction in the NanoBRET signal can be traced to the degradation of r­(CUG)^exp^, as a dose dependent reduction in r­(CUG)_480_ expression levels was observed by allele-specific RT-qPCR;^53^ whereas r­(CUG)_0_ (WT allele) abundance was unaffected (Figure 3D). At the highest Gapmer concentrations (50 nM and 100 nM), r­(CUG)^exp^ levels were reduced to near or below the detection threshold, indicating effective degradation by RNase H. Correspondingly, the NanoBRET signal was reduced to 18 ± 0.6, representing the minimum observed signal. This reduction aligns closely with the signal expected in wild-type (WT) cells, which lack r­(CUG)^exp^ RNA, suggesting that the NanoBRET assay reflects a complete release of MBNL1 from sequestration when r­(CUG)^exp^ RNA is undetectable. Indeed, a strong correlation between NanoBRET signal intensity and r­(CUG)^exp^ levels in HeLa480 cells treated with the CAG Gapmer was observed, with Pearson correlation coefficient of 0.98. These findings establish a direct relationship between NanoBRET signal and r­(CUG)^exp^ abundance, reinforcing the utility of the NanoBRET assay as a quantitative tool to measure both the presence of toxic RNA and the effectiveness of interventions aimed at reducing its levels. The concordance between the NanoBRET signal and the abundance of the repeat expansion further underscores the assay’s robustness in monitoring r­(CUG)^exp^–MBNL1 interactions and the bioactivity of small molecules that prevent or inhibit the toxic RNA–protein complex.

As aforementioned, one hallmark of DM1 is the formation of nuclear r­(CUG)-positive foci, composed of r­(CUG)^exp^ bound to various RBPs including MBNL1.^62^ To study whether the reduction in NanoBRET signal observed in HeLa480 cells upon treatment with CAG25 Vivo-Morpholino reports on the r­(CUG)^exp^–MBNL1 RNA–protein complex, foci formation was measured by single molecule fluorescence *in situ* hybridization (smFISH). Typically, foci formation in repeat expansion disorders employ a dye-labeled oligonucleotide complementary to the RNA.^59^ However, in the NanoBRET assay, competition between the ASO and the FISH probe would result in a false positive; that is, the foci are still present but the ASO impedes binding of the FISH probe. We therefore designed a series of smFISH probes complementary to both alleles, both mutant and wild type, in the HeLa480 cell line (Table S3).

As expected, in wild type (untransfected) HeLa480 cells, endogenous MBNL1 (using an anti-MBNL1 antibody) colocalized with r­(CUG)^exp^ (by smFISH) in the nucleus (Figure S4). Treatment with 10 μM of CAG25 Vivo-Morpholino significantly reduced the number and intensity of nuclear foci formed by endogenous MBNL1, as indicated by the diminished overlapping signals in the merged channels (Figure S4). This reduction was accompanied by a shift in MBNL1 localization from discrete nuclear foci to a more diffuse nuclear distribution. In cells expressing MBNL1–NanoLuc, the localization of the fusion protein was assessed by using an anti-NanoLuc antibody. In the absence of the Vivo-Morpholino, MBNL1–NanoLuc colocalized with r­(CUG)^exp^ in nuclear foci (with some cytoplasmic distribution; Figure 2A), which were markedly reduced upon treatment with 10 μM of CAG25 Vivo-Morpholino (Figure 2B; *p* < 0.0001). Thus, the NanoBRET signal and its reduction by the two ASOs can be traced to the extent of complex formation between MBNL1 and r­(CUG)^exp^ and validates the assay as a functional readout for RNA–protein interactions causative of DM1.

To assess whether repeat module number influences NanoBRET performance, three plasmid constructs encoding different numbers of interrupted repeats were cotransfected into HeLa480 cells along with MBNL1–NanoLuc and MBNL1–HaloTag. The three plasmids, dubbed DT960, DT480, and DT240, harbor 48, 24, and 12 interrupted [(CTG)_20_(CTCGA)] modules, respectively.^63^ Under optimized assay conditions, all three constructs generated comparable NanoBRET windows (Figure S5A–C), demonstrating that signal amplitude is not linearly dependent on the number of interrupted modules present on a single RNA molecule. These transiently transfected systems produced window sizes ranging from 13.5 mBU to 25 mBU and express higher levels of r­(CUG)^exp^ than HeLa480 (Figure 3B and Figure S5D).

An intent for the development of the HeLa480 NanoBRET assay was to enable screening of small molecules that inhibit r­(CUG)^exp^–MBNL1 complex formation in live cells. We therefore measured the *Z*-factor^64^ for the assay in 96-well plates using a 10 μM dose of CAG25 Vivo-Morpholino. This statistical parameter measures the suitability of an assay for high throughput screening (HTS) and ranges in value from 0 to 1, where a *Z*-factor ≥ 0.5 is considered suitable for HTS.^64^ The *Z*-factor for this NanoBRET assay is 0.63 and is therefore acceptable for HTS (Figure S6).

### The r­(CUG)^exp^–MBNL1 NanoBRET Assay Identifies Small Molecules That Inhibit Complex Formation in Live Cells

The NanoBRET assay was employed to determine if a panel of small molecules (*n* = 72) could reduce or inhibit r­(CUG)^exp^–MBNL1 complex formation. These small molecules were selected based on molecular fingerprinting^65^ of structural features and physicochemical properties of known RNA-binding molecules to query a large chemical library (∼1 billion compounds). The resulting 1957 small molecules were further refined by cluster analysis (RDKit: Open-source cheminformatics, https://www.rdkit.org) and predicted solubility^66^ to provide the 72 compounds that comprise the panel. Uniform Manifold Approximation and Projection (UMAP)^67^ analysis to define the chemical space of the panel verified that the selected molecules retained favorable drug-like properties, as compared to molecules in DrugBank.^68^ This analysis confirmed that many exhibited good solubility, bioavailability, and pharmacokinetic properties, with an average quantitative estimate of drug-likeness (QED)^69^ score of 0.68 ± 0.015 (standard error).

The small molecules were screened in triplicate for inhibiting r­(CUG)^exp^–MBNL1 complex formation in HeLa480 at a dose of 50 μM, affording ten small molecules that reduced the NanoBRET signal by >σ from the mean of all small molecules assayed (Figure 4). These small molecules were generally well-tolerated in cells, as assessed by cell viability measurements (>80% for all hit molecules; Figure S7), indicating minimal cytotoxic effects at the screening concentration. Interestingly, the hit small molecules could be classified into two chemotype families, **A** (*n* = 6) and **B** (*n* = 4) (Figure 4). Dose response studies verified that each small molecule advanced from the primary screen reduced NanoBRET signal with a range of potencies; some small molecules reduced the NanoBRET signal to a similar extent as the ASO (10 μM) at the 20 μM or 50 μM dose (Figure 5A). The most potent small molecules in this assay were **A5**, **A6**, and **B4**, with IC_50_ values of 16 ± 2 μM, 27 ± 3 μM, and 20 ± 2 μM, respectively. In contrast, the least potent compounds, **A1** and **B2**, showed activity only at the highest dose of 50 μM.

**Figure 4:**
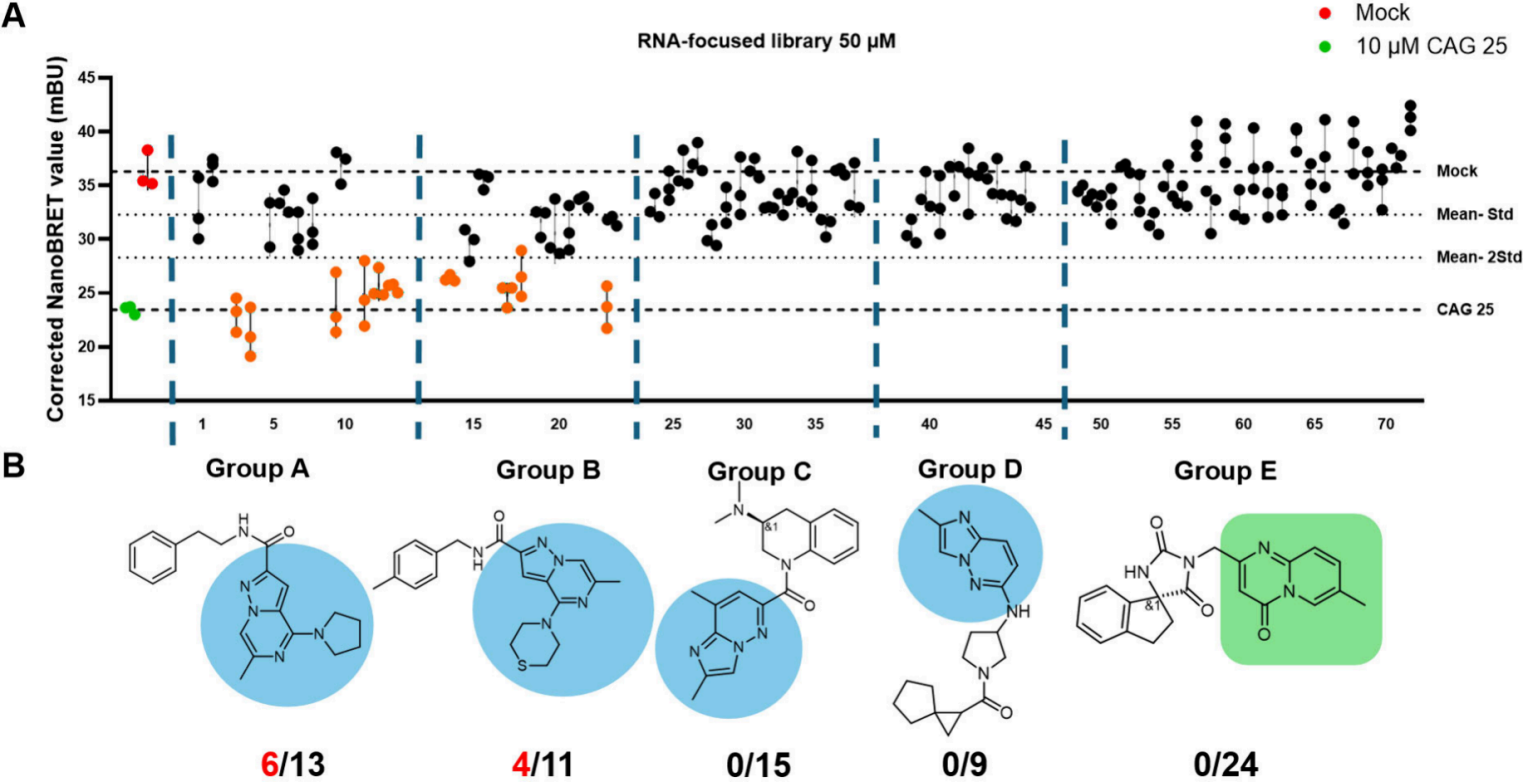
**High-throughput screening of small molecules for inhibition of r­(CUG)** ^**exp**^ **–MBNL1 complex formation in the NanoBRET assay**. (A) Small molecules from an RNA-focused library were screened in triplicate in the HeLa480 NanoBRET assay at a concentration of 50 μM to identify inhibitors of the r­(CUG)^exp^–MBNL1 complex. The corrected NanoBRET values (NanoBRET signal of treated samples minus NanoBRET signal of control) are plotted for all screened compounds. Dashed lines indicate Mean and Mean ± σ. Compounds that reduced the NanoBRET signal by > σ from the mean are highlighted in orange. Mock-treated controls (red) and CAG25 Vivo-Morpholino-treated cells (green) are included for comparison. This screen identified ten small molecules that reduced NanoBRET signal by σ, suggesting disruption of the r­(CUG)^exp^–MBNL1 interaction. (B) The hit small molecules identified from the NanoBRET assay can be classified into two distinct chemotype families: chemotype A (*n* = 6 hits) and chemotype B (*n* = 4 hits), as indicated in red. Groups C, D, and E contained no active small molecules under the assay conditions. The structural features highlighted (blue and green regions) represent the conserved scaffolds that distinguish the chemotype families.

**Figure 5:**
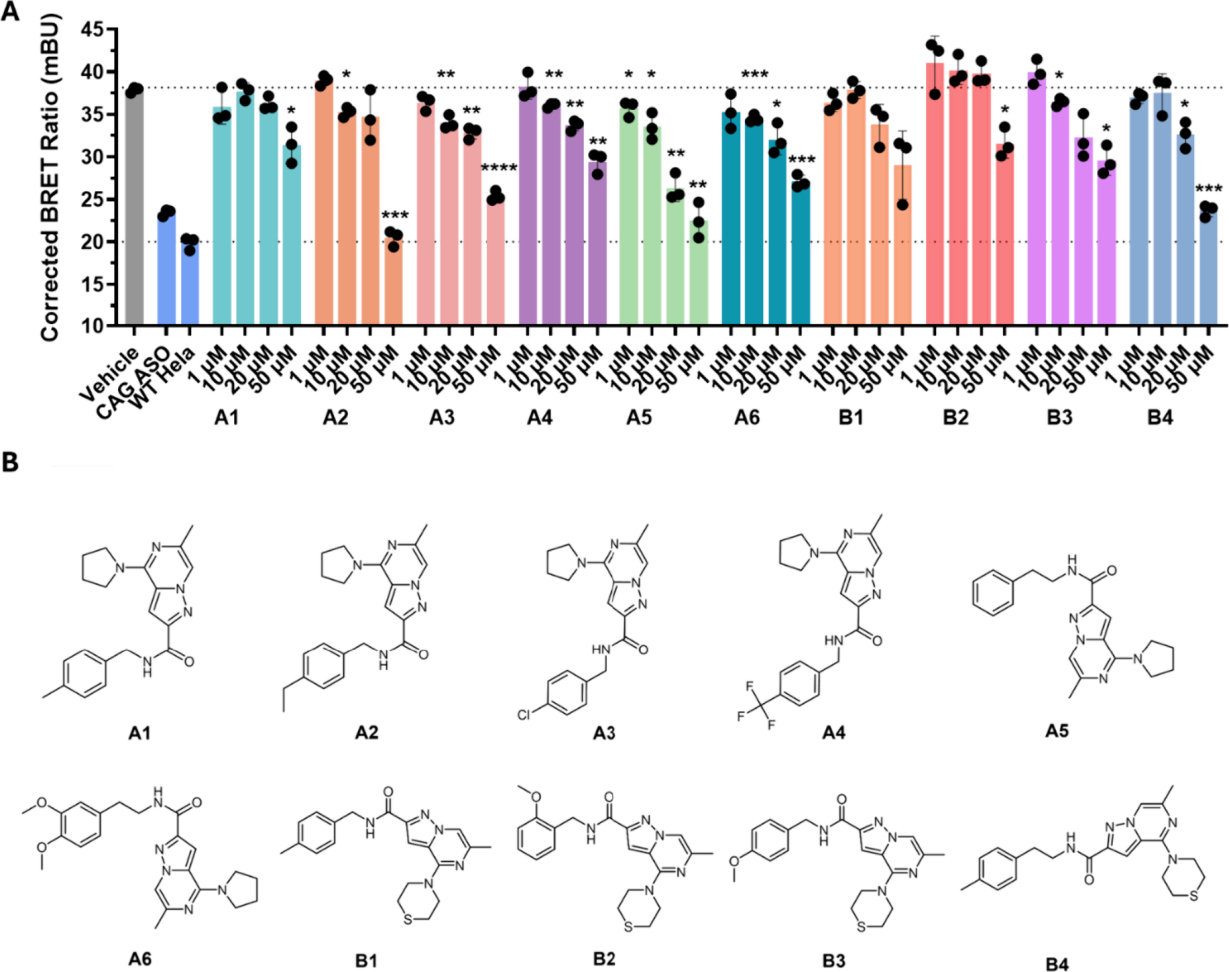
**Dose–response analysis and chemical structures of small molecules that disrupt r­(CUG)** ^**exp**^ **–MBNL1 complex formation in the NanoBRET assay**. (A) Dose dependent reduction of the corrected BRET ratios (mBU) for small molecules **A1**–**A6** and **B1**–**B4**, identified from the primary screening assay. Corrected BRET ratios for vehicle-treated and CAG25 Vivo-Morpholino-treated (ASO)-treated cells and wild type HeLa cells are included for comparison. Data are reported as mean ± standard deviation (*n* = 3 biological replicates). Statistical significance was determined relative to untreated controls using a two-tailed unpaired Student’s *t* test with significance thresholds: *, *p* < 0.05; **, *p* < 0.01; ***, *p* < 0.001; ****, *p* < 0.0001. (B) Chemical structures of the top ten small molecules identified from the primary screening assay that were studied in dose response.

### Small Molecules That Reduce the NanoBRET Signal Inhibit the r­(CUG) Repeat–MBNL1 Complex *in Vitro*

To assess if there is a correlation between inhibition of the r­(CUG)^exp^–MBNL1 complex in the cellular NanoBRET assay and *in vitro*, a previously developed time-resolved fluorescence resonance energy transfer (TR-FRET) assay was employed.^70^ In brief, complex formation is measured using biotinylated r­(CUG)_12_ and MBNL1–His_6_. By using streptavidin-XL665 and an anti-His_6_-Tb-labeled antibody, a FRET signal is generated upon complex formation. Therefore, a reduction in FRET is observed if a small molecule inhibits the interaction between the r­(CUG) repeats and MBNL1. All ten small molecules demonstrated dose-dependent disruption of the r­(CUG) repeat–MBNL1 complex *in vitro*, while the three previously identified inactive compounds showed no such effect (Figure S8 and Table S4). However, the rank order of compound potency in the TR-FRET (*in vitro*) and NanoBRET (cellular) assays exhibited substantial discrepancies, rather than following a parallel trend (Table S4). For instance, while **A1** and **A6** exhibited highest potency in the TR-FRET assay (IC_50_’s of 14 ± 3 μM and 18 ± 2 μM, respectively) and both reaching ∼100% disruption of complex formation at the highest tested concentration (50 μM), only **A6** retained comparable potency in the cellular NanoBRET (IC_50_ of 27 ± 3 μM). In contrast, **A1**’s potency was markedly reduced in the cellular assay, with activity only observed at the highest dose (50 μM) tested in cells. This difference could be due to cell permeability or target specificity. These results further highlight the strength of the NanoBRET assay, which maintains the physiological environment, making it especially valuable for identifying small molecules capable of alleviating RNA-mediated cellular pathologies.

### Small Molecules Inhibit the r­(CUG)_12_–MBNL1 Complex by Binding to the RNA

In our NanoBRET and TR-FRET assays that assess r­(CUG)^exp^–MBNL1 complex formation, reduced complex formation could be observed if the small molecule binds to either the RNA or the protein. The latter is likely undesirable as small molecule binding to MBNL1 could inhibit binding to its canonical substrates and exacerbate alternative splicing defects.^61^ Therefore, differential scanning fluorimetry (DSF) was employed to determine whether the compounds act primarily by binding the RNA or the protein. Using a FAM–r­(CUG)_10_–BHQ RNA construct, eight of the ten compounds dose dependently induced thermal shifts, whereas **B1** and **B2** exhibited no measurable shift at 100 μM. Among the small molecules with evidence of binding, **A6** produced the largest change in melting temperature (*T*_m_), Δ*T*_m_ = 1.2 °C (*p* < 0.0001; Figure S9A). The DSF assays conducted with purified MBNL1 protein and SYPRO Orange revealed no measurable thermal shift at 100 μM for nine of the ten compounds, where only **A1** induced a modest shift (Δ*T*_m_ = 1.0 °C; *p* = 0.0009). As a positive control, the previously reported MBNL1-binding compound produced a dose dependent thermal shift, reaching a maximum Δ*T*_m_ = 1.9 °C at 100 μM (*p* < 0.0001), confirming the assay’s sensitivity (Figure S9B). Collectively, these findings support that most tested compounds disrupt the r­(CUG)^exp^–MBNL1 complex primarily through direct RNA binding rather than interaction with MBNL1 protein.

We also studied the binding of the ten small molecules that reduced NanoBRET signal to r­(CUG) repeats by nuclear magnetic resonance (NMR) spectrometry. Binding is inferred from changes in the chemical shifts, line broadening, or intensity of the imino signals, which reflect changes in the local RNA environment caused by its interaction with a small molecule, arising from several possible mechanisms such as disruption of hydrogen bonding and/or conformational changes.^71^ The effect of each small molecule on the imino proton spectrum of an RNA harboring two 1 × 1 nucleotide UU internal loops present in r­(CUG)^exp^ was measured in NMR buffer (5 mM KH_2_PO_4_/K_2_HPO_4_, pH 6.0, and 50 mM NaCl) (Figures S10–S20). The molecules were classified into two distinct groups: (i) two compounds (**B1** and **B2**) exhibiting very weak binding, as indicated by minimal alterations in peak intensities of the imino protons within the 5′CUG/3′GUC internal loops; and (ii) eight compounds (**A1**–**A6**, **B3**, and **B4**) demonstrating moderate binding, characterized by more pronounced changes in peak intensities and/or chemical shifts of the imino proton resonances in the internal loops. For most compounds, perturbations were localized primarily to the UU internal loops with minimal disruption to the canonically base-paired regions or the hairpin tetraloop (Figures S10–S20). This observation suggests that these molecules predominantly bind to the internal loop without broadly affecting regions outside the 5′CUG/3′GUC repeat. Notably, the weak binding exhibited by **B1** and **B2** in the NMR assay correlates with their lack of detectable thermal shift in DSF experiments (Figure S9B).

Addition of **A6** (alleviates DM1-associated splicing defects in patient-derived myotubes, *vida infra*) to the RNA repeat led to exchange broadening and shifting of resonances, notably around the 5′CUG/3′GUC internal loop (U5/U19 and U8/U22). At a 2:1 **A6**: RNA ratio, the U5/U19 H3, U8/22 H3, G6/G20 H1 and G9/G23 H1 (a nucleotide in one loop’s closing GC base pair) resonances broadened and peak intensity was reduced, indicating the compound’s interaction with the U/U internal loops. Collectively, our findings demonstrate the ten small molecules identified from the NanoBRET assay engage r­(CUG)^exp^ primarily at the internal loops, albeit to different extents as indicated by changes in imino proton spectra, providing an avenue for developing ligands that selectively modulate pathogenic RNA structures while minimizing off-target effects on MBNL1 or other RNAs.

### Small Molecules Rescue Pre-mRNA Splicing Defects and Formation of Foci in DM1 Myotubes

The ten small molecules with activity in the NanoBRET assay and three control compounds that were inactive (representing a different scaffold; Figures 6B and S21A) were studied for improving DM1-associated cellular phenotypes in patient-derived myotubes - rescue of alternative pre-mRNA splicing defects (Figure 6A) and formation of nuclear foci. For these studies, DM1 patient-derived or wild type fibroblasts, forced into myogenic differentiation by doxycycline-induced expression of myoblast determination protein 1 (MYOD1),^72^ were employed. Cells were treated with the compound of interest during myogenic differentiation, followed by measuring rescue of the MBNL1 exon 5 alternative splicing defect observed in this cell line.^43,50,73^ After myogenic differentiation, the percentage of exon 5 inclusion in DM1 patient-derived cells was 31 ± 1% while the percentage of inclusion in wild type myotubes was 5 ± 1%. As expected,^72^ this pre-mRNA splicing defect was more severe in DM1 patient-derived myotubes than in the patient-derived fibroblasts, that is without differentiation (31 ± 1% vs 3 ± 0.2%). The three negative control compounds that were inactive in the NanoBRET assay were unable to rescue the *MBNL1* exon 5 splicing defect at a 50 μM dose (Figures S21 and S22A). The inactivity of these three molecules in both the NanoBRET assay and functional splicing rescue experiments suggest that false positives are not significantly reported by the NanoBRET assay. However, additional orthogonal validations are still required to confirm compound activity.

**Figure 6:**
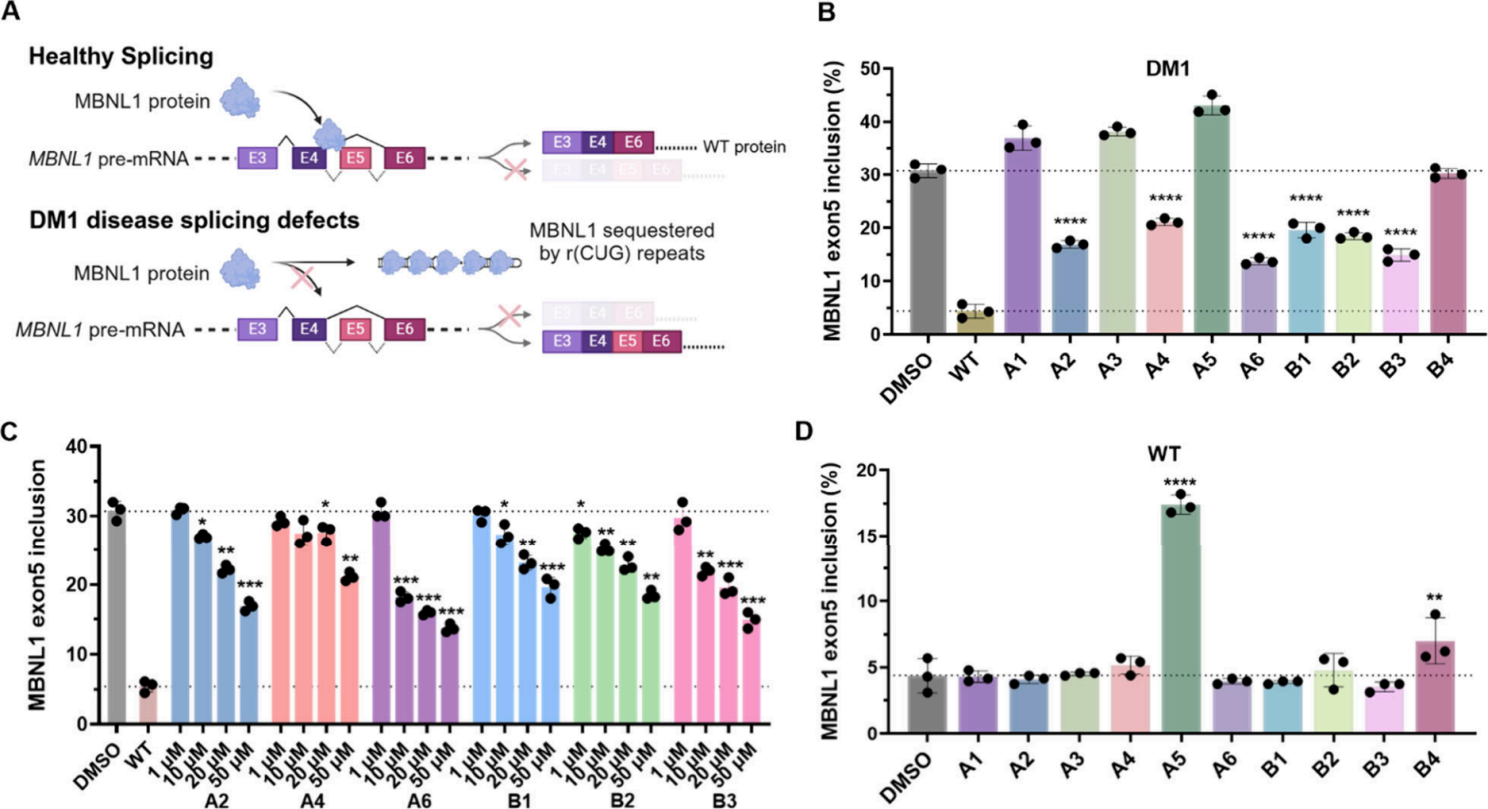
**Effect of compounds on** ***MBNL1*** **exon 5 alternative splicing in DM1 and wild type myotubes by small molecules**. (A) Schematic representation of *MBNL1* exon 5 alternative splicing in healthy and DM1 myotubes. In healthy cells, MBNL1 protein regulates the alternative pre-mRNA splicing of its own exon 5. In DM1-affected cells, sequestration of MBNL1 by toxic r­(CUG)^exp^ causes widespread deregulation of splicing events, including aberrant inclusion of exon 5. (B) MBNL1 exon 5 splicing inclusion levels were measured in DM1 patient-derived myotubes upon treatment with ten small molecules identified from the NanoBRET assay. Compounds were tested at a single dose (50 μM) during myogenic differentiation, and inclusion levels were compared to untreated DM1 myotubes and untreated WT myotubes. Rescue of MBNL1 exon 5 inclusion was observed in patient-derived myotubes upon treatment with **A2**, **A4**, **A6**, **B1**, **B2**, and **B3**. (C) Dose–response analysis of the six active compounds identified in (B) in DM1 myotubes. Cells were treated with increasing concentrations (1–50 μM) of the compound of interest during differentiation, and exon 5 inclusion levels were measured by end-point RT-PCR. All six compounds showed dose-dependent rescue of MBNL1 exon 5 inclusion. (D) MBNL1 exon 5 splicing inclusion levels in wild type (WT) myotubes treated with compounds **A2**, **A4**, **A6**, **B1**, **B2**, and **B3** (50 μM). For all panels, data are reported as mean ± standard deviation (*n* = 3 biological replicates). Statistical significance was determined relative to untreated controls using a two-tailed unpaired Student’s *t* test with significance thresholds: *, *p* < 0.05; **, *p* < 0.01; ***, *p* < 0.001; ****, *p* < 0.0001.

In contrast, six of the ten small molecules active in the NanoBRET assay, **A2**, **A4**, **A6**, **B1**, **B2**, and **B3**, rescued MBNL1 exon 5 alternative splicing when DM1 myotubes were treated with 50 μM compound without affecting MBNL1 protein expression levels (Figure 6B and Figure S23A,B). While most compounds were well tolerated (Figure S23C), **A3** and **A5** affected DM1 myotube viability, which paralleled a worsening of MBNL1-dependent splicing defects; similarly **A5** caused toxicity in wild-type cells (Figure S23C,D). Inactive compounds **A1** (*p* < 0.0001), **A3** (*p* < 0.0001), **A5** (*p* < 0.0001), and **B4** (*p* = 0.0007) significantly reduced *MyoD* transcript levels in DM1 patient-derived cells (Figure S23E). Since *MyoD* is a key regulator of myogenic differentiation,^72^ its downregulation suggests that the fibroblast-to-myoblast conversion process was affected and a potential off-target.

Among the active compounds, **A6** rescued the splicing defect to the greatest extent, where exon 5 is included 14 ± 0.6%, or an ∼66% improvement when comparing the percent exon inclusion in untreated cells (31 ± 1%) and wild type cells (5 ± 1%) (Figure 6B). Additional studies showed that **A2**, **A4**, **A6**, **B1**, **B2**, and **B3** dose dependently rescued MBNL1 splicing, with **A6** showing the most potent effects with an IC_50_ of ∼15 μM (Figures 6C and S22B). Notably, the small molecules (50 μM) had no effect on MBNL1 exon 5 splicing in wild type myotubes nor did they affect *MAP4K4* exon 22a alternative splicing, a NOVA-regulated splicing event,^74,75^ in either DM1 or WT myotubes (Figures 6D, S22C, and S24A).

To gain insight into how the small molecules might rescue splicing defects in patient-derived myotubes, that is if they function by binding to r­(CUG)^exp^ and displacing MBNL1, their effects on *DMPK* transcript levels (would suggest a transcriptional inhibitor) were measured. The compound **A2** reduced *DMPK* transcript levels by >40%, while **B1** caused a decrease of >20%, suggesting that it may act, at least partially, as transcriptional inhibitors (Figure S24B). In contrast, **A4**, **A6**, **B2** and **B3** did not significantly affect *DMPK* transcript levels, ruling out transcriptional inhibition as their mechanism of action (Figure S24B). None of the compounds, including **A2**, reduced *MyoD* abundance as measured by RT-qPCR (Figure S23E), indicating that they do not induce dedifferentiation of DM1 myotubes. Notably, compounds **B1** and **B2**, which exhibit no or weak RNA-binding affinity *in vitro*, rescue splicing defects in DM1 patient-derived cells and produce measurable effects in the NanoBRET assay, which may stem from an alternative mechanism rather than binding to the repeat expansion. For **B1**, this is likely due to the molecule acting as a transcriptional inhibitor.

Finally, smFISH [r­(CUG)^exp^] and an immunofluorescence assay (IFA; MBNL1) were used to evaluate whether the small molecules inhibit the r­(CUG)^exp^–MBNL1 complex in DM1 patient-derived myotubes, as evidenced by reduction of nuclear foci. All four compounds that did not reduce *DMPK* or *MyoD* abundance—**A4**, **A6**, **B2** and **B3**—significantly reduced the number and intensity of MBNL1-containing nuclear foci at 50 μM, supporting their ability to displace MBNL1 from r­(CUG)^exp^ RNA (Figures 7 and S25; *p* < 0.0001). Collectively, these findings suggest that while **A2** and **B1** may partially act through transcriptional inhibition, **A4**, **A6**, **B2**, and **B3** primarily function by directly targeting r­(CUG)^exp^ RNA, displacing MBNL1, and restoring its functional availability, thereby rescuing splicing defects in DM1 cells.

**Figure 7:**
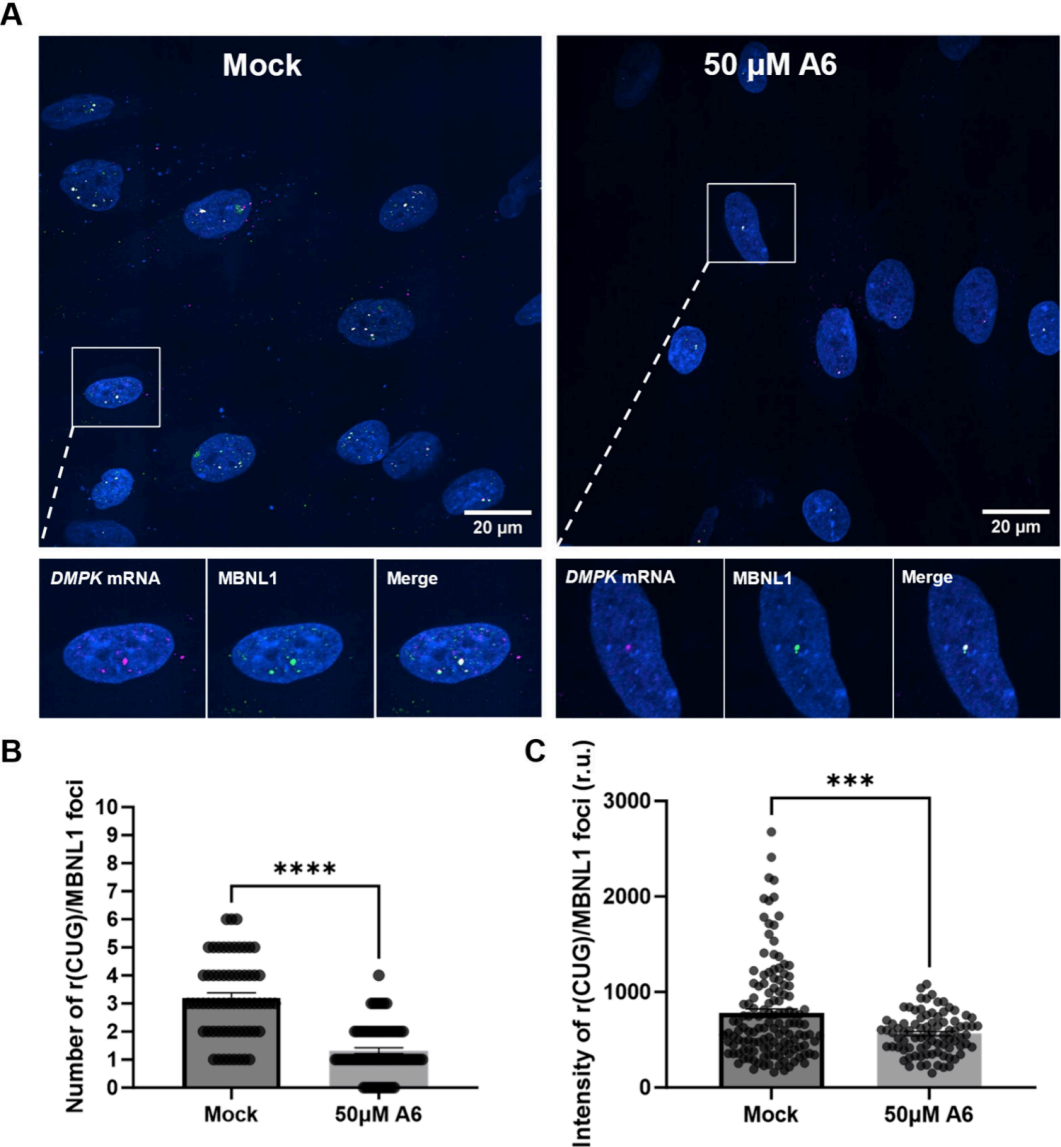
**Effect of A6 on formation of nuclear foci in DM1 patient-derived myotubes**. (A) Single-molecule RNA fluorescence *in situ* hybridization (smRNA-FISH) for *DMPK* CDS (magenta) and IFA for MBNL1 (green). Representative images of DM1 patient-derived myotubes treated with 50 μM of **A6** or vehicle (0.1% (v/v) DMSO). DAPI staining (blue) was used to visualize nuclei. Scale bar is 20 μm. (B) Quantification of r­(CUG)^exp^–MBNL1 foci number in the nuclei of DM1 myotubes (with 40 nuclei quantified/replicate; *n* = 3 biological replicates) as represented in (A). ****, *p* < 0.0001, as determined by Student’s *t* test. Data are reported as the mean ± SEM (C) Quantification of r­(CUG)^exp^–MBNL1 foci intensity in the nuclei of DM1 myotubes (with 40 nuclei quantified/replicate; *n* = 3 biological replicates) as represented in (A). ***, *p* < 0.001, as determined by Student’s *t* test. All intensity measurements are expressed in relative units (r.u.). Data are reported as the mean ± SEM.

As **A6** appeared the most promising of the small molecules that improve DM1-associated defects in myotubes, its binding affinity for r­(CUG)_2_, the same construct used in NMR studies, was measured. The fluorescence intensity of **A6** (100 nM) was measured as a function of RNA concentration, and the resulting curve was fit to afford a dissociation constant (*K*_d_) of 17 ± 2 μM (Figure S26A). In contrast, no significant binding of **A6** was observed when titrating **A6** with a fully paired RNA, indicating specificity for r­(CUG) repeats (Figure S26B). These results demonstrate that the NanoBRET assay is suitable for discovering small molecules with sufficient affinity (here, low μM) that can alleviate DM1-associated molecular pathology from model systems to patient-derived cell lines.

## Discussion

Resonance energy transfer (RET) techniques, both fluorescence (FRET) and bioluminescence (BRET), to study protein–protein interactions were developed to overcome the limitations associated with immobilizing one of the protein partners and with maintaining the natural fold of the cellular proteins in lysates. That is, cellular FRET and BRET assays allowed these interactions to be studied in live cells and under native conditions.^76–78^ With careful design, the first NanoBRET platform enabled the detection of transient interactions using tagged proteins expressed at levels comparable to endogenous expression as well as measuring the inhibitory activity of small molecules with therapeutic relevance.^48^ Shortly thereafter, the assay was extended to measure the binding affinity of tagged proteins for complementarily labeled antisense oligonucleotides and double stranded (ds)­RNAs in cells, the latter as proxies for cellular (canonical) nucleic acid substrates.^79^ Akin to the studies that developed RET techniques to study protein–protein interactions, the analogous assay to study RNA–protein interaction could overcome some of the limitations, for example immobilization of the RNA, time, number of steps, and potential nonphysiological interactions, of CLIP-type techniques.

It was observed that compound potencies did not always align across *in vitro* binding, TR-FRET, and cellular NanoBRET assays. For example, **A1** shows moderate binding to r­(CUG) repeats by NMR spectrometry and strong activity in the TR-FRET assay (IC_50_ = 14 ± 3 μM) but only shows weak activity in NanoBRET assay at highest concentrations (50 μM). These differences could be due to insufficient cellular permeability or target selectivity. Conversely, **B1** exhibits weak RNA affinity (*K*_d_ > 50 μM) and TR-FRET activity (IC_50_ > 50 μM) yet is active in the NanoBRET assay and rescues an alternative splicing defect, consistent with acting as a transcriptional inhibitor rather than a r­(CUG)^exp^ binder. In contrast, **A6** displays consistent activity across all *in vitro* and cellular assays, indicative of direct RNA engagement. These observations underscore the complexity of live-cell assays that are not captured by solution-phase binding measurements, for examples compound uptake, intracellular distribution and colocalization of the small molecule and target, as well as target specificity, among others. Consequently, integrating multiple orthogonal assays is essential to prioritize small molecules with both biochemical target engagement and cellular efficacy.

The NanoBRET assay developed and validated here measures formation of a toxic RNA–protein interaction that causes DM1 and the inhibition thereof by antisense oligonucleotides and small molecules. A hallmark of many microsatellite (RNA repeat expansion) disorders, such as DM1, is sequestration of RNA-binding proteins into foci, leading to RBP loss of function. The sequences of RNA repeat expansions differ as well as the repeat unit (triplet–hexanucleotide repeats) and unsurprisingly different RBPs are sequestered by each sequence, although there are some commonalities. For example, MBNL1 is also sequestered by r­(CCUG)^exp^ (myotonic dystrophy type 2; DM2)^80^ and r­(CAG)^exp^ repeats that cause spinocerebellar ataxia 3 (SCA3).^81^ Although the main mechanism of toxicity of the hexanucleotide repeat expansion that causes C9orf72-associated amyotrophic lateral sclerosis and frontotemporal dementia is aberrant translation of the repeats in dipeptide repeat proteins,^82–85^ the RNA repeat expansion also sequesters heteronuclear ribonucleoprotein H (hnRNP H), which causes alternative pre-mRNA splicing defects.^86^ Therefore, similar assays could be developed for other RNA repeat expansion disorders and enable high throughput screening of small molecules or other modalities that inhibit RNA–protein interactions that could then be studied in advanced cellular models. As the NanoBRET assay is sensitive to changes in RNA abundance, demonstrated by CAG Gapmer (Figure 3D), it should be compatible with small molecules that degrade the target directly^87^ or that induce cleavage by RNase recruitment.^88–90^

Various methods have been developed to image RNA, in particular aptamers that upon binding a ligand produce a fluorescent signal. These aptamers are fused to an RNA target of interest, producing a fluorescent signal upon delivery of the exogenous ligand to the cell.^91–93^ These RNA-aptamer fusions have been used to study cellular localization and small molecule binding,^91–94^ however, their fluorescence signals are relatively weak and are measured by confocal microscopy. One could envision an RNA aptamer for the HaloTag, enabling the detection of hypothetically any RNA–protein interaction, where the protein would carry NanoLuc, an assay amenable to a high throughput format using a plate reader.

As noted above, previous studies have suggested that exons 5, 6, and 7 contribute to the nuclear localization of MBNL1.^56,57^ The constructs used herein lack exons 6 and 7, and our imaging studies in HeLa480 cells showed that the truncated MBNL1 proteins were distributed approximately equally between the nucleus (in foci) and cytoplasm, rather than a predominant nuclear localization. Despite this change in subcellular localization, NanoBRET was observed upon binding r­(CUG)^exp^. To study whether enhanced signal might be observed if the MBNL1 fusions were predominantly localized to the nucleus, MBNL1–NanoLuc and MBNL1–HaloTag fusion protein containing a nuclear localization sequence (NLS) were engineered. Although the modified proteins accumulated predominantly in the nucleus, quantitative NanoBRET measurements revealed no significant increase in the optimal signal window relative to the original nonNLS- constructs (13 mBU vs 13.5 mBU; Figure S27). These results demonstrated that the cytoplasmic fraction of the fusions contribute negligibly to background signal and enforced nuclear targeting does not confer a measurable performance advantage under the conditions tested.

## Conclusions

Herein, a NanoBRET-based platform is described that enabled interrogation of an RNA–protein interaction that causes the neuromuscular disorder myotonic dystrophy type 1. The platform was validated using an ASO, providing a signal window of ∼13.5 mBU, where the lowest NanoBRET ratio observed was similar to that in cells that do not express the target RNA. Furthermore, the assay is suitable for high throughput screening efforts with a *Z*-factor of 0.63. Indeed, the platform identified a cohort of drug-like small molecules that were carried forward to *in vitro* studies that demonstrated target binding and to studies in advanced cellular models that assessed rescue of a DM1-associated alternative pre-mRNA splicing defect and reduction of nuclear foci, two hallmarks of disease. It is envision that this platform can be extended to other RNA repeat expansion disorders and to other RNA–protein interactions, the latter if a suitable aptamer could be discovered that binds the HaloTag ligand. Given the dynamic and complex nature of RNA folding conducting screening campaigns in native cellular systems for RNA is important and this system could provide a streamlined way to do that.

## Materials and Methods

### Antisense Oligonucleotides That Target r­(CUG)^exp^.^95^

The CAG25 Vivo-Morpholino antisense oligonucleotide (5′-AGCA­GCAG­CAGC­AGCA­GCAG­CAGCA-3′) was purchased from Gene Tools, LLC. The CAG16 Gapmer antisense oligonucleotide, 5′-+C+A+G*C*A*G*C*A*G*C*A*G*C+A+G+C-3′ where + indicates an LNA residue and * indicates a phosphorothioate backbone, was acquired from Qiagen.

### Cell Culture: HeLa480 Cells

HeLa480 cells^53^ were maintained in Dulbecco’s Modified Eagle Medium (DMEM; Corning, catalog #15-017-CV) supplemented with 10% (v/v) fetal bovine serum (FBS; Gibco, catalog #12676029), 1% (v/v) Antibiotic-Antimycotic Solution (Corning, catalog #30-004-CI), and 1% (v/v) Glutagro (Corning, catalog #25-015-CI).^53^ Cells were cultured at 37 °C in a humidified atmosphere containing 5% CO_2_ and used at a passage numbers less than 20. Cells were tested for mycoplasma contaminations and determined to be mycoplasma-free before used in experiments.

### NanoBRET Assay: Determining the Optimal Placement of the Fusion (N- or C-Terminus) and Plasmid Ratios

To optimize the assay, HeLa480 cells were cotransfected with plasmids encoding MBNL1 fused to HaloTag and NanoLuc in various donor to acceptor DNA ratios in a 6-well plate using 8 μL of FuGENE HD Transfection Reagent (Promega, catalog #E2311) in 110 μL of Opti-MEM (Giboco, catalog #31985070), following the manufacturer’s protocol. The transfection mix was incubated for 10 min at room temperature before being added to the cells. After incubating the cells for 20 h, they were trypsinized from the surface, resuspended in an appropriate volume of assay medium (Opti-MEM + 4% (v/v) FBS), and counted using trypan blue (Corning, catalog #25-900-CI) and the Countess 3 Automated Cell Counter (Invitrogen, catalog #AMQAX2000) to determine cell viability and total cell number. The cell suspension was then diluted to the desired concentration and reseeded into white 96-well tissue culture plates (Corning, catalog #3903) where 100 μL of growth medium containing 200 nM HaloTag NanoBRET 618 Ligand (Promega, catalog #N1662) was added to each well, delivering 1.6 × 10^4^ transfected cells (affording ∼70% confluency). [Note: in each experiment, cells were delivered only in 100 μL of growth medium to three wells as no HaloTag ligand controls.] The cells were then incubated overnight at 37 °C with 5% CO_2_ to facilitate specific binding to the HaloTag fusion protein.

The following day, NanoLuc substrate and 20 μM extracellular NanoLuc inhibitor were added to the wells. For a 96-well plate, 2.5 mL of Opti-MEM were mixed with 25 μL of NanoLuc substrate (Promega, catalog #N1662) and 8.3 μL of extracellular NanoLuc inhibitor (Promega, catalog #N2160). Then, 25 μL of this mixture was added to each well, resulting in final concentrations of 1× NanoLuc substrate and 20 μM extracellular NanoLuc inhibitor in the assay medium and mixed at room temperature. The interaction between MBNL1 and r­(CUG)_480_ was measured by detecting the BRET signal using the GloMax Discover System (Promega, catalog #GM3000). Dual-filtered luminescence was collected with a 460/80 nm bandpass filter for the donor (NanoLuc protein) and a 610 nm long-pass filter for the acceptor (HaloTag ligand) using an integration time of 500 ms. The corrected NanoBRET ratio, expressed in milliBRET units (mBU), was calculated using eq 1:

1 $$\mathrm{Corrected NanoBRET Ratio}=\frac{\mathrm{HaloTag ligand}\;\left(\mathrm{Emission}\;610\;\mathrm{nm}/\mathrm{LP}\right)}{\mathrm{Donor Luminescence}\;\left(\mathrm{Emission}\;450\;\mathrm{nm}/\mathrm{BP}\;80\;\mathrm{nm}\right)}-\frac{\mathrm{No Ligand Control}\;\left(\mathrm{Emission}\;610\;\mathrm{nm}/\mathrm{LP}\right)}{\mathrm{Donor Luminescence}\;\left(\mathrm{Emission}\;450\;\mathrm{nm}/\mathrm{BP}\;80\;\mathrm{nm}\right)}$$

NanoBRET inhibition rate was calculated using eq 2:

2 $$Inhibition\;rate=\frac{Ratio_{DMSO}-Ratio_{sample}}{Ratio_{DMSO}-Ratio_{WT}}\times 100\%$$

In all subsequent assays, a ratio of 1:50 MBNL1–HaloTag:MBNL1–NanoLuc plasmids (2.5 μg HaloTag and 50 ng NanoLuc) was used as described.

### Validation of the NanoBRET Assay Using a CAG25 Vivo-Morpholino

The CAG25 Vivo-Morpholino was added directly to the cell culture medium (without the need for a transfection reagent) at the recommended final concentration of 10 μM, according to the manufacturer’s guidelines. It was dissolved in RNase-free water and stored at room temperature before use. The Vivo-Morpholino was added 4 h after the cells had been reseeded into the 96-well plate. Cells were then incubated with the Vivo-Morpholino for 16 h before NanoBRET signal detection.

### Suitability of the NanoBRET Assay for HTS: *Z*-Factor

To evaluate the suitability of the NanoBRET assay for high-throughput screening (HTS), the *Z*-factor was calculated. The CAG25 Vivo-Morpholino was added to the cells in a 96-well plate format at a final concentration of 10 μM. Specifically, half of the plate was treated with Morpholino, while the other half served as mock-treated controls. After treatment, cells were incubated overnight at 37 °C in a 5% CO_2_ atmosphere, prior to measuring NanoBRET as described in “NanoBRET Assay: Determining the Optimal Placement of the Fusion (N- or C-Terminus) and Plasmid Ratios”.

The Corrected NanoBRET ratio was calculated as shown in eq 1, which normalizes for nonspecific luminescence and fluorescence signals. The *Z*-factor, a statistical measure of assay quality, was calculated using eq 3:^64^

3 $$Z^{\prime }=1-\frac{3\left(\sigma_{sample}+\sigma_{control}\right)}{\left|\mu_{sample}-\mu_{control}\right|}$$

### Gapmer Oligonucleotide Transfection

Plasmids encoding MBNL1 fusion proteins were first transfected into HeLa480 cells cultured in 6-well plates (∼80% confluency; as described in “NanoBRET Assay: Determining the Optimal Placement of the Fusion (N- or C-Terminus) and Plasmid Ratios”). Approximately 24 h later, the transfected cells were reseeded into 96-well plates (Corning, catalog #3903) at a density of 1.6 × 10^4^ cells per well in 90 μL of growth medium, affording an approximate confluency of ∼70%. The cells were incubated at 37 °C for 4 h to allow adherence to the plate. Next, the CAG16 Gapmer was diluted to the indicated concentrations along with Lipofectamine RNAiMAX (0.3 μL per well, Invitrogen, catalog #13778150) in 10 μL of Opti-MEM (Gibco, catalog #31985070) and incubated for 5 min at room temperature to form the transfection complex. The transfection cocktail was added directly to the cells in culture medium. The cells were then incubated (37 °C, 5% CO_2_) overnight prior to downstream analysis.

### Validation of the NanoBRET Assay Using a CAG Gapmer

To measure activity in the NanoBRET assay, cells were grown and treated as described in “Gapmer Oligonucleotide Transfection” followed by measuring BRET as described in “NanoBRET Assay: Determining the Optimal Placement of the Fusion (N- or C-Terminus) and Plasmid Ratios”.

To measure r­(CUG)_480_ abundance, HeLa480 cells were plated in 12-well plates as transfected with the CAG16 Gapmer as described in “Gapmer Oligonucleotide Transfection”. After incubating overnight, total RNA was harvested using a Zymo Research Quick-RNA Mini Prep Kit per the manufacturer’s recommended protocol including the on-column DNase I digestion. Approximately 500 ng of total RNA was reverse transcribed with a qScript cDNA synthesis kit in 20 μL total reaction volume (Quanta BioSciences) per the vendor’s recommended protocol. QPCR amplification was carried out on QuantStudio 5, 384-well Block Real-Time PCR System (Applied Biosystems) by using TaqMan Universal PCR master mix with HT_Forward (900 nM) and HT_Reverse (900 nM) primers and custom HT_Probe1 or HT_Probe2 fluorescent probes (250 nM) (Table S2).^53^ HT_Probe1 was also used with HT primer sets to detect the expression of r­(CUG)^exp^ from DT240, DT480 and DT960 plasmids. Data were analyzed by using the comparative (ΔΔC_T_) method.^96^ The abundance of r­(CUG)_480_ and r­(CUG)_0_ was normalized to GAPDH and presented as relative mRNA levels by comparing the Gapmer to a scrambled ASO.^53^

### Validation of the NanoBRET Assay by Assessing smFISH/Foci

Single-molecule fluorescence *in situ* hybridization (smFISH) probes targeting *DMPK* exons 11–15 mRNA were designed using Stellaris probe design software (Biosearch Technologies), ensuring specificity and optimal hybridization efficiency. The sequences of these probes are provided in Table S3.

HeLa480 cells were cultured to approximately 80% confluency in 12-well plates containing glass-bottom wells. Cells were subsequently fixed in 4% (w/v) paraformaldehyde (PFA) for 10 min at 37 °C, and permeabilized by incubating with 75% (v/v) ethanol overnight at 4 °C. Following permeabilization, the ethanol was removed. For HeLa480 cells transfected with MBNL1 fusions, the cells were incubated with Anti-NanoLuc Monoclonal Antibody (Promega, catalog #N7000), diluted 1:1000 in 1× PBS, at 4 °C overnight. For untransfected HeLa cells and to image the localization of endogenous MBNL1, the cells were incubated with Anti-MBNL1Monoclonal Antibody (EMD Millipore, catalog #MABE70). Following incubation with the primary antibody, the cells were washed three times with 1× PBS (5 min each wash) and incubated with the secondary antibody, Alexa Fluor 488 goat antimouse IgG (H+L) (invitrogen, catalog #A-11001, 1:2000 dilution in 1× PBS) for 2 h at room temperature in the dark. Subsequently, *DMPK* smFISH probes (100 nM each) conjugated with ddUTP-Atto550 dye (Axxora, catalog #JBS-NU-1619-550) were prepared in 1× Hybridization Buffer (2× SSC Buffer, 10% (w/v) dextran sulfate, and 10% (v/v) deionized formamide). The probes were incubated with the cells at 37 °C in a humidified chamber overnight. After hybridization, the cells were washed three times with 2× SSC Buffer at 37 °C to remove unbound probes. Finally, the cells were mounted with 20 μL ProLong Gold Antifade Mountant with DNA Stain DAPI (invitrogen, catalog #P36931) for nuclear staining and imaged using LSM 980 Airyscan 2 Laser Scanning Confocal Microscope (Zeiss).

### Screening Small Molecules in the Optimized NanoBRET Assay

To identify small molecules that disrupt the sequestration of MBNL1 by r­(CUG)^exp^, a high-throughput NanoBRET screening assay was performed in HeLa480 cells. Cells were transfected with MBNL1–HaloTag and MBNL1–NanoLuc constructs at optimized ratios, as described in “NanoBRET Assay: Determining the Optimal Placement of the Fusion (N- or C-Terminus) and Plasmid Ratios”.

Approximately overnight post-transfection, cells were reseeded into 96-well plates (Corning, catalog #3903) at a density of 1.6 × 10^4^ cells per well in 100 μL of growth medium, affording an approximate confluency of ∼70%. The cells were incubated at 37 °C for 4 h to allow adherence before small molecules were added directly to the culture medium at their respective final concentrations. Cells were then incubated with compounds for 16 h prior to downstream analysis. The Corrected NanoBRET ratio and inhibition rate were calculated as described in “NanoBRET Assay: Determining the Optimal Placement of the Fusion (N- or C-Terminus) and Plasmid Ratios”.

To assess potential cytotoxic effects of the screened compounds, cell viability was measured using the CellTiter-Glo 2.0 Cell Viability Assay (Promega, catalog #G9242) following NanoBRET assay readout. After measuring NanoBRET, CellTiter-Glo 2.0 Reagent was equilibrated to room temperature and added to each well at a 1:1 ratio to the volume of culture medium (e.g., 125 μL of reagent for 125 μL of medium per well in a 96-well plate). The plate was then shaken at 500–700 rpm, followed by a 30 min incubation at room temperature to allow cell lysis and quench the NanoLuc signal. After incubation, total luminescence was recorded using the GloMax Discover System following the CellTiter-Glo protocol. Luminescence (RLU) values from vehicle-treated controls were compared to compound-treated wells to determine the effect of compound treatment on cell viability.

### *In Vitro* r­(CUG) Repeat–MBNL1 Displacement Assays.^97^

Complex formation between r­(CUG) repeats and MBNL1 *in vitro* was measured using a previously reported time-resolved fluorescence resonance energy transfer (TR-FRET) assay.^59^ Briefly, 5′-biotinylated r­(CUG)_12_ was folded by heating at 95 °C for 2 min in 1.5× TR-FRET Folding Buffer (30 mM HEPES-NaOH, pH 7.5, 165 mM KCl, 15 mM NaCl) and then snap cooling on ice for at least 10 min. The folded RNA was incubated with test compounds for 30 min at room temperature with the buffer adjusted to 1× Assay Buffer (20 mM HEPES, pH 7.5, 110 mM KCl, 10 mM NaCl, 2 mM MgCl_2_, 2 mM CaCl_2_, 5 mM dithiothreitol (DTT), 0.1% (w/v) BSA, and 0.05% (v/v) Tween-20) during compound addition. Following this incubation, r­(CUG)_12_ RNA (final concentration: 160 nM) was added to MBNL1-containing samples (MBNL1 final concentration: 120 nM), or vice versa, and the reaction was incubated for an additional 15 min at room temperature. To establish maximum TR-FRET (no small molecule control), samples were prepared with r­(CUG)_12_ and MBNL1 but without test compounds. To determine minimum TR-FRET, samples were prepared without MBNL1, replacing the volume of the protein solution added with 1× TR-FRET Assay Buffer. To detect RNA–MBNL1 complex formation, Streptavidin-XL665 (Revvity, catalog #610SAXLF) and Tb-Anti-His_6_ antibody (Revvity, catalog #61HISTLF) were added to final concentrations of 80 nM and 0.88 ng/μL. Samples were incubated for 30 min at room temperature before measuring TR-FRET.

TR-FRET was measured using a Molecular Devices SpectraMax M5 plate reader in time-resolved fluorescence mode, with a delay time of 200 μs and an integration time of 1500 μs. Fluorescence intensity was acquired at two emission wavelengths: 545 nm (for the Anti-His6-Terbium signal) and 665 nm (for the FRET signal to Streptavidin-XL665), both with an excitation wavelength of 345 nm and a cutoff filter of 420 nm. The TR-FRET ratio, calculated as the fluorescence at 545 nm divided by the fluorescence at 665 nm, was used to quantify the interaction between MBNL1 and r­(CUG)_12_. TR-FRET inhibition rate was calculated using eq 4:

4 $$Inhibition\;rate=\frac{TR‐FRET_{\mathrm{maximum}}-TR‐FRET_{sample}}{TR‐FRET_{\mathrm{maximum}}-TR‐FRET_{\mathrm{minimum}}}\times 100\%$$

The IC_50_ (half-maximal inhibitory concentration) is calculated by fitting the dose–response curve to a four-parameter logistic (4PL) equation, commonly used in inhibition assays:

5 $$Inhibition\;rate=\frac{100}{1+10^{\left(\log\;IC_{50}-concentration\right)\times Hill\;Slope}}$$

### Differential Scanning Fluorimetry

All experiments were performed using a QuantStudio 5, 384-well Block Real-Time PCR System (Applied Biosystems). Melting curve analyses were conducted using a temperature range from 20 to 95 °C, a ramp rate of 0.01 °C/s, and 75 acquisitions per °C. Melting temperatures (*T*_m_) were determined as the peak of the negative first derivative of the fluorescence curve. Each condition was measured in triplicate, and Δ*T*_m_ values were calculated relative to vehicle control. The FAM-r­(CUG)_10_-BHQ (FAM-CCGC­UGCU­GCUG­CUGC­UGCU­GCUG­CUGC­UGCU­GCGG-BHQ) oligonucleotide was purchased from Dharmacon (HPLC purified). Briefly, 500 nM FAM-r­(CUG)_10_-BHQ was folded by heating at 95 °C for 2 min in 20 mM NaH_2_PO_4_/Na_2_HPO_4_ buffer, pH 7.4 and then snap cooling on ice for at least 10 min. The folded RNA was incubated with test compounds at the indicated concentrations for 30 min at room temperature. In the melting curve method, fluorescence intensity was monitored using the FAM filter channel.

DSF experiments to assess compound–MBNL1 interactions were performed using SYPRO Orange Dye (Invitrogen, catalog #S6651). MBNL1 protein (10 μM) was pre-incubated with each compound at the indicated concentrations in 1× DSF buffer (20 mM HEPES, pH 7.5, 110 mM KCl, and 10 mM NaCl) for 30 min at room temperature. After incubation, SYPRO Orange Dye was added to a final concentration of 10× (supplied as a 500× stock; Invitrogen, catalog #S6651). Samples (10 μL) were transferred to a 384-well PCR plate, and fluorescence intensity as a function of temperature was monitored using the TAMRA filter channel.

### Cell Culture and Differentiation: DM1 and Wild Type Myotubes

DM1 patient-derived conditional MyoD-fibroblasts (*DMPK* bearing 1300 CUG repeats) and wild-type conditional MyoD-fibroblasts were generously provided by Denis Furling (Centre de Recherche en Myologie, UPMC/INSERM/CNRS, Institute Myologie, Paris, France).^72^ Cells were grown and differentiated at 37 °C in a 5% CO_2_ atmosphere. Growth medium consisted of 1× DMEM with 4.5 g/L glucose (without l-glutamine and sodium pyruvate; Corning, catalog #15-017-CV), supplemented with 15% (v/v) fetal bovine serum (FBS, Gibco, catalog #16-000-044), 1% (v/v) Glutagro (200 mM; Corning, catalog #25-015-CI), and 1% (v/v) Antibiotic-Antimycotic Solution (Corning, catalog #30-004-CI). Differentiation medium comprised 1× DMEM with 4.5 g/L glucose (without l-glutamine and sodium pyruvate; Corning, catalog #15-017-CV) with 1% (v/v) Antibiotic-Antimycotic Solution, 100 μg/mL human transferrin (Sigma, catalog #T8158-1G), 10 μg/mL insulin (10 mg/mL; Sigma-Aldrich, catalog #I0516-5ML), and 2 μg/mL doxycycline (freshly added; 1000× stock in DMSO) to induce differentiation. Both DM1 patient-derived fibroblasts and those derived from a healthy donor were used at passage numbers less than 20. Cells were tested for mycoplasma contaminations and determined to be mycoplasma-free before used in experiments.

### Analysis of MBNL1 Exon 5 (MBNL1-Regulated) and MAP4K4 Exon 22a (Nova-Regulated) Alternative Splicing

Rescue of disease-associated splicing defects by small molecules was performed as described in ref 72. In brief, DM1 patient-derived fibroblasts were seeded in 12-well plates and maintained in growth medium until they reached 100% confluency. The growth medium was removed and replaced with differentiation medium with or without compound, and the cells were incubated for 48 h. Following treatment, cells were lysed, and total RNA was extracted using the Zymo Quick RNA Miniprep Kit according to the manufacturer’s instructions, including the on-column DNase I digestion.

Approximately 200 ng of total RNA was reverse transcribed at 50 °C using either 100 units of SuperScript III reverse transcriptase (Life Technologies) or the qScript cDNA synthesis kit (20 μL total reaction volume, Quanta BioSciences) following the respective manufacturer’s protocol. Subsequently, 2 μL of the reverse transcription reaction was amplified using GoTaq DNA polymerase (Promega) per the manufacturer’s recommended protocol in a volume of 25 μL. RT-PCR was carried out for 30 cycles under the following conditions: 95 °C for 30 s, 58 °C for 30 s, 72 °C for 1 min, with a final extension at 72 °C for 5 min using primers listed in Table S2 for *MBNL1* exon 5 or *MAP4K4* exon 22a. The amplified PCR products were analyzed and quantified using a 5300 Fragment Analyzer System (Agilent Technologies) with the dsDNA 910 Reagent Kit (35–1500 bp, catalog #DNF910 K0500).

### Measuring MyoD and DMPK Transcript Abundance

*MyoD* transcript levels were measured by RT-qPCR as a biomarker for differentiation, while *DMPK* level were quantified to exclude compounds that affect transcription. For these analyses, the same cDNA samples generated from the splicing analysis of *MBNL1* exon 5 and *MAP4K4* exon 22a were used. For each biological replicate, a 35 μL master mix was prepared to accommodate three technical replicates (3 × 10 μL each) plus a small surplus for pipetting. This master mix contained 17.5 μL of 2× SYBR Green Master Mix (Applied Biosystems), 0.2 μM of each forward and reverse primer, 2 μL of cDNA template (from the reverse transcription reaction), and nuclease-free water to bring the total volume to 35 μL. QPCR amplification was completed under the following cycling conditions: 95 °C for 2 min, followed by 40 cycles of 95 °C for 15 s and 60 °C for 1 min. A melting curve analysis was performed to confirm the specificity of amplification. Relative expression levels of *MyoD* and *DMPK* were normalized to *GAPDH* as an internal control using the ΔΔCt method.^98^

### Imaging Nuclear Foci in DM1 Patient-Derived Myotubes

DM1 patient-derived conditional MyoD-fibroblasts and wild-type MyoD-fibroblasts were cultured and differentiated as described in “Cell Culture and Differentiation: DM1 and Wild Type Myotubes”. Compounds were added at their respective concentrations during differentiation and incubated for 48h before imaging. Following differentiation, DM1 myotubes were fixed, permeabilized, and incubated with Anti-MBNL1 primary antibody, followed by Alexa Fluor 488-conjugated secondary antibody, as described in “Validation of the NanoBRET Assay by Assessing smFISH/Foci”. For RNA foci imaging, single-molecule fluorescence *in situ* hybridization (smFISH) probes targeting *DMPK* CDS (Table S5) were hybridized to cells as described in “Validation of the NanoBRET Assay by Assessing smFISH/Foci”. Cells were mounted with 20 μL ProLong Gold Antifade Mountant with DNA Stain DAPI and imaged using LSM 980 Airyscan 2 Laser Scanning Confocal Microscope (Zeiss). To quantify nuclear foci, images were processed using FIJI. Intensity thresholds were set individually for FISH and MBNL1 channels to ensure no haze in the background and complete detection of all dots. RNA foci were defined as FISH-positive regions with a minimum area of 0.2 μm^2^. MBNL1-positive foci were identified as overlapping regions between the MBNL1 and RNA staining. The reduction in nuclear foci formation was compared between untreated and small molecule-treated DM1 myotubes.

### NMR Spectroscopy

NMR spectra were recorded on a 700 MHz Bruker Avance III spectrometer equipped with a cryogenic probe. RNAs (r­(GACC­UGCU­GGU­**GAAA**­ACC­UGC­UGG­UC) where the underlined Us indicate the internal loop nucleotides and bold indicate a GNRA hairpin) and (r­(GACC­AGCU­GGU­**GAAA**­ACCA­GCU­GGUC) where UU internal loops were replaced with AU base pairs) were purchased from Dharmacon, HPLC purified, deprotected and desalted. The RNA (50 μM) was dissolved in NMR Buffer (5 mM KH_2_PO_4_/K_2_HPO_4_, 0.25 mM EDTA, pH 6.0) or NMR Buffer supplemented with 50 mM NaCl and reannealed by heating to 95 °C for 3 min, and then slowly cooling the sample to room temperature before adding to Shigemi NMR tubes (Shigemi, Inc.).

1D ^1^H NMR spectra of exchangeable (imino) protons were acquired in 5% D_2_O and 95% H_2_O at 9 °C using 50 μM of RNA in the absence of compound. Compounds were then dissolved in D6-DMSO and added to the RNA sample to achieve a final concentration of 100 μM (1:2 RNA:compound). The chemical shifts were referenced to the residual solvent peaks of D6-DMSO, which served as the internal calibration standard. NMR spectra were processed in Topspin 4.0.6.

### Fluorescence Binding Assays

Binding assays employed the same RNA used in NMR studies (see “NMR Spectroscopy”). All binding experiments were performed in 1× TR-FRET Assay Buffer lacking Tween-20 on black 384-well nonbinding microplates (Greiner. catalog #781900) with a total volume of 10 μL per well. A 100 nM solution of A6 (the fluorescent probe) was prepared in the same buffer and increasing concentrations of the RNA were then titrated into the A6 solution. After a 20 min incubation at room temperature, fluorescence was measured by a Spectra Max M5 plate reader using an excitation wavelength of 320 nm and an emission wavelength of 480 nm. To confirm specificity, parallel measurements were performed with a fully paired RNA construct as a negative control, where no significant change in fluorescence was observed. The change in fluorescence signal as a function of RNA concentration was fitted to a standard ligand binding for one site saturation

6 $$F_{obs}=F_{0}+\frac{F_{\max}-F_{0}}{1+{\left(\frac{K_{d}}{\left[RNA\right]}\right)}^{n}}$$

where *F*_obs_ is the observed fluorescence at each RNA concentration, *F*_0_ and *F*_max_ are the fluorescence intensities of free and fully bound RNA, *K*_d_ is the dissociation constant, and *n* is the Hill coefficient.

## Supplementary Material



## Notes

Additional experimental data; materials including plasmid sequences and primer sequences used in this study; and compound characterization (PDF)
