# Supplementary material for: A Live-Cell NanoBRET Assay to Monitor RNA–Protein Interactions and Their Inhibition by Small Molecules
Source: ACS Cent Sci. 2025 Sep 25;11(11):2154–71. doi: 10.1021/acscentsci.5c00705 (PMC12670313; doi:10.1021/acscentsci.5c00705)
Supplement: Supplementary file 1 [file oc5c00705_si_001.pdf]

Supporting Information for:

A Live-Cell NanoBRET Assay to Monitor RNA–Protein Interactions and their Inhibition by Small Molecules

Jingsong Shan[1, 2], Amirhossein Taghavi[2], Elizabeth A. Caine[3], Ryuichi Sekioka[2], Veronika Rajchin[1, 2], James M. Burke[4], Monty Watkins[4], Jessica L. Childs-Disney[2], Matthew D. Disney[1, 2*]

[1]Department of Chemistry, The Scripps Research Institute, 130 Scripps Way, Jupiter, FL 33458 USA

[2]Department of Chemistry, The Herbert Wertheim UF Scripps Institute for Biomedical Innovation and Technology, 130 Scripps Way, Jupiter, FL 33458 USA

[3]Promega Corporation, 2800 Woods Hollow Road, Madison, WI 53711 USA

[4]Department of Molecular Medicine, The Herbert Wertheim UF Scripps Institute for Biomedical Innovation and Technology, 130 Scripps Way, Jupiter, FL 33458 USA

*Author to whom correspondence should be addressed mdisney@ufl.edu

Supplementary Tables & Figures

Table S1: Sequences of the plasmids used in this study that encode MBNL1 fusion proteins.

MBNL1-NanoLuc plasmid sequence (nucleotides that encode NanoLuc are underlined while nucleotides that encode **MBNL1** are in **bold**):

cctaccgcggatatctagatttgggcccaattcctgcaggcgagctctcggctcgagcggcgtcttcacactcgaagatttcgttgggga
ctggcgacagacagccggctacaacctggaccaagtccttgaacagggaggtgtgtccagtttgtttcagaatctcggggtgtccgta
actccgatccaaaggattgtcctgagcggtgaaaatggcctgaagatcgacatccatgtcatcatcccgtatgaaggtctgagcggcg
accaaatggccagatcgaaaaaatttttaaggtggtgtaccctgtggatgatcatcactttaaggtgatcctgcactatggcacactgg
taatcgacggggttacgccgaacatgatcgactatttcggacggccgtatgaaggcatcgccgtgttcgacggcaaaaagatcactgt
aacagggaccctgtggaacggcaacaaaattatcgacgagcgcctgatcaaccccgacggctccctgctgttccgagtaaccatca
acggagtgaccggctggcggctgtgcgaacgcattctggcgtaaggccgcgactctagggcatgcaagctgatccggctgctaaca
aagcccgaaaggaagctgagttggctgctgccaccgctgagcaataactagcataaccccttggggcggccgcttcgagcagacat
gataagatacattgatgagtttggacaaaccacaactagaatgcagtgaaaaaaatgctttatttgtgaaatttgtgatgctattgctttattt
gtaaccattataagctgcaataaacaagttaacaacaacaattgcattcattttatgtttcaggttcagggggagatgtgggaggtttttta
agcaagtaaaacctctacaaatgtggtaaaatcgaattttaacaaaatattaacgcttacaatttcctgatgcggtattttctccttacgcat
ctgtgcggtatttcacaccgcatacgcggatctgcgcagcaccatggcctgaaataacctctgaaagaggaacttggttaggtaccttc
tgaggcggaaagaaccagctgtggaatgtgtgtcagttagggtgtggaaagtccccaggctccccagcaggcagaagtatgcaaa
gcatgcatctcaattagtcagcaaccaggtgtggaaagtccccaggctccccagcaggcagaagtatgcaaagcatgcatctcaatt
agtcagcaaccatagtcccgcccctaactccgcccatcccgcccctaactccgcccagttccgcccattctccgccccatggctgact
aattttttttatttatgcagaggccgaggccgcctcggcctctgagctattccagaagtagtgaggaggcttttttggaggcctaggcttttg
caaaaagctcgattcttctgacactagcgccaccatgaagaagcccgaactcaccgctaccagcgttgaaaaatttctcatcgagaa
gttcgacagtgtgagcgacctgatgcagttgtcggagggcgaagagagccgagccttcagcttcgatgtcggcggacgcggctatgt
actgcgggtgaatagctgcgctgatggcttctacaaagaccgctacgtgtaccgccacttcgccagcgctgcactacccatccccgaa
gtgttggacatcggcgagttcagcgagagcctgacatactgcatcagtagacgcgcccaaggcgttactctccaagacctccccgaa
acagagctgcctgctgtgttacagcctgtcgccgaagctatggatgctattgccgccgccgacctcagtcaaaccagcggcttcggcc
cattcgggccccaaggcatcggccagtacacaacctggcgggatttcatttgcgccattgctgatccccatgtctaccactggcagacc
gtgatggacgacaccgtgtccgccagcgtagctcaagccctggacgaactgatgctgtgggccgaagactgtcccgaggtgcgcca
cctcgtccatgccgacttcggcagcaacaacgtcctgaccgacaacggccgcatcaccgccgtaatcgactggtccgaagctatgtt
cggggacagtcagtacgaggtggccaacatcttcttctggcggccctggctggcttgcatggagcagcagactcgctacttcgagcgc
cggcatcccgagctggccggcagccctcgtctgcgagcctacatgctgcgcatcggcctggatcagctctaccagagcctcgtggac
ggcaacttcgacgatgctgcctgggctcaaggccgctgcgatgccatcgtccgcagcggggccggcaccgtcggtcgcacacaaat
cgctcgccggagcgcagccgtatggaccgacggctgcgtcgaggtgctggccgacagcggcaaccgccggcccagtacacgac
cgcgcgctaaggaggtaggtcgagtttagactctagccatcacgatggccgcaataaaatatctttattttcattacatctgtgtgttggtttt
ttgtgtgaatcgatagcgataaggatcctctttgcgcttgcgttttcccttgtccagatagcccagtagctgacattcatccggggtcagca
ccgtttctgcggactggctttctacgtaatggtttcttagacgtcaggtggcacttttcggggaaatgtgcgcggaacccctatttgtttattttt
ctaaatacattcaaatatgtatccgctcatgagacaataaccctgataaatgcttcaataatattgaaaaaggaagagtatgagtattca
acatttccgtgtcgcccttattcccttttttgcggcattttgccttcctgtttttgctcacccagaaacgctggtgaaagtaaaagatgctgaag
atcagttgggtgcacgagtgggttacatcgaactggatctcaacagcggtaagatccttgagagttttcgccccgaagaacgttttccaa
tgatgagcacttttaaagttctgctatgtggcgcggtattatcccgtattgacgccgggcaagagcaactcggtcgccgcatacactatt
ctcagaatgacttggttgagtactcaccagtcacagaaaagcatcttacggatggcatgacagtaagagaattatgcagtgctgccat
aaccatgagtgataacactgcggccaacttacttctgacaactatcggaggaccgaaggagctaaccgcttttttgcacaacatgggg
gatcatgtaactcgccttgatcgttgggaaccggagctgaatgaagccataccaaacgacgagcgtgacaccacgatgcctgtagc
aatggcaacaacgttgcgcaaactattaactggcgaactacttactctagcttcccggcaacaattaatagactggatggaggcggat
aaagttgcaggaccacttctgcgctcggcccttccggctggctggtttattgctgataaatctggagccggtgagcgtgggtctcgcggt
atcattgcagcactggggccagatggtaagccctcccgtatcgtagttatctacacgacggggagtcaggcaactatggatgaacga
aatagacagatcgctgagataggtgcctcactgattaagcattggtaattcgaaatgaccgaccaagcgacgcccaaccggtatcag
ctcactcaaaggcggtaatacggttatccacagaatcaggggataacgcaggaaagaacatgtgagcaaaaggccagcaaaag

gccaggaaccgtaaaaaggccgcgttgctggcgtttttccataggctccgcccccctgacgagcatcacaaaaatcgacgctcaagt
cagaggtggcgaaacccgacaggactataaagataccaggcgtttccccctggaagctccctcgtgcgctctcctgttccgaccctgc
cgcttaccggatacctgtccgcctttctcccttcgggaagcgtggcgctttctcatagctcacgctgtaggtatctcagttcggtgtaggtcg
ttcgctccaagctgggctgtgtgcacgaaccccccgttcagcccgaccgctgcgccttatccggtaactatcgtcttgagtccaacccg
gtaagacacgacttatcgccactggcagcagccactggtaacaggattagcagagcgaggtatgtaggcggtgctacagagttcttg
aagtggtggcctaactacggctacactagaaggacagtatttggtatctgcgctctgctgaagccagttaccttcggaaaaagagttgg
tagctcttgatccggcaaacaaaccaccgctggtagcggtggtttttttgtttgcaagcagcagattacgcgcagaaaaaaaggatttc
aagaagatcctttgatcttttctacggggtctgacgctcagtggaacgaaaactcacgttaagggattttggtcatgagattatcaaaaag
gatcttcacctagatccttttatagtccggaaatacaggaacgcacgctggatggcccttcgctgggatggtgaaaccatgaaaaatgg
cagcttcagtggattaagtgggggtaatgtggcctgtaccctctggttgcataggtattcatacggttaaaatttatcaggcgcgattgcgg
cagtttttcgggtggtttgttgccatttttacctgtctgctgccgtgatcgcgctgaacgcgttttagcggtgcgtacaattaagggattatggt
aaatccacttactgtctgccctcgtagccatcgagataaaccgcagtactccggccacgatgcgtccggcgtagaggatcgagatctt
caatattggccattagccatattattcattggttatatagcataaatcaatattggctattggccattgcatacgttgtatctatatcataatatgt
acatttatattggctcatgtccaatatgaccgccatgttggcattgattattgactagttattaatagtaatcaattacggggtcattagttcata
gcccatatatggagttccgcgttacataacttacggtaaatggcccgcctggctgaccgcccaacgacccccgcccattgacgtcaat
aatgacgtatgttcccatagtaacgccaatagggactttccattgacgtcaatgggtggagtatttacggtaaactgcccacttggcagt
acatcaagtgtatcatatgccaagtccgccccctattgacgtcaatgacggtaaatggcccgcctggcattatgcccagtacatgacctt
acgggactttcctacttggcagtacatctacgtattagtcatcgctattaccatggtgatgcggttttggcagtacaccaatgggcgtggat
agcggtttgactcacggggatttccaagtctccaccccattgacgtcaatgggagtttgttttggcaccaaaatcaacgggactttccaa
aatgtcgtaataaccccgccccgttgacgcaaatgggcggtaggcgtgtacggtgggaggtctatataagcagagctggtttagtgaa
ccgtcagatcactagaagctttattgcggtagtttatcacagttaaattgctaacgcagtcagtgcttctgacacaacagtctcgaacttaa
gctgcagaagttggtcgtgaggcactgggcaggtaagtatcaaggttacaagacaggtttaaggagaccaatagaaactgggcttgt
cgagacagagaagactcttgcgtttctgataggcacctattggtcttactgacatccactttgcctttctctccacaggtgtccactcccagt
tcaattacagctcttaaggctagagtattaatacgactcactatagggctagcaaagcgatcgcttccg**atggctgtttccgtcacacc
aattcgggacacaaaatggctaacactggaagtatgtagagagttccagaggggacttgctcacggccagacacggaa
tgtaaatttgcacatccttcgaaaagctgccaagttgaaaatggacgagtaatcgcctgctttgattcattgaaaggccgttgc
tccagggagaactgcaaatatcttcatccacccccacatttaaaaacgcagttggagataaatggacgcaat**aacttgattca
gcagaagaacatggccatgttggcccagcaaatgcaactagccaatgccatgatgcctggtgccccattacaacccgtgccaatgttt
tcagttgcaccaagcttagccaccaatgcatcagcagccgcctttaatccctatctgggacctgtttctccaagcctggtcccggcagag
atcttgccgactgcaccaatgttggttacagggaatccgggtgtccctgtacctgcagctgctgcagctgctgcacagaaattaatgcg
aacagacagacttgaggtatgtcgagagtaccaacgtggcaattgcaaccgaggagaaaatgattgtcggtttgctcatcctgctgac
agcacaatgattgacaccaatgacaacacagtcactgtgtgtatggattacatcaaagggagatgctctcgggaaaagtgcaaatac
tttcatccccctgcacatttgcaagccaagatcaaggctgcccaatacca

MBNL1-HaloTag plasmid sequence (nucleotides that encode HaloTag are underlined while nucleotides that encode **MBNL1** are in **bold**):

cctaccgcggatatctagatttgggcccaattcctgcaggcgagctctcgagccaaccactgaggatctgtactttcagagcgataacg
atggatccgaaatcggtactggctttccattcgacccccattatgtggaagtcctgggcgagcgcatgcactacgtcgatgttggtccgc
gcgatggcacccctgtgctgttcctgcacggtaacccgacctcctcctacgtgtggcgcaacatcatcccgcatgttgcaccgacccat
cgctgcattgctccagacctgatcggtatgggcaaatccgacaaaccagacctgggttatttcttcgacgaccacgtccgcttcatggat
gccttcatcgaagccctgggtctggaagaggtcgtcctggtcattcacgactggggctccgctctgggtttccactgggccaagcgcaa
tccagagcgcgtcaaaggtattgcatttatggagttcatccgccctatcccgacctgggacgaatggccagaatttgcccgcgagacct
tccaggccttccgcaccaccgacgtcggccgcaagctgatcatcgatcagaacgtttttatcgagggtacgctgccgatgggtgtcgtc
cgcccgctgactgaagtcgagatggaccattaccgcgagccgttcctgaatcctgttgaccgcgagccactgtggcgcttcccaaacg
agctgccaatcgccggtgagccagcgaacatcgtcgcgctggtcgaagaatacatggactggctgcaccagtcccctgtcccgaag
ctgctgttctggggcaccccaggcgttctgatcccaccggccgaagccgctcgcctggccaaaagcctgcctaactgcaaggctgtg
gacatcggcccgggtctgaatctgctgcaagaagacaacccggacctgatcggcagcgagatcgcgcgctggctgtctactctgga
gatttccggttaatagaattggcatgcaagctgatccggctgctaacaaagcccgaaaggaagctgagttggctgctgccaccgctga
gcaataactagcataaccccttggggcggccgcttcgagcagacatgataagatacattgatgagtttggacaaaccacaactagaa

tgcagtgaaaaaaatgctttatttgtgaaatttgtgatgctattgctttatttgtaaccattataagctgcaataaacaagttaacaacaaca
attgcattcattttatgtttcaggttcagggggagatgtgggaggttttttaagcaagtaaaacctctacaaatgtggtaaaatcgaattta
acaaaatattaacgcttacaatttcctgatgcggtattttctccttacgcatctgtgcggtatttcacaccgcatacgcggatctgcgcagca
ccatggcctgaaataacctctgaaagaggaacttggttaggtaccttctgaggcggaaagaaccagctgtggaatgtgtgtcagttag
ggtgtggaaagtccccaggctccccagcaggcagaagtatgcaaagcatgcatctcaattagtcagcaaccaggtgtggaaagtcc
ccaggctccccagcaggcagaagtatgcaaagcatgcatctcaattagtcagcaaccatagtcccgcccctaactccgcccatccc
gcccctaactccgcccagttccgcccattctccgccccatggctgactaattttttttatttatgcagaggccgaggccgcctcggcctctg
agctattccagaagtagtgaggaggcttttttggaggcctaggcttttgcaaaaagcttgattcttctgacacaacagtctcgaacttaag
gctagagccaccatgattgaacaagatggattgcacgcaggttctccggccgcttgggtggagaggctattcggctatgactgggcac
aacagacaatcggctgctctgatgccgccgtgttccggctgtcagcgcaggggcgcccggttctttttgtcaagaccgacctgtccggt
gccctgaatgaactgcaggacgaggcagcgcggctatcgtggctggccacgacgggcgttccttgcgcagctgtgctcgacgttgtc
actgaagcgggaagggactggctgctattgggcgaagtgccggggcaggatctcctgtcatctcaccttgctcctgccgagaaagtat
ccatcatggctgatgcaatgcggcggctgcatacgcttgatccggctacctgcccattcgaccaccaagcgaaacatcgcatcgagc
gagcacgtactcggatggaagccggtcttgtcgatcaggatgatctggacgaagagcatcaggggctcgcgccagccgaactgttc
gccaggctcaaggcgcgcatgcccgacggcgaggatctcgtcgtgacccatggcgatgcctgcttgccgaatatcatggtggaaaat
ggccgcttttctggattcatcgactgtggccggctgggtgtggcggaccgctatcaggacatagcgttggctacccgtgatattgctgaa
gagcttggcggcgaatgggctgaccgcttcctcgtgctttacggtatcgccgctcccgattcgcagcgcatcgccttctatcgccttcttga
cgagttcttctgagcgggactctggggttcgaaatgaccgaccaagcgacgcccaacctgccatcacgatggccgcaataaaatatc
tttattttcattacatctgtgtgttggttttttgtgtgaatcgatagcgataaggatcctctttgcgcttgcgttttcccttgtccagatagcccagta
gctgacattcatccggggtcagcaccgtttctgcggactggctttctacgtaatggtttcttagacgtcaggtggcacttttcggggaaatgt
gcgcggaacccctatttgtttatttttctaaatacattcaaatatgtatccgctcatgagacaataaccctgataaatgcttcaataatattga
aaaaggaagagtatgagtattcaacatttccgtgtcgcccttattcccttttttgcggcattttgccttcctgtttttgctcacccagaaacgct
ggtgaaagtaaaagatgctgaagatcagttgggtgcacgagtgggttacatcgaactggatctcaacagcggtaagatccttgagag
ttttcgccccgaagaacgttttccaatgatgagcacttttaaagttctgctatgtggcgcggtattatcccgtattgacgccgggcaagag
caactcggtcgccgcatacactattctcagaatgacttggttgagtactcaccagtcacagaaaagcatcttacggatggcatgacagt
aagagaattatgcagtgctgccataaccatgagtgataacactgcggccaacttacttctgacaactatcggaggaccgaaggagct
aaccgcttttttgcacaacatgggggatcatgtaactcgccttgatcgttgggaaccggagctgaatgaagccataccaaacgacgag
cgtgacaccacgatgcctgtagcaatggcaacaacgttgcgcaaactattaactggcgaactacttactctagcttcccggcaacaatt
aatagactggatggaggcggataaagttgcaggaccacttctgcgctcggcccttccggctggctggtttattgctgataaatctggagc
cggtgagcgtgggtctcgcggtatcattgcagcactggggccagatggtaagccctcccgtatcgtagttatctacacgacggggagt
caggcaactatggatgaacgaaatagacagatcgctgagataggtgcctcactgattaagcattggtaattcgaaatgaccgaccaa
gcgacgcccaaccggtatcagctcactcaaaggcggtaatacggttatccacagaatcaggggataacgcaggaaagaacatgtg
agcaaaaggccagcaaaaggccaggaaccgtaaaaaggccgcgttgctggcgtttttccataggctccgcccccctgacgagcat
cacaaaaatcgacgctcaagtcagaggtggcgaaacccgacaggactataaagataccaggcgtttccccctggaagctccctcgt
gcgctctcctgttccgaccctgccgcttaccggatacctgtccgcctttctcccttcgggaagcgtggcgctttctcatagctcacgctgtag
gtatctcagttcggtgtaggtcgttcgctccaagctgggctgtgtgcacgaaccccccgttcagcccgaccgctgcgccttatccggtaa
ctatcgtcttgagtccaacccggtaagacacgacttatcgccactggcagcagccactggtaacaggattagcagagcgaggtatgt
aggcggtgctacagagttcttgaagtggtggcctaactacggctacactagaaggacagtatttggtatctgcgctctgctgaagccag
ttaccttcggaaaaagagttggtagctcttgatccggcaaacaaaccaccgctggtagcggtggtttttttgtttgcaagcagcagattac
gcgcagaaaaaaaggatttcaagaagatcctttgatcttttctacggggtctgacgctcagtggaacgaaaactcacgttaagggatttt
ggtcatgagattatcaaaaaggatcttcacctagatccttttatagtccggaaatacaggaacgcacgctggatggcccttcgctgggat
ggtgaaaccatgaaaaatggcagcttcagtggattaagtgggggtaatgtggcctgtaccctctggttgcataggtattcatacggttaa
aatttatcaggcgcgattgcggcagtttttcgggtggtttgttgccatttttacctgtctgctgccgtgatcgcgctgaacgcgttttagcggtg
cgtacaattaagggattatggtaaatccacttactgtctgccctcgtagccatcgagataaaccgcagtactccggccacgatgcgtcc
ggcgtagaggatcgagatcttcaatattggccattagccatattattcattggttatatagcataaatcaatattggctattggccattgcata
cgttgtatctatatcataatatgtacatttatattggctcatgtccaatatgaccgccatgttggcattgattattgactagttattaatagtaatc
aattacggggtcattagttcatagcccatatatggagttccgcgttacataacttacggtaaatggcccgcctggctgaccgcccaacg
acccccgcccattgacgtcaataatgacgtatgttcccatagtaacgccaatagggactttccattgacgtcaatgggtggagtatttac
ggtaaactgcccacttggcagtacatcaagtgtatcatatgccaagtccgccccctattgacgtcaatgacggtaaatggcccgcctgg
cattatgcccagtacatgaccttacgggactttcctacttggcagtacatctacgtattagtcatcgctattaccatggtgatgcggttttggc
agtacaccaatgggcgtggatagcggtttgactcacggggatttccaagtctccaccccattgacgtcaatgggagtttgttttggcacc

aaaatcaacgggactttccaaaatgtcgtaataaccccgccccgttgacgcaaatgggcggtaggcgtgtacggtgggaggtctatat
aagcagagctggtttagtgaaccgtcagatcactagaagctttattgcggtagtttatcacagttaaattgctaacgcagtcagtgcttctg
acacaacagtctcgaacttaagctgcagaagttggtcgtgaggcactgggcaggtaagtatcaaggttacaagacaggtttaaggag
accaatagaaactgggcttgtcgagacagagaagactcttgcgtttctgataggcacctattggtcttactgacatccactttgcctttctct
ccacaggtgtccactcccagttcaattacagctcttaaggctagagtattaatacgactcactatagggctagcaaagcgatcgcttccg
atggctgtttccgtcacaccaattcgggacacaaaatggctaacactggaagtatgtagagagttccagaggggacttgc
tcacggccagacacggaatgtaaatttgcacatccttcgaaaagctgccaagttgaaaatggacgagtaatcgcctgctttg
attcattgaaaggccgttgctccagggagaactgcaaatatcttcatcccccccacatttaaaaacgcagttggagataaat
ggacgcaataacttgattcagcagaagaacatggccatgttggcccagcaaatgcaactagccaatgccatgatgcctggtgcccc
attacaacccgtgccaatgttttcagttgcaccaagcttagccaccaatgcatcagcagccgcctttaatccctatctgggacctgtttctc
caagcctggtcccggcagagatcttgccgactgcaccaatgttggttacagggaatccgggtgtccctgtacctgcagctgctgcagct
gctgcacagaaattaatgcgaacagacagacttgaggtatgtcgagagtaccaacgtggcaattgcaaccgaggagaaaatgattg
tcggtttgctcatcctgctgacagcacaatgattgacaccaatgacaacacagtcactgtgtgtatggattacatcaaagggagatgctc
tcgggaaaagtgcaaatactttcatccccctgcacatttgcaagccaagatcaaggctgcccaatacca

NanoLuc **- MBNL1** plasmid sequence (nucleotides that encode NanoLuc are underlined while nucleotides that encode **MBNL1** are in **bold**):

ag**atggctgtttccgtcacaccaattcgggacacaaaatggctaacactggaagtatgtagagagttccagaggggactt**
gctcacggccagacacggaatgtaaatttgcacatccttcgaaaagctgccaagttgaaaatggacgagtaatcgcctgct
ttgattcattgaaaggccgttgctccagggagaactgcaaatatcttcatcccccccacatttaaaaacgcagttggagata
aatggacgcaataacttgattcagcagaagaacatggccatgttggcccagcaaatgcaactagccaatgccatgatgcctggtgc
cccattacaacccgtgccaatgttttcagttgcaccaagcttagccaccaatgcatcagcagccgcctttaatccctatctgggacctgttt
ctccaagcctggtcccggcagagatcttgccgactgcaccaatgttggttacagggaatccgggtgtccctgtacctgcagctgctgca
gctgctgcacagaaattaatgcgaacagacagacttgaggtatgtcgagagtaccaacgtggcaattgcaaccgaggagaaaatg
attgtcggtttgctcatcctgctgacagcacaatgattgacaccaatgacaacacagtcactgtgtgtatggattacatcaaagggagat
gctctcgggaaaagtgcaaatactttcatccccctgcacatttgcaagccaagatcaaggctgcccaataccacagagctcaaccgc
ggatatctagatttgggcccaattcctgcaggattttgcggccgcttgctgagttggctgctgccaccgctgagcaataactagcataac
cccttggccgcttcgagcagacatgataagatacattgatgagtttggacaaaccacaactagaatgcagtgaaaaaaatgctttattt
gtgaaatttgtgatgctattgctttatttgtaaccattataagctgcaataaacaagttaacaacaacaattgcattcattttatgtttcaggttc
agggggagatgtgggaggttttttaagcaagtaaaacctctacaaatgtggtaaaatcgaattttaacaaaatattaacgcttacaattt
cctgatgcggtattttctccttacgcatctgtgcggtatttcacaccgcatacgcggatctgcgcagcaccatggcctgaaataacctctg
aaagaggaacttggttaggtaccttctgaggcggaaagaaccagctgtggaatgtgtgtcagttagggtgtggaaagtccccaggctc
cccagcaggcagaagtatgcaaagcatgcatctcaattagtcagcaaccaggtgtggaaagtccccaggctccccagcaggcaga
agtatgcaaagcatgcatctcaattagtcagcaaccatagtcccgcccctaactccgcccatcccgcccctaactccgcccagttccg
cccattctccgccccatggctgactaattttttttatttatgcagaggccgaggccgcctcggcctctgagctattccagaagtagtgagga
ggcttttttggaggcctaggcttttgcaaaaagctcgattcttctgacactagcgccaccatgaagaagcccgaactcaccgctaccag
cgttgaaaaatttctcatcgagaagttcgacagtgtgagcgacctgatgcagttgtcggagggcgaagagagccgagccttcagcttc
gatgtcggcggacgcggctatgtactgcgggtgaatagctgcgctgatggcttctacaaagaccgctacgtgtaccgccacttcgcca
gcgctgcactacccatccccgaagtgttggacatcggcgagttcagcgagagcctgacatactgcatcagtagacgcgcccaaggc
gttactctccaagacctccccgaaacagagctgcctgctgtgttacagcctgtcgccgaagctatggatgctattgccgccgccgacct
cagtcaaaccagcggcttcggcccattcgggccccaaggcatcggccagtacacaacctggcgggatttcatttgcgccattgctgat
ccccatgtctaccactggcagaccgtgatggacgacaccgtgtccgccagcgtagctcaagccctggacgaactgatgctgtgggcc
gaagactgtcccgaggtgcgccacctcgtccatgccgacttcggcagcaacaacgtcctgaccgacaacggccgcatcaccgccg
taatcgactggtccgaagctatgttcggggacagtcagtacgaggtggccaacatcttcttctggcggccctggctggcttgcatggag
cagcagactcgctacttcgagcgccggcatcccgagctggccggcagccctcgtctgcgagcctacatgctgcgcatcggcctggat
cagctctaccagagcctcgtggacggcaacttcgacgatgctgcctgggctcaaggccgctgcgatgccatcgtccgcagcggggc
cggcaccgtcggtcgcacacaaatcgctcgccggagcgcagccgtatggaccgacggctgcgtcgaggtgctggccgacagcgg
caaccgccggcccagtacacgaccgcgcgctaaggaggtaggtcgagtttagactctagccatcacgatggccgcaataaaatatc
tttattttcattacatctgtgtgttggtttttgtgtgaatcgatagcgataaggatcctctttgcgcttgcgttttcccttgtccagatagcccagta

gctgacattcatccggggtcagcaccgtttctgcggactggctttctacgtaatggtttcttagacgtcaggtggcacttttcggggaaatgt
gcgcggaacccctatttgtttatttttctaaatacattcaaatatgtatccgctcatgagacaataaccctgataaatgcttcaataatattga
aaaaggaagagtatgagtattcaacatttccgtgtcgcccttattcccttttttgcggcattttgccttcctgtttttgctcacccagaaacgct
ggtgaaagtaaaagatgctgaagatcagttgggtgcacgagtgggttacatcgaactggatctcaacagcggtaagatccttgagag
ttttcgccccgaagaacgttttccaatgatgagcacttttaaagttctgctatgtggcgcggtattatcccgtattgacgccgggcaagag
caactcggtcgccgcatacactattctcagaatgacttggttgagtactcaccagtcacagaaaagcatcttacggatggcatgacagt
aagagaattatgcagtgctgccataaccatgagtgataacactgcggccaacttacttctgacaactatcggaggaccgaaggagct
aaccgcttttttgcacaacatgggggatcatgtaactcgccttgatcgttgggaaccggagctgaatgaagccataccaaacgacgag
cgtgacaccacgatgcctgtagcaatggcaacaacgttgcgcaaactattaactggcgaactacttactctagcttcccggcaacaatt
aatagactggatggaggcggataaagttgcaggaccacttctgcgctcggcccttccggctggctggtttattgctgataaatctggagc
cggtgagcgtgggtctcgcggtatcattgcagcactggggccagatggtaagccctcccgtatcgtagttatctacacgacggggagt
caggcaactatggatgaacgaaatagacagatcgctgagataggtgcctcactgattaagcattggtaattcgaaatgaccgaccaa
gcgacgcccaaccggtatcagctcactcaaaggcggtaatacggttatccacagaatcaggggataacgcaggaaagaacatgtg
agcaaaaggccagcaaaaggccaggaaccgtaaaaaggccgcgttgctggcgtttttccataggctccgcccccctgacgagcat
cacaaaaatcgacgctcaagtcagaggtggcgaaacccgacaggactataaagataccaggcgtttccccctggaagctccctcgt
gcgctctcctgttccgaccctgccgcttaccggatacctgtccgcctttctcccttcgggaagcgtggcgctttctcatagctcacgctgtag
gtatctcagttcggtgtaggtcgttcgctccaagctgggctgtgtgcacgaaccccccgttcagcccgaccgctgcgccttatccggtaa
ctatcgtcttgagtccaacccggtaagacacgacttatcgccactggcagcagccactggtaacaggattagcagagcgaggtatgt
aggcggtgctacagagttcttgaagtggtggcctaactacggctacactagaaggacagtatttggtatctgcgctctgctgaagccag
ttaccttcggaaaaagagttggtagctcttgatccggcaaacaaaccaccgctggtagcggtggtttttttgtttgcaagcagcagattac
gcgcagaaaaaaaggatttcaagaagatcctttgatcttttctacggggtctgacgctcagtggaacgaaaactcacgttaagggatttt
ggtcatgagattatcaaaaaggatcttcacctagatccttttatagtccggaaatacaggaacgcacgctggatggcccttcgctgggat
ggtgaaaccatgaaaaatggcagcttcagtggattaagtgggggtaatgtggcctgtaccctctggttgcataggtattcatacggttaa
aatttatcaggcgcgattgcggcagtttttcgggtggtttgttgccatttttacctgtctgctgccgtgatcgcgctgaacgcgttttagcggtg
cgtacaattaagggattatggtaaatccacttactgtctgccctcgtagccatcgagataaaccgcagtactccggccacgatgcgtcc
ggcgtagaggatcgagatcttcaatattggccattagccatattattcattggttatatagcataaatcaatattggctattggccattgcata
cgttgtatctatatcataatatgtacatttatattggctcatgtccaatatgaccgccatgttggcattgattattgactagttattaatagtaatc
aattacggggtcattagttcatagcccatatatggagttccgcgttacataacttacggtaaatggcccgcctggctgaccgcccaacg
acccccgcccattgacgtcaataatgacgtatgttcccatagtaacgccaatagggactttccattgacgtcaatgggtggagtatttac
ggtaaactgcccacttggcagtacatcaagtgtatcatatgccaagtccgccccctattgacgtcaatgacggtaaatggcccgcctgg
cattatgcccagtacatgaccttacgggactttcctacttggcagtacatctacgtattagtcatcgctattaccatggtgatgcggttttggc
agtacaccaatgggcgtggatagcggtttgactcacggggatttccaagtctccaccccattgacgtcaatgggagtttgttttggcacc
aaaatcaacgggactttccaaaatgtcgtaataaccccgccccgttgacgcaaatgggcggtaggcgtgtacggtgggaggtctatat
aagcagagctggtttagtgaaccgtcagatcactagaagctttattgcggtagtttatcacagttaaattgctaacgcagtcagtgcttctg
acacaacagtctcgaacttaagctgcagaagttggtcgtgaggcactgggcaggtaagtatcaaggttacaagacaggtttaaggag
accaatagaaactgggcttgtcgagacagagaagactcttgcgtttctgataggcacctattggtcttactgacatccactttgcctttctct
ccacaggtgtccactcccagttcaattacagctcttaaggctagagtattaatacgactcactatagggctagcgctcaccatggtcttca
cactcgaagatttcgttggggactggcgacagacagccggctacaacctggaccaagtccttgaacagggaggtgtgtccagtttgttt
cagaatctcggggtgtccgtaactccgatccaaaggattgtcctgagcggtgaaaatgggctgaagatcgacatccatgtcatcatcc
cgtatgaaggtctgagcggcgaccaaatgggccagatcgaaaaaatttttaaggtggtgtaccctgtggatgatcatcactttaaggtg
atcctgcactatggcacactggtaatcgacggggttacgccgaacatgatcgactatttcggacggccgtatgaaggcatcgccgtgtt
cgacggcaaaaagatcactgtaacagggaccctgtggaacggcaacaaaattatcgacgagcgcctgatcaaccccgacggctc
cctgctgttccgagtaaccatcaacggagtgaccggctggcggctgtgcgaacgcattctggcgggctcgagcggcgcgatcgcttc
cg

HaoTag **- MBNL1** plasmid sequence (nucleotides that encode HaloTag are underlined while nucleotides that encode **MBNL1** are in **bold**):

cagagctcaaccgcggatatctagatttgggcccaattcctgcaggattttgcggccgcttgctgagttggctgctgccaccgctgagca
ataactagcataaccccttggccgcttcgagcagacatgataagatacattgatgagtttggacaaaccacaactagaatgcagtga

aaaaaatgctttatttgtgaaatttgtgatgctattgctttatttgtaaccattataagctgcaataaacaagttaacaacaacaattgcattc
attttatgtttcaggttcagggggagatgtgggaggttttttaagcaagtaaaacctctacaaatgtggtaaaatcgaattttaacaaaat
attaacgcttacaatttcctgatgcggtattttctccttacgcatctgtgcggtatttcacaccgcatacgcggatctgcgcagcaccatggc
ctgaaataacctctgaaagaggaacttggttaggtaccttctgaggcggaaagaaccagctgtggaatgtgtgtcagttagggtgtgg
aaagtccccaggctccccagcaggcagaagtatgcaaagcatgcatctcaattagtcagcaaccaggtgtggaaagtccccaggct
ccccagcaggcagaagtatgcaaagcatgcatctcaattagtcagcaaccatagtcccgcccctaactccgcccatcccgcccctaa
ctccgcccagttccgcccattctccgccccatggctgactaattttttttatttatgcagaggccgaggccgcctcggcctctgagctattcc
agaagtagtgaggaggcttttttggaggcctaggcttttgcaaaaagcttgattcttctgacacaacagtctcgaacttaaggctagagc
caccatgattgaacaagatggattgcacgcaggttctccggccgcttgggtggagaggctattcggctatgactgggcacaacagac
aatcggctgctctgatgccgccgtgttccggctgtcagcgcaggggcgcccggttctttttgtcaagaccgacctgtccggtgccctgaa
tgaactgcaggacgaggcagcgcggctatcgtggctggccacgacgggcgttccttgcgcagctgtgctcgacgttgtcactgaagc
gggaagggactggctgctattgggcgaagtgccggggcaggatctcctgtcatctcaccttgctcctgccgagaaagtatccatcatg
gctgatgcaatgcggcggctgcatacgcttgatccggctacctgcccattcgaccaccaagcgaaacatcgcatcgagcgagcacg
tactcggatggaagccggtcttgtcgatcaggatgatctggacgaagagcatcaggggctcgcgccagccgaactgttcgccaggct
caaggcgcgcatgcccgacggcgaggatctcgtcgtgacccatggcgatgcctgcttgccgaatatcatggtggaaaatggccgcttt
tctggattcatcgactgtggccggctgggtgtggcggaccgctatcaggacatagcgttggctacccgtgatattgctgaagagcttggc
ggcgaatgggctgaccgcttcctcgtgctttacggtatcgccgctcccgattcgcagcgcatcgccttctatcgccttcttgacgagttcttc
tgagcgggactctggggttcgaaatgaccgaccaagcgacgcccaacctgccatcacgatggccgcaataaaatatctttattttcatt
acatctgtgtgttggttttttgtgtgaatcgatagcgataaggatcctctttgcgcttgcgttttcccttgtccagatagcccagtagctgacatt
catccggggtcagcaccgtttctgcggactggctttctacgtaatggtttcttagacgtcaggtggcacttttcggggaaatgtgcgcgga
acccctatttgtttatttttctaaatacattcaaatatgtatccgctcatgagacaataaccctgataaatgcttcaataatattgaaaaagga
agagtatgagtattcaacatttccgtgtcgcccttattcccttttttgcggcattttgccttcctgtttttgctcacccagaaacgctggtgaaag
taaaagatgctgaagatcagttgggtgcacgagtgggttacatcgaactggatctcaacagcggtaagatccttgagagttttcgcccc
gaagaacgttttccaatgatgagcacttttaaagttctgctatgtggcgcggtattatcccgtattgacgccgggcaagagcaactcggt
cgccgcatacactattctcagaatgacttggttgagtactcaccagtcacagaaaagcatcttacggatggcatgacagtaagagaatt
atgcagtgctgccataaccatgagtgataacactgcggccaacttacttctgacaactatcggaggaccgaaggagctaaccgctttt
tgcacaacatgggggatcatgtaactcgccttgatcgttgggaaccggagctgaatgaagccataccaaacgacgagcgtgacacc
acgatgcctgtagcaatggcaacaacgttgcgcaaactattaactggcgaactacttactctagcttcccggcaacaattaatagactg
gatggaggcggataaagttgcaggaccacttctgcgctcggcccttccggctggctggtttattgctgataaatctggagccggtgagc
gtgggtctcgcggtatcattgcagcactggggccagatggtaagccctcccgtatcgtagttatctacacgacggggagtcaggcaac
tatggatgaacgaaatagacagatcgctgagataggtgcctcactgattaagcattggtaattcgaaatgaccgaccaagcgacgcc
caaccggtatcagctcactcaaaggcggtaatacggttatccacagaatcaggggataacgcaggaaagaacatgtgagcaaaa
ggccagcaaaaggccaggaaccgtaaaaaggccgcgttgctggcgtttttccataggctccgcccccctgacgagcatcacaaaa
atcgacgctcaagtcagaggtggcgaaacccgacaggactataaagataccaggcgtttccccctggaagctccctcgtgcgctctc
ctgttccgaccctgccgcttaccggatacctgtccgcctttctcccttcgggaagcgtggcgctttctcatagctcacgctgtaggtatctca
gttcggtgtaggtcgttcgctccaagctgggctgtgtgcacgaaccccccgttcagcccgaccgctgcgccttatccggtaactatcgtct
tgagtccaacccggtaagacacgacttatcgccactggcagcagccactggtaacaggattagcagagcgaggtatgtaggcggtg
ctacagagttcttgaagtggtggcctaactacggctacactagaaggacagtatttggtatctgcgctctgctgaagccagttaccttcgg
aaaaagagttggtagctcttgatccggcaaacaaaccaccgctggtagcggtggtttttttgtttgcaagcagcagattacgcgcagaa
aaaaaggatttcaagaagatcctttgatcttttctacggggtctgacgctcagtggaacgaaaactcacgttaagggattttggtcatgag
attatcaaaaaggatcttcacctagatccttttatagtccggaaatacaggaacgcacgctggatggcccttcgctgggatggtgaaac
catgaaaaatggcagcttcagtggattaagtgggggtaatgtggcctgtaccctctggttgcataggtattcatacggttaaaatttatca
ggcgcgattgcggcagtttttcgggtggtttgttgccatttttacctgtctgctgccgtgatcgcgctgaacgcgttttagcggtgcgtacaatt
aagggattatggtaaatccacttactgtctgccctcgtagccatcgagataaaccgcagtactccggccacgatgcgtccggcgtaga
ggatcgagatcttcaatattggccattagccatattattcattggttatatagcataaatcaatattggctattggccattgcatacgttgtatct
atatcataatatgtacatttatattggctcatgtccaatatgaccgccatgttggcattgattattgactagttattaatagtaatcaattacgg
ggtcattagttcatagcccatatatggagttccgcgttacataacttacggtaaatggcccgcctggctgaccgcccaacgacccccgc
ccattgacgtcaataatgacgtatgttcccatagtaacgccaatagggactttccattgacgtcaatgggtggagtatttacggtaaactg
cccacttggcagtacatcaagtgtatcatatgccaagtccgccccctattgacgtcaatgacggtaaatggcccgcctggcattatgcc
cagtacatgaccttacgggactttcctacttggcagtacatctacgtattagtcatcgctattaccatggtgatgcggttttggcagtacacc
aatgggcgtggatagcggtttgactcacggggatttccaagtctccaccccattgacgtcaatgggagtttgttttggcaccaaaatcaa

cgggactttccaaaatgtcgtaataaccccgccccgttgacgcaaatgggcggtaggcgtgtacggtgggaggtctatataagcaga
gctggtttagtgaaccgtcagatcactagaagctttattgcggtagtttatcacagttaaattgctaacgcagtcagtgcttctgacacaac
agtctcgaacttaagctgcagaagttggtcgtgaggcactgggcaggtaagtatcaaggttacaagacaggtttaaggagaccaata
gaaactgggcttgtcgagacagagaagactcttgcgtttctgataggcacctattggtcttactgacatccactttgcctttctctccacag
gtgtccactcccagttcaattacagctcttaaggctagagtattaatacgactcactatagggctagcaaagccaccatggcagaaatc
ggtactggctttccattcgacccccattatgtggaagtcctgggcgagcgcatgcactacgtcgatgttggtccgcgcgatggcacccct
gtgctgttcctgcacggtaacccgacctcctcctacgtgtggcgcaacatcatcccgcatgttgcaccgacccatcgctgcattgctcca
gacctgatcggtatgggcaaatccgacaaaccagacctgggttatttcttcgacgaccacgtccgcttcatggatgccttcatcgaagc
cctgggtctggaagaggtcgtcctggtcattcacgactggggctccgctctgggtttccactggccaagcgcaatccagagcgcgtc
aaaggtattgcatttatggagttcatccgccctatcccgacctgggacgaatggccagaatttgcccgcgagaccttccaggccttccg
caccaccgacgtcggccgcaagctgatcatcgatcagaacgtttttatcgagggtacgctgccgatgggtgtcgtccgcccgctgact
gaagtcgagatggaccattaccgcgagccgttcctgaatcctgttgaccgcgagccactgtggcgcttcccaaacgagctgccaatc
gccggtgagccagcgaacatcgtcgcgctggtcgaagaatacatggactggctgcaccagtcccctgtcccgaagctgctgttctgg
ggcaccccaggcgttctgatcccaccggccgaagccgctcgcctggccaaaagcctgcctaactgcaaggctgtggacatcggcc
cgggtctgaatctgctgcaagaagacaacccggacctgatcggcagcgagatcgcgcgctggctgtcgacgctcgagatttccggc
gagccaaccactgaggatctgtactttcagagcgataacgcgatcgcttccgag**atggctgtttccgtcacaccaattcgggacac**
aaaatggctaacactggaagtatgtagagagttccagaggggacttgctcacggccagacacggaatgtaaatttgcac
atccttcgaaaagctgccaagttgaaaatggacgagtaatcgcctgctttgattcattgaaaggccgttgctccagggagaa
ctgcaaatatcttcatccacccccacatttaaaaacgcagttggagataaatggacgcaataacttgattcagcagaagaacat
ggccatgttggcccagcaaatgcaactagccaatgccatgatgcctggtgccccattacaacccgtgccaatgttttcagttgcaccaa
gcttagccaccaatgcatcagcagccgcctttaatccctatctgggacctgtttctccaagcctggtcccggcagagatcttgccgactg
caccaatgttggttacagggaatccgggtgtccctgtacctgcagctgctgcagctgctgcacagaaattaatgcgaacagacagact
tgaggtatgtcgagagtaccaacgtggcaattgcaaccgaggagaaaatgattgtcggtttgctcatcctgctgacagcacaatgattg
acaccaatgacaacacagtcactgtgtgtatggattacatcaaagggagatgctctcgggaaaagtgcaaatactttcatcccoctgc
acatttgcaagccaagatcaaggctgcccaatacca

NLS*-MBNL1**-HaloTag plasmid sequence (nucleotides that encode HaloTag are underlined; nucleotides that encode **MBNL1** are in **bold**; nucleotides that encode the ***NLS are in ***bold and italics***):

cgggacacaaaatggctaacactggaagtatgtagagagttccagaggggacttgctcacggccagacacggaatgta
aatttgcacatccttcgaaaagctgccaagttgaaaatggacgagtaatcgcctgctttgattcattgaaaggccgttgctcc
agggagaactgcaaatatcttcatccacccccacatttaaaaacgcagttggagataaatggacgcaataacttgattcagc
agaagaacatggccatgttggcccagcaaatgcaactagccaatgccatgatgcctggtgccccattacaacccgtgccaatgttttc
agttgcaccaagcttagccaccaatgcatcagcagccgcctttaatccctatctgggacctgtttctccaagcctggtcccggcagagat
cttgccgactgcaccaatgttggttacagggaatccgggtgtccctgtacctgcagctgctgcagctgctgcacagaaattaatgcgaa
cagacagacttgaggtatgtcgagagtaccaacgtggcaattgcaaccgaggagaaaatgattgtcggtttgctcatcctgctgacag
cacaatgattgacaccaatgacaacacagtcactgtgtgtatggattacatcaaagggagatgctctcgggaaaagtgcaaatacttt
catcccoctgcacatttgcaagccaagatcaaggctgcccaataccacctaccgcggatatctagatttgggcccaattcctgcaggc
gagctctcgagccaaccactgaggatctgtactttcagagcgataacgatggatccgaaatcggtactggctttccattcgacccccatt
atgtggaagtcctgggcgagcgcatgcactacgtcgatgttggtccgcgcgatggcacccctgtgctgttcctgcacggtaacccgac
ctcctcctacgtgtggcgcaacatcatcccgcatgttgcaccgacccatcgctgcattgctccagacctgatcggtatgggcaaatccg
acaaaccagacctgggttatttcttcgacgaccacgtccgcttcatggatgccttcatcgaagccctgggtctggaagaggtcgtcctgg
tcattcacgactggggctccgctctgggtttccactggccaagcgcaatccagagcgcgtcaaaggtattgcatttatggagttcatcc
gccctatcccgacctgggacgaatggccagaatttgcccgcgagaccttccaggccttccgcaccaccgacgtcggccgcaagctg
atcatcgatcagaacgtttttatcgagggtacgctgccgatgggtgtcgtccgcccgctgactgaagtcgagatggaccattaccgcga
gccgttcctgaatcctgttgaccgcgagccactgtggcgcttcccaaacgagctgccaatcgccggtgagccagcgaacatcgtcgc
gctggtcgaagaatacatggactggctgcaccagtcccctgtcccgaagctgctgttctggggcaccccaggcgttctgatcccaccg
gccgaagccgctcgcctggccaaaagcctgcctaactgcaaggctgtggacatcggcccgggtctgaatctgctgcaagaagaca
acccggacctgatcggcagcgagatcgcgcgctggctgtctactctggagatttccggttaatagaattggcatgcaagctgatccgg

ctgctaacaaagcccgaaaggaagctgagttggctgctgccaccgctgagcaataactagcataaccccttggggcggccgcttcg
agcagacatgataagatacattgatgagtttggacaaaccacaactagaatgcagtgaaaaaaatgctttatttgtgaaatttgtgatgc
tattgctttatttgtaaccattataagctgcaataaacaagttaacaacaacaattgcattcattttatgtttcaggttcagggggagatgtgg
gaggttttttaagcaagtaaaacctctacaaatgtggtaaaatcgaattttaacaaaatattaacgcttacaatttcctgatgcggtattttc
tccttacgcatctgtgcggtatttcacaccgcatacgcggatctgcgcagcaccatggcctgaaataacctctgaaagaggaacttggtt
aggtaccttctgaggcggaaagaaccagctgtggaatgtgtgtcagttagggtgtggaaagtccccaggctccccagcaggcagaa
gtatgcaaagcatgcatctcaattagtcagcaaccaggtgtggaaagtccccaggctccccagcaggcagaagtatgcaaagcatg
catctcaattagtcagcaaccatagtcccgcccctaactccgcccatcccgcccctaactccgcccagttccgcccattctccgccccat
ggctgactaattttttttatttatgcagaggccgaggccgcctcggcctctgagctattccagaagtagtgaggaggcttttttggaggcct
aggcttttgcaaaaagcttgattcttctgacacaacagtctcgaacttaaggctagagccaccatgattgaacaagatggattgcacgc
aggttctccggccgcttgggtggagaggctattcggctatgactgggcacaacagacaatcggctgctctgatgccgccgtgttccggc
tgtcagcgcaggggcgcccggttctttttgtcaagaccgacctgtccggtgccctgaatgaactgcaggacgaggcagcgcggctatc
gtggctggccacgacgggcgttccttgcgcagctgtgctcgacgttgtcactgaagcgggaagggactggctgctattgggcgaagtg
ccggggcaggatctcctgtcatctcaccttgctcctgccgagaaagtatccatcatggctgatgcaatgcggcggctgcatacgcttgat
ccggctacctgcccattcgaccaccaagcgaaacatcgcatcgagcgagcacgtactcggatggaagccggtcttgtcgatcagga
tgatctggacgaagagcatcaggggctcgcgccagccgaactgttcgccaggctcaaggcgcgcatgcccgacggcgaggatctc
gtcgtgacccatggcgatgcctgcttgccgaatatcatggtggaaaatggccgcttttctggattcatcgactgtggccggctgggtgtgg
cggaccgctatcaggacatagcgttggctacccgtgatattgctgaagagcttggcggcgaatgggctgaccgcttcctcgtgctttac
ggtatcgccgctcccgattcgcagcgcatcgccttctatcgccttcttgacgagttcttctgagcgggactctggggttcgaaatgaccga
ccaagcgacgcccaacctgccatcacgatggccgcaataaaatatctttattttcattacatctgtgtgttggttttttgtgtgaatcgatagc
gataaggatcctctttgcgcttgcgttttcccttgtccagatagcccagtagctgacattcatccggggtcagcaccgtttctgcggactgg
ctttctacgtaatggtttcttagacgtcaggtggcacttttcggggaaatgtgcgcggaacccctatttgtttatttttctaaatacattcaaata
tgtatccgctcatgagacaataaccctgataaatgcttcaataatattgaaaaaggaagagtatgagtattcaacatttccgtgtcgccct
tattcccttttttgcggcattttgccttcctgtttttgctcacccagaaacgctggtgaaagtaaaagatgctgaagatcagttgggtgcacga
gtgggttacatcgaactggatctcaacagcggtaagatccttgagagttttcgccccgaagaacgttttccaatgatgagcactttcaaa
gttctgctatgtggcgcggtattatcccgtattgacgccgggcaagagcaactcggtcgccgcatacactattctcagaatgacttggttg
agtactcaccagtcacagaaaagcatcttacggatggcatgacagtaagagaattatgcagtgctgccataaccatgagtgataaca
ctgcggccaacttacttctgacaactatcggaggaccgaaggagctaaccgcttttttgcacaacatgggggatcatgtaactcgcctt
gatcgttgggaaccggagctgaatgaagccataccaaacgacgagcgtgacaccacgatgcctgtagcaatggcaacaacgttgc
gcaaactattaactggcgaactacttactctagcttcccggcaacaattaatagactggatggaggcggataaagttgcaggaccactt
ctgcgctcggcccttccggctggctggtttattgctgataaatctggagccggtgagcgtgggtctcgcggtatcattgcagcactgggg
ccagatggtaagccctcccgtatcgtagttatctacacgacggggagtcaggcaactatggatgaacgaaatagacagatcgctgag
ataggtgcctcactgattaagcattggtaattcgaaatgaccgaccaagcgacgcccaaccggtatcagctcactcaaaggcggtaa
tacggttatccacagaatcaggggataacgcaggaaagaacatgtgagcaaaaggccagcaaaaggccaggaaccgtaaaaa
ggccgcgttgctggcgtttttccataggctccgcccccctgacgagcatcacaaaaatcgacgctcaagtcagaggtggcgaaaccc
gacaggactataaagataccaggcgtttccccctggaagctccctcgtgcgctctcctgttccgaccctgccgcttaccggatacctgtc
cgcctttctcccttcgggaagcgtggcgctttctcatagctcacgctgtaggtatctcagttcggtgtaggtcgttcgctccaagctgggctg
tgtgcacgaaccccccgttcagcccgaccgctgcgccttatccggtaactatcgtcttgagtccaacccggtaagacacgacttatcgc
cactggcagcagccactggtaacaggattagcagagcgaggtatgtaggcggtgctacagagttcttgaagtggtggcctaactacg
gctacactagaaggacagtatttggtatctgcgctctgctgaagccagttaccttcggaaaaagagttggtagctcttgatccggcaaac
aaaccaccgctggtagcggtggtttttttgtttgcaagcagcagattacgcgcagaaaaaaaggatttcaagaagatcctttgatcttttct
acggggtctgacgctcagtggaacgaaaactcacgttaagggattttggtcatgagattatcaaaaaggatcttcacctagatccttttat
agtccggaaatacaggaacgcacgctggatggcccttcgctgggatggtgaaaccatgaaaaatggcagcttcagtggattaagtg
gggtaatgtggcctgtaccctctggttgcataggtattcatacggttaaaatttatcaggcgcgattgcggcagtttttcgggtggtttgttg
ccatttttacctgtctgctgccgtgatcgcgctgaacgcgttttagcggtgcgtacaattaagggattatggtaaatccacttactgtctgcc
ctcgtagccatcgagataaaccgcagtactccggccacgatgcgtccggcgtagaggatcgagatcttcaatattggccattagccat
attattcattggttatatagcataaatcaatattggctattggccattgcatacgttgtatctatatcataatatgtacatttatattggctcatgtc
caatatgaccgccatgttggcattgattattgactagttattaatagtaatcaattacggggtcattagttcatagcccatatatggagttccg
cgttacataacttacggtaaatggcccgcctggctgaccgcccaacgacccccgcccattgacgtcaataatgacgtatgttcccatag
taacgccaatagggactttccattgacgtcaatgggtggagtatttacggtaaactgcccacttggcagtacatcaagtgtatcatatgcc
aagtccgccccctattgacgtcaatgacggtaaatggcccgcctggcattatgcccagtacatgaccttacgggactttcctacttggca

gtacatctacgtattagtcatcgctattaccatggtgatgcggttttggcagtacaccaatgggcgtggatagcggtttgactcacgggga
tttccaagtctccaccccattgacgtcaatgggagtttgttttggcaccaaaatcaacgggactttccaaaatgtcgtaataaccccgccc
cgttgacgcaaatgggcggtaggcgtgtacggtgggaggtctatataagcagagctggtttagtgaaccgtcagatcactagaagcttt
attgcggtagtttatcacagttaaattgctaacgcagtcagtgcttctgacacaacagtctcgaacttaagctgcagaagttggtcgtgag
gcactgggcaggtaagtatcaaggttacaagacaggtttaaggagaccaatagaaactgggcttgtcgagacagagaagactcttg
cgtttctgataggcacctattggtcttactgacatccactttgcctttctctccacaggtgtccactcccagttcaattacagctcttaaggcta
gagtattaatacgactcactatagggctagcaaagcgatcgcttccgatg***cccaaaaagaaaagaaaggtggctgtttccgtcac***
accaatt

NLS*-MBNL1**-NanoLuc plasmid sequence (nucleotides that encode NanoLuc are underlined; nucleotides that encode **MBNL1** are in **bold**; nucleotides that encode the ***NLS are in ***bold and italics***):

cgggacacaaaatggctaacactggaagtatgtagagagttccagagggggacttgctcacggccagacacggaatgta
aatttgcacatccttcgaaaagctgccaagttgaaaatggacgagtaatcgcctgctttgattcattgaaaggccgttgctcc
agggagaactgcaaatatcttcatccacccccacatttaaaaacgcagttggagataaatggacgcaataacttgattcagc
agaagaacatggccatgttggcccagcaaatgcaactagccaatgccatgatgcctggtgccccattacaacccgtgccaatgtttc
agttgcaccaagcttagccaccaatgcatcagcagccgcctttaatccctatctgggacctgtttctccaagcctggtcccggcagagat
cttgccgactgcaccaatgttggttacagggaatccgggtgtccctgtacctgcagctgctgcagctgctgcacagaaattaatgcgaa
cagacagacttgaggtatgtcgagagtaccaacgtggcaattgcaaccgaggagaaaatgattgtcggtttgctcatcctgctgacag
cacaatgattgacaccaatgacaacacagtcactgtgtgtatggattacatcaaagggagatgctctcgggaaaagtgcaaatacttt
catccccctgcacatttgcaagccaagatcaaggctgcccaataccacctaccgcggatatctagatttgggcccaattcctgcaggc
gagctctcggctcgagcggcgtcttcacactcgaagatttcgttggggactggcgacagacagccggctacaacctggaccaagtcc
ttgaacagggaggtgtgtccagtttgtttcagaatctcggggtgtccgtaactccgatccaaaggattgtcctgagcggtgaaaatgggc
tgaagatcgacatccatgtcatcatcccgtatgaaggtctgagcggcgaccaaatgggccagatcgaaaaaatttttaaggtggtgta
ccctgtggatgatcatcactttaaggtgatcctgcactatggcacactggtaatcgacggggttacgccgaacatgatcgactatttcgg
acggccgtatgaaggcatcgccgtgttcgacggcaaaaagatcactgtaacagggaccctgtggaacggcaacaaaattatcgac
gagcgcctgatcaaccccgacggctccctgctgttccgagtaaccatcaacggagtgaccggctggcggctgtgcgaacgcattctg
gcgtaaggccgcgactctagggcatgcaagctgatccggctgctaacaaagcccgaaaggaagctgagttggctgctgccaccgct
gagcaataactagcataaccccttggggcggccgcttcgagcagacatgataagatacattgatgagtttggacaaaccacaactag
aatgcagtgaaaaaaatgctttatttgtgaaatttgtgatgctattgctttatttgtaaccattataagctgcaataaacaagttaacaacaa
caattgcattcattttatgtttcaggttcagggggagatgtgggaggttttttaagcaagtaaaacctctacaaatgtggtaaaatcgaattt
taacaaaatattaacgcttacaatttcctgatgcggtattttctccttacgcatctgtgcggtatttcacaccgcatacgcggatctgcgcag
caccatggcctgaaataacctctgaaagaggaacttggttaggtaccttctgaggcggaaagaaccagctgtggaatgtgtgtcagtt
agggtgtggaaagtccccaggctccccagcaggcagaagtatgcaaagcatgcatctcaattagtcagcaaccaggtgtggaaagt
ccccaggctccccagcaggcagaagtatgcaaagcatgcatctcaattagtcagcaaccatagtcccgcccctaactccgcccatcc
cgcccctaactccgcccagttccgcccattctccgccccatggctgactaattttttttatttatgcagaggccgaggccgcctcggcctct
gagctattccagaagtagtgaggaggcttttttggaggcctaggcttttgcaaaaagctcgattcttctgacactagcgccaccatgaag
aagcccgaactcaccgctaccagcgttgaaaaatttctcatcgagaagttcgacagtgtgagcgacctgatgcagttgtcggagggc
gaagagagccgagccttcagcttcgatgtcggcggacgcggctatgtactgcgggtgaatagctgcgctgatggcttctacaaagac
cgctacgtgtaccgccacttcgccagcgctgcactacccatccccgaagtgttggacatcggcgagttcagcgagagcctgacatact
gcatcagtagacgcgcccaaggcgttactctccaagacctccccgaaacagagctgcctgctgtgttacagcctgtcgccgaagctat
ggatgctattgccgccgccgacctcagtcaaaccagcggcttcggcccattcgggccccaaggcatcggccagtacacaacctggc
gggatttcatttgcgccattgctgatccccatgtctaccactggcagaccgtgatggacgacaccgtgtccgccagcgtagctcaagcc
ctggacgaactgatgctgtgggccgaagactgtcccgaggtgcgccacctcgtccatgccgacttcggcagcaacaacgtcctgacc
gacaacggccgcatcaccgccgtaatcgactggtccgaagctatgttcggggacagtcagtacgaggtggccaacatcttcttctggc
ggccctggctggcttgcatggagcagcagactcgctacttcgagcgccggcatcccgagctggccggcagccctcgtctgcgagcct
acatgctgcgcatcggcctggatcagctctaccagagcctcgtggacggcaacttcgacgatgctgcctgggctcaaggccgctgcg
atgccatcgtccgcagcggggccggcaccgtcggtcgcacacaaatcgctcgccggagcgcagccgtatggaccgacggctgcgt
cgaggtgctggccgacagcggcaaccgccggcccagtacacgaccgcgcgctaaggaggtaggtcgagtttagactctagccatc
acgatggccgcaataaaatatctttattttcattacatctgtgtgttggtttttgtgtgaatcgatagcgataaggatcctctttgcgcttgcgttt

tcccttgtccagatagcccagtagctgacattcatccggggtcagcaccgtttctgcggactggctttctacgtaatggtttcttagacgtca
ggtggcacttttcggggaaatgtgcgcggaacccctatttgtttatttttctaaatacattcaaatatgtatccgctcatgagacaataaccct
gataaatgcttcaataatattgaaaaaggaagagtatgagtattcaacatttccgtgtcgcccttattcccttttttgcggcattttgccttcct
gtttttgctcacccagaaacgctggtgaaagtaaaagatgctgaagatcagttgggtgcacgagtgggttacatcgaactggatctcaa
cagcggtaagatccttgagagttttcgccccgaagaacgttttccaatgatgagcacttttaaagttctgctatgtggcgcggtattatccc
gtattgacgccgggcaagagcaactcggtcgccgcatacactattctcagaatgacttggttgagtactcaccagtcacagaaaagc
atcttacggatggcatgacagtaagagaattatgcagtgctgccataaccatgagtgataacactgcggccaacttacttctgacaact
atcggaggaccgaaggagctaaccgcttttttgcacaacatgggggatcatgtaactcgccttgatcgttgggaaccggagctgaatg
aagccataccaaacgacgagcgtgacaccacgatgcctgtagcaatggcaacaacgttgcgcaaactattaactggcgaactactt
actctagcttcccggcaacaattaatagactggatggaggcggataaagttgcaggaccacttctgcgctcggcccttccggctggctg
gtttattgctgataaatctggagccggtgagcgtgggtctcgcggtatcattgcagcactggggccagatggtaagccctcccgtatcgt
agttatctacacgacggggagtcaggcaactatggatgaacgaaatagacagatcgctgagataggtgcctcactgattaagcattg
gtaattcgaaatgaccgaccaagcgacgcccaaccggtatcagctcactcaaaggcggtaatacggttatccacagaatcagggg
ataacgcaggaaagaacatgtgagcaaaaggccagcaaaaggccaggaaccgtaaaaaggccgcgttgctggcgtttttccata
ggctccgcccccctgacgagcatcacaaaaatcgacgctcaagtcagaggtggcgaaacccgacaggactataaagataccagg
cgtttccccctggaagctccctcgtgcgctctcctgttccgaccctgccgcttaccggatacctgtccgcctttctcccttcgggaagcgtg
gcgctttctcatagctcacgctgtaggtatctcagttcggtgtaggtcgttcgctccaagctgggctgtgtgcacgaaccccccgttcagc
ccgaccgctgcgccttatccggtaactatcgtcttgagtccaacccggtaagacacgacttatcgccactggcagcagccactggtaa
caggattagcagagcgaggtatgtaggcggtgctacagagttcttgaagtggtggcctaactacggctacactagaaggacagtattt
ggtatctgcgctctgctgaagccagttaccttcggaaaaagagttggtagctcttgatccggcaaacaaaccaccgctggtagcggtg
gtttttttgtttgcaagcagcagattacgcgcagaaaaaaaggatttcaagaagatcctttgatcttttctacggggtctgacgctcagtgg
aacgaaaactcacgttaagggattttggtcatgagattatcaaaaaggatcttcacctagatccttttatagtccggaaatacaggaacg
cacgctggatggcccttcgctgggatggtgaaaccatgaaaaatggcagcttcagtggattaagtgggggtaatgtggcctgtaccct
ctggttgcataggtattcatacggttaaaatttatcaggcgcgattgcggcagtttttcgggtggtttgttgccatttttacctgtctgctgccgt
gatcgcgctgaacgcgttttagcggtgcgtacaattaagggattatggtaaatccacttactgtctgccctcgtagccatcgagataaac
cgcagtactccggccacgatgcgtccggcgtagaggatcgagatcttcaatattggccattagccatattattcattggttatatagcata
aatcaatattggctattggccattgcatacgttgtatctatatcataatatgtacatttatattggctcatgtccaatatgaccgccatgttggc
attgattattgactagttattaatagtaatcaattacggggtcattagttcatagcccatatatggagttccgcgttacataacttacggtaaa
tggcccgcctggctgaccgcccaacgacccccgcccattgacgtcaataatgacgtatgttcccatagtaacgccaatagggactttc
cattgacgtcaatgggtggagtatttacggtaaactgcccacttggcagtacatcaagtgtatcatatgccaagtccgccccctattgac
gtcaatgacggtaaatggcccgcctggcattatgcccagtacatgaccttacgggactttcctacttggcagtacatctacgtattagtca
tcgctattaccatggtgatgcggttttggcagtacaccaatgggcgtggatagcggtttgactcacggggatttccaagtctccacccat
tgacgtcaatgggagtttgttttggcaccaaaatcaacgggactttccaaaatgtcgtaataaccccgccccgttgacgcaaatgggcg
gtaggcgtgtacggtgggaggtctatataagcagagctggtttagtgaaccgtcagatcactagaagctttattgcggtagtttatcaca
gttaaattgctaacgcagtcagtgcttctgacacaacagtctcgaacttaagctgcagaagttggtcgtgaggcactgggcaggtaagt
atcaaggttacaagacaggtttaaggagaccaatagaaactgggcttgtcgagacagagaagactcttgcgtttctgataggcaccta
ttggtcttactgacatccactttgcctttctctccacaggtgtccactcccagttcaattacagctcttaaggctagagtattaatacgactca
ctatagggctagcaaagcgatcgcttccg***atgcccaaaaagaaaagaaaggtggctgtttccgtcacaccaatt***

**Table S2**. Sequences of primers used in these studies.		
Primers and probes used for RT-qPCR amplification of HeLa cells stably expressing mutant and WT alleles (HeLa480)[1]		
**Primers**	**Forward Sequence (5'→3')**	**Reverse Sequence (5'→3')**
HeLa_CUG (RT-qPCR)	CGATCTCTGCCTGCTTACTC	GTCGGAGGACGAGGTCAATAAA
GAPDH (SYBR)	GAAGGTGAAGGTCGGAGTC	GAAGATGGTGATGGGATTTC
**Probe**	**Sequence (5'→3')**	
Probe 1 (WT allele)	/56FAM/AGAGCAGCG/ZEN/CAAGTGAGGAGG/3IABkFQ/	
Probe 2 (mutant allele)	/5HEX/TGACGCAGC/ZEN/CACGTGAAGGTC/3IABkFQ/	
Primers used in MyoD-inducible WT and DM1 myotubes		
	**Forward Sequence (5'→3')**	**Reverse Sequence (5'→3')**
DMPK	CGT GCA AGC GCC CAG	CTC CAC CAA CTT ACT GTT TCA TCC T
MBNL1 exon 5 inclusion and exclusion	GCT GCC CAA TAC CAG GTC AAC	TGG TGG GAG AAA TGC TGT ATG C
MyoD	CGG CAT GAT GGA CTA CAG CG	CAG GCA GTC TAG GCT CGA C

**Table S3: Sequences of smFISH probe targeting *DMPK*_exons 11-15 for imaging foci in HeLa480 cells.**		
**Probe ID**	**Sequence (5' → 3')**	**Targeting nucleotide sequence (5' → 3') and nt range in *DMPK***
DMPK_Exon11-15_1	gaaagcgcctccgatagg	cctatcggaggcgctttc; 1203-1220
DMPK_Exon11-15_2	acggcgaacaggagcagg	cctgctcctgttcgccgt; 1221-1238
DMPK_Exon11-15_3	ggcggcacgagacagaac	gttctgtctcgtgccgcc; 1240-1257
DMPK_Exon11-15_4	caacccaatgcagcccag	ctgggctgcattgggttg; 1261-1278
DMPK_Exon11-15_5	cagactgcggtgagttgg	ccaactcaccgcagtctg; 1293-1310
DMPK_Exon11-15_6	tctagggttcagggagcg	cgctccctgaaccctaga; 1332-1349
DMPK_Exon11-15_7	cccggagtcgaagacagt	actgtcttcgactccggg; 1350-1367
DMPK_Exon11-15_8	gcactcagtcttccaacg	cgttggaagactgagtgc; 1372-1389
DMPK_Exon11-15_9	gaactggcaggcggtggg	cccaccgcctgccagttc; 1414-1431
DMPK_Exon11-15_10	cacgctcggagcggttgt	acaaccgctccgagcgtg; 1432-1449
DMPK_Exon11-15_11	tcacaggactggagctgg	ccagctccagtcctgtga; 1459-1476
DMPK_Exon11-15_12	tacaaggacccttcgagc	gctcgaagggtccttgta; 1537-1557
DMPK_Exon11-15_13	tttgcccatccacgtcag	ctgacgtggatgggcaaa; 1668-1685
DMPK_Exon11-15_14	ggatggaacacggacggc	gccgtccgtgttccatcc; 1714-1731
DMPK_Exon11-15_15	aacgataggtgggggtgc	gcacccccacctatcgtt; 1737-1754
DMPK_Exon11-15_16	ctttgcactttgcgaacc	ggttcgcaaagtgcaaag; 1755-1772
DMPK_Exon11-15_17	cagggcgtcatgcacaag	cttgtgcatgacgccctg; 1777-1794
DMPK_Exon11-15_18	aagcaggcagagatcgcg	ccgcgatctctgcctgctt; 1812-1829
DMPK_Exon11-15_19	tcggaggacgaggtcaat	attgacctcgtcctccga; 1947-1964
DMPK_Exon11-15_20	ctgtagcctgtcagcgag	ctcgctgacaggctacag; 1965-1982
DMPK_Exon11-15_21	ttggggttgttgggggtc	gacccccaacaaccccaa; 1983-2000
DMPK_Exon11-15_22	gtgcatccaaaacgtgga	tccacgttttggatgcac; 2001-2018
DMPK_Exon11-15_23	aggaatgtcggggtctca	tgagaccccgacattcct; 2019-2036
DMPK_Exon11-15_24	tcctaggtggggacagac	gtctgtccccacctagga; 2048-2065
DMPK_Exon11-15_25	tttattcgcgagggtcgg	ccgaccctcgcgaataaa; 2075-2092
DMPK_Exon11-15_26	ttgggcagatggagggcc	ggccctccatctgcccaa; 2094-2111

Table S4 – IC_{50} values for cellular NanoBRET and in vitro TR-FRET assays for small molecule hits

Small molecule	IC_{50} of NanoBRET (μM)	IC_{50} of TR-FRET (μM)
**A1**	>50	14 ± 3
**A2**	22 ± 6	25 ± 1
**A3**	23 ± 5	>50
**A4**	39 ± 6	22 ± 1
**A5**	14 ± 2	>50
**A6**	27 ± 3	18 ± 2
**B1**	37 ± 12	>50
**B2**	>50	>50
**B3**	35 ± 12	>50
**B4**	22 ± 4	>50

**Table S5: Sequences of smFISH probe targeting the *DMPK* CDS to image foci in DM1 myotubes.**		
**Probe ID**	**Sequence (5' → 3')**	**Targeting nucleotide sequence (5' → 3') and nt range in *DMPK***
DMPK_CDS_1	aagaagtcggccacgtactt	aagtacgtggccgacttctt; 271-290
DMPK_CDS_2	cttaagcctcaccacgatgg	ccatcgtggtgaggcttaag; 2628-2647
DMPK_CDS_3	gatcaccttcagaatctcga	tcgagattctgaaggtgatc; 2673-2692
DMPK_CDS_4	gtctgcttcatcttcactac	gtagtgaagatgaagcagac; 2720-2739
DMPK_CDS_5	gttcatgatcttcatggcat	atgccatgaagatcatgaac; 2751-2770
DMPK_CDS_6	tcacggaagcacgacacctc	gaggtgtcgtgcttccgtga; 2795-2814
DMPK_CDS_7	cgaagtgcagctgcgtgatc	gatcacgcagctgcacttcg; 2851-2870
DMPK_CDS_8	tacaggtagttctcatcctg	caggatgagaactacctgta; 2876-2895
DMPK_CDS_9	cacgtaatactccatgacca	tggtcatggagtattacgtg; 2898-2917
DMPK_CDS_10	aaacttgctcagcagtgtca	tgacactgctgagcaagttt; 2931-2950
DMPK_CDS_11	agtctatggccatgacaatc	gattgtcatggccatagact; 2995-3014
DMPK_CDS_12	atgttgtcgggtttgatgtc	gacatcaaacccgacaacat; 3044-3063
DMPK_CDS_13	ttgaggcaagagccgaagtc	gacttcggctcttgcctcaa; 3098-3117
DMPK_CDS_14	ctgcccatagaacatttcat	atgaaatgttctatgggcag; 3267-3286
DMPK_CDS_15	cgtggaatccgcgtagaagg	ccttctacgcggattccacg; 3291-3310
DMPK_CDS_16	acgatcttgccataggtctc	gagacctatggcaagatcgt; 3314-3333
DMPK_CDS_17	gagagaggtgctccttgtag	ctacaaggagcacctctctc; 3337-3356
DMPK_CDS_18	agcaaccgctgaatgaagtc	gacttcattcagcggttgct; 3395-3414
DMPK_CDS_19	aagaagggatgtgtccggaa	ttccggacacatcccttctt; 3458-3477
DMPK_CDS_20	gaaatccggtgtaaaggggg	ccccctttacaccggatttc; 3516-3535
DMPK_CDS_21	caccaagtcgaagttgcatg	catgcaacttcgacttggtg; 3552-3571
DMPK_CDS_22	cgaatgtccgacagtgtctc	gagacactgtcggacattcg; 3608-3627
DMPK_CDS_23	catgcaggagtaggagtagc	gctactcctactcctgcatg; 3666-3685
DMPK_CDS_24	acttcagctgtttcatcctg	caggatgaaacagctgaagt; 3794-3813
DMPK_CDS_25	tagttgactggcgaagttct	agaacttcgccagtcaacta; 3951-3970
DMPK_CDS_26	catctagatgggaaggtgga	tccaccttcccatctagatg; 4096-4115
DMPK_CDS_27	gaacaggagcagggaaagcg	cgctttccctgctcctgttc; 4224-4243
DMPK_CDS_28	cggcacgagacagaacaacg	cgttgttctgtctcgtgccg; 4246-4265
DMPK_CDS_29	cggagtcgaagacagttcta	tagaactgtcttcgactccg; 4356-4375
DMPK_CDS_30	ctcggagcggttgtgaactg	cagttcacaaccgctccgag; 4436-4455
DMPK_CDS_31	gatcacaggactggagctgg	ccagctccagtcctgtgatc; 4469-4488

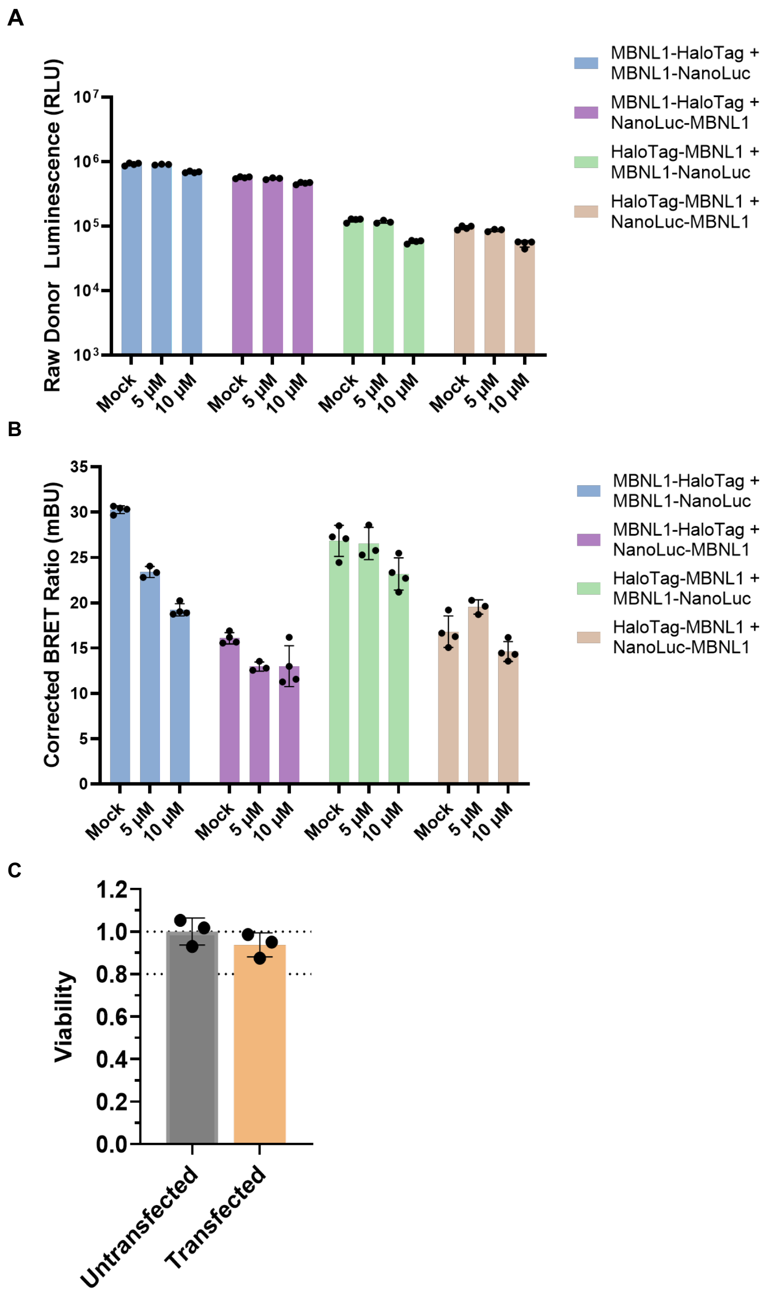

Figure S1. Corrected BRET ratios for different MBNL1 construct combinations in untreated HeLa480 cells (mock) and HeLa480 cells treated with CAG25 Vivo-Morpholino.

(A) Raw donor luminescence (Relative Luminescence Units, RLU) for each construct combination under mock and Vivo-Morpholino-treated conditions. The donor luminescence remained within an optimal range across all tested combinations (~1 × 10^5).

(B) HeLa480 cells were co-transfected with different combinations of MBNL1 constructs tagged with HaloTag and NanoLuc at the N- or C-terminus and evaluated in the NanoBRET assay. The experiment was conducted using a 50:1 ratio of plasmids encoding MBNL1 fused to either HaloTag or NanoLuc fusions at the indicated terminus, respectively. Cells were treated with 5 µM or 10 µM of CAG25 Vivo-Morpholino, an antisense oligonucleotide targeting r(CUG) repeats. The

greatest reduction in the NanoBRET signal was observed with the C-terminal fusions (MBNL1-HaloTag + MBNL1-NanoLuc) compared to untreated samples.

(C) Viability of HeLa480 cells after co-transfection of 50 ng of MBNL1-NanoLuc and 2500 ng MBNL1-HaloTag, as measured by CellTiter-Glo viability reagent.

Data are reported as the mean ± standard deviation (n = 3 biological replicates), with individual data points shown. These findings support the use of C-terminal fusions for optimal assay sensitivity and reproducibility.

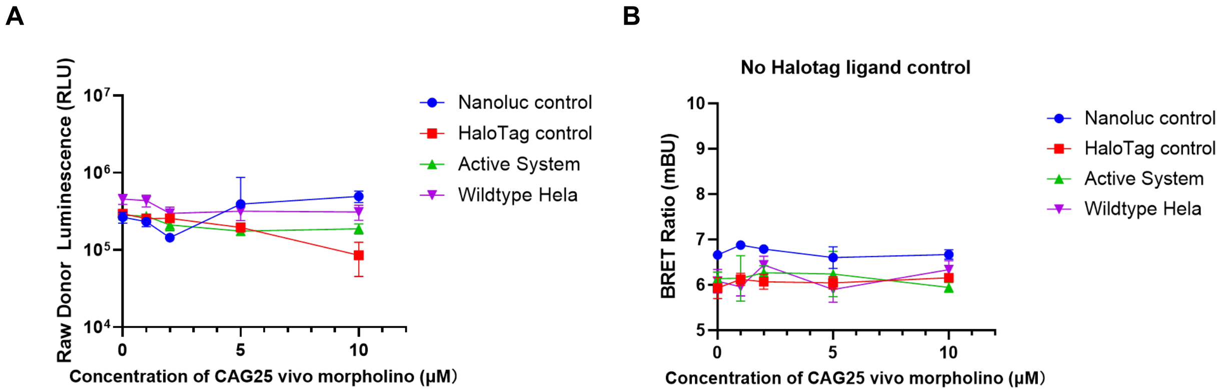

Figure S2: Donor Luminescence and background BRET ratios for NanoBRET constructs in HeLa480 cells.

(A) Raw donor luminescence (RLU) values were measured in control systems to confirm that all conditions maintained sufficient expression levels for reliable NanoBRET analysis: (i) HeLa480 cells transfected with MBNL1-HaloTag + NanoLuc (no MBNL1 fusion; blue); (ii) HeLa480 cells transfected with HaloTag (no MBNL1) + MBNL1-NanoLuc (red); and (iii) WT HeLa cells [no $r(CUG)^{exp}$] transfected with MBNL1-NanoLuc and MBNL1-HaloTag (purple). “Active System” indicates HeLa480 cells were transfected with MBNL1-NanoLuc and MBNL1-HaloTag (green). Cells were treated with increasing concentrations of CAG25 Vivo-Morpholino, and donor luminescence was recorded. Data are reported as the mean ± standard deviation (n = 3).

(B) BRET ratios were measured in control samples without the addition of the HaloTag NanoBRET 618 ligand to establish background levels in different control systems as defined in (A). Data represent mean ± standard deviation (n = 3).

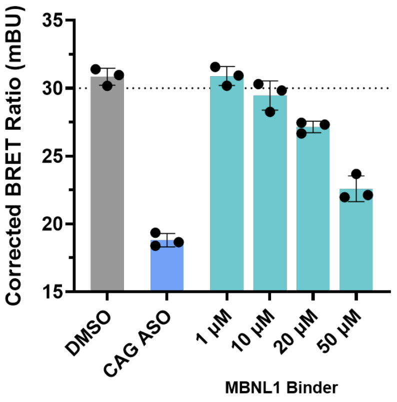

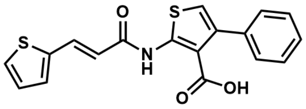

MBNL1 binder

Figure S3: Dose-response analysis and chemical structures of MBNL1 binder in NanoBRET assay.

Dose dependent reduction of the corrected BRET ratios (mBU) for a previously reported MBNL1 binder, the chemical structure of which is provided.[2] Data are reported as mean ± standard deviation (n = 3 biological replicates).

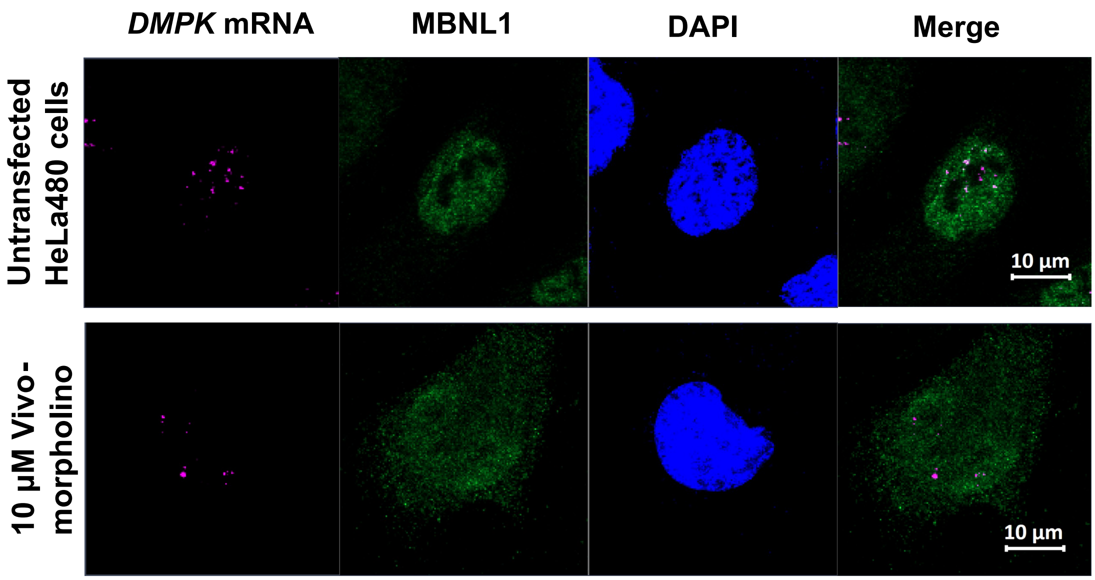

Figure S4. Disruption of nuclear foci containing r(CUG)$_{480}$ and endogenous MBNL1 upon treatment with CAG25 Vivo-Morpholino in HeLa480 cells.

Representative microscopy images of nuclear foci in untransfected HeLa480, where r(CUG)^{exp} was imaged by smFISH (magenta), endogenous MBNL1 was detected by immunofluorescence assay (IFA; anti-MBNL1 antibody, green), and nuclei were stained with DAPI (blue) under mock conditions (top row) and after treatment with 10 µM of CAG25 Vivo-Morpholino (bottom row). In untreated cells, endogenous MBNL1 co-localizes with r(CUG)^{exp} in nuclear foci, as observed in the merge channel (white signals). Scale bar is 10 µM.

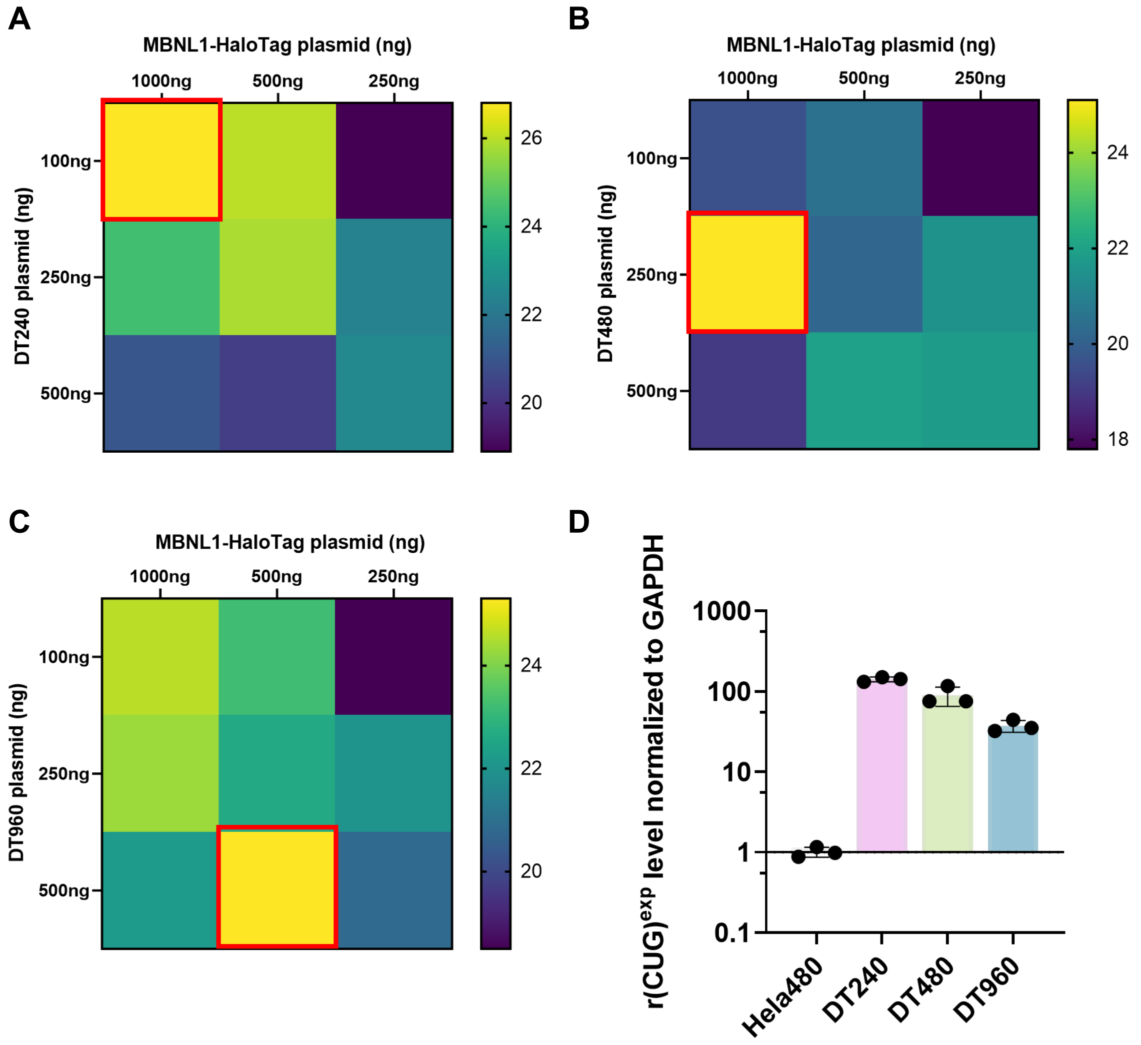

Figure S5. NanoBRET assay performance in HeLa cells transiently transfected with plasmid encoding interrupted repeats of varying lengths. Three plasmid constructs bearing interrupted r(CUG) repeats—DT960 (48 modules), DT480 (24 modules), and DT240 (12 modules) of the [$(CTG)_{(20)}$(CTCGA)] motif—were transiently expressed in HeLa cells alongside MBNL1-NanoLuc and MBNL1-HaloTag fusions.

(A-C) Heat maps summarizing assay window sizes for DT240 (**A**), DT480 (**B**), and DT960 (**C**). Window size is defined as the decrease in NanoBRET signal after treatment with 10 μM CAG25 Vivo-Morpholino, obtained at the indicated amounts of plasmids. MBNL1-NanoLuc plasmid was kept constant at 25 ng.

(D) Expression level of $r(CUG)^{exp}$ in HeLa cells transfected with plasmids encoding DT240, DT480, and DT960, as compared to HeLa480 cells, as determined by RT-qPCR (normalized to *GAPDH*; n = 3 biological replicates).

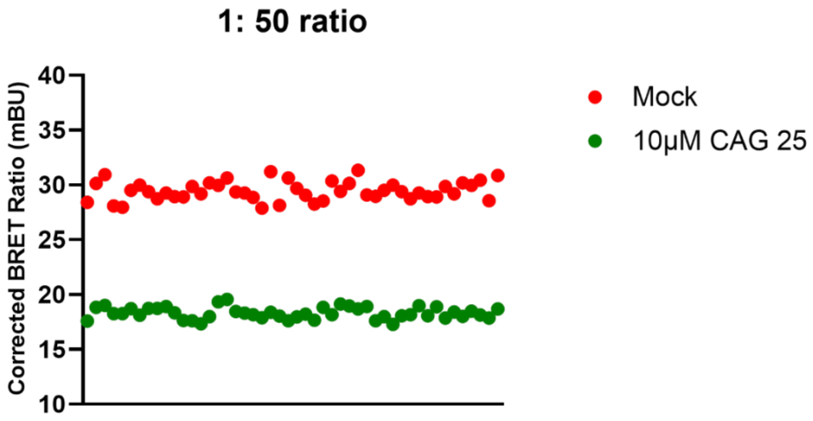

Figure S6. Assessment of NanoBRET assay suitability for high-throughput screening (HTS).

Corrected BRET ratios (mBU) are shown for a 1:50 ratio of plasmids encoding MBNL1-NanoLuc : MBNL1-HaloTag in the presence and absence of 10 μM of CAG25 Vivo-Morpholino (green) and in the mock-treated controls (red). To assess the suitability of the assay for high-throughput screening (HTS), the Z-factor was calculated at the 10 μM dose in a 96-well plate format. The Z-factor, [3] a measure of assay quality for HTS, was determined to be 0.63, indicating that the assay is suitable for HTS (Z-factor ≥ 0.5).

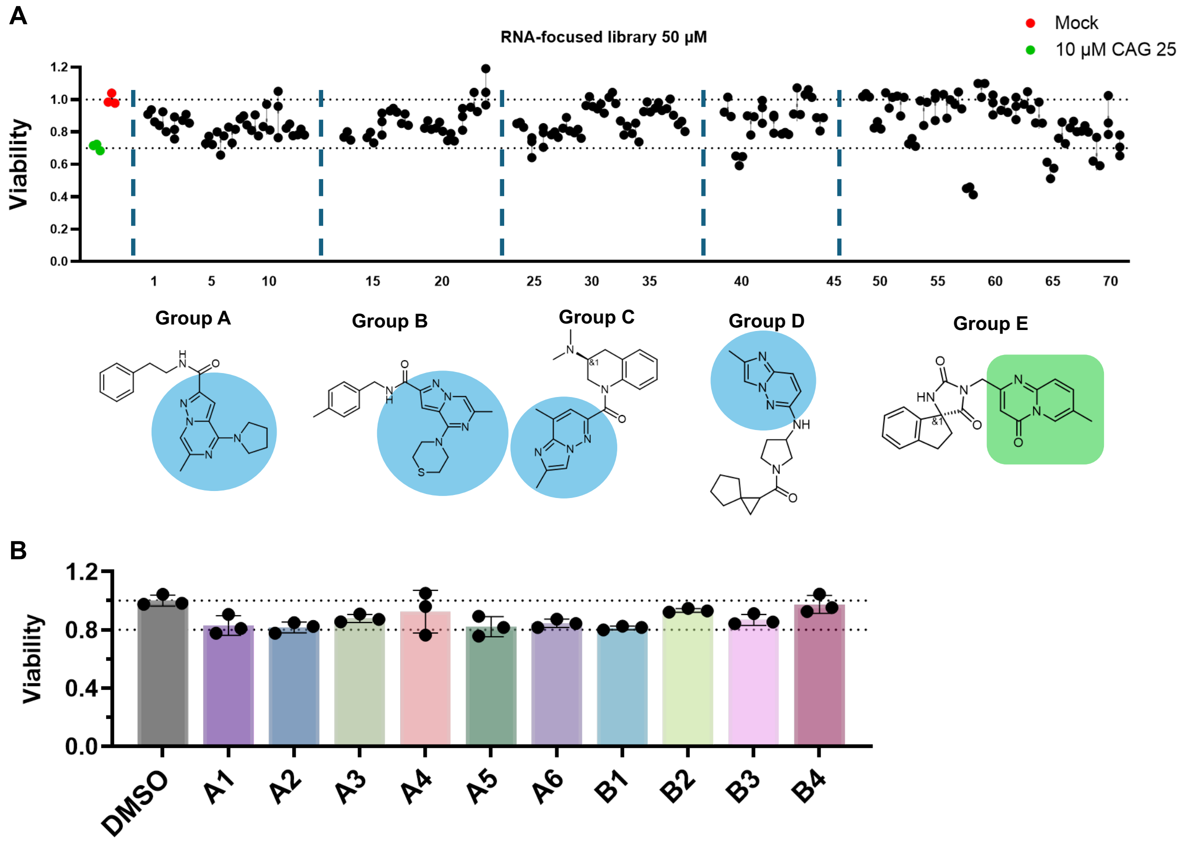

Figure S7. Effect of small molecules on the viability of HeLa480 cells.

(A) Cell viability assay performed in HeLa480 cells to assess cytotoxicity of small molecules identified from the NanoBRET screening. Small molecules were tested at 50 μM, and cell viability was measured using a luminescence-based ATP assay. Viability percentages are normalized to DMSO-treated cells (red). The viability of cells treated with 10 μM of CAG25 Vivo-Morpholino is indicated in green. Dashed lines are present at 100% and 70% viability. Compounds with viability below the 70% threshold were considered cytotoxic and deprioritized for further analysis.

(B) Cell viability from panel (A) for all ten hit compounds, each with >80% viability.

Data are reported as mean ± standard deviation (n = 3 biological replicates).

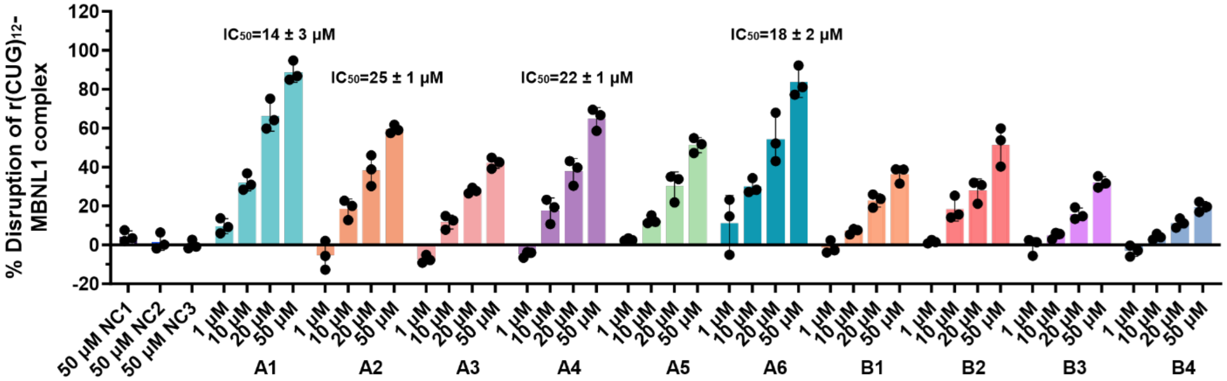

Figure S8. Dose-dependent disruption of r(CUG)$_{12}$-MBNL1 complex *in vitro* by small molecules in a TR-FRET assay.

The ability of small molecules **A1** – **A6** and **B1** – **B4** to disrupt the interaction between r(CUG)$_{12}$ and MBNL1 protein *in vitro* was assessed using a time-resolved fluorescence resonance energy transfer (TR-FRET) assay. Increasing concentrations of compounds (1 μM, 10 μM, 20 μM, and 50 μM) were tested. The percentage disruption of the r(CUG)$_{12}$-MBNL1 complex was calculated relative to untreated controls. **A1** and **A6** exhibited the most potent dose-dependent activity, achieving up to ~100% disruption at 50 μM. Data are reported as mean ± SD from (n = 3 independent experiments).

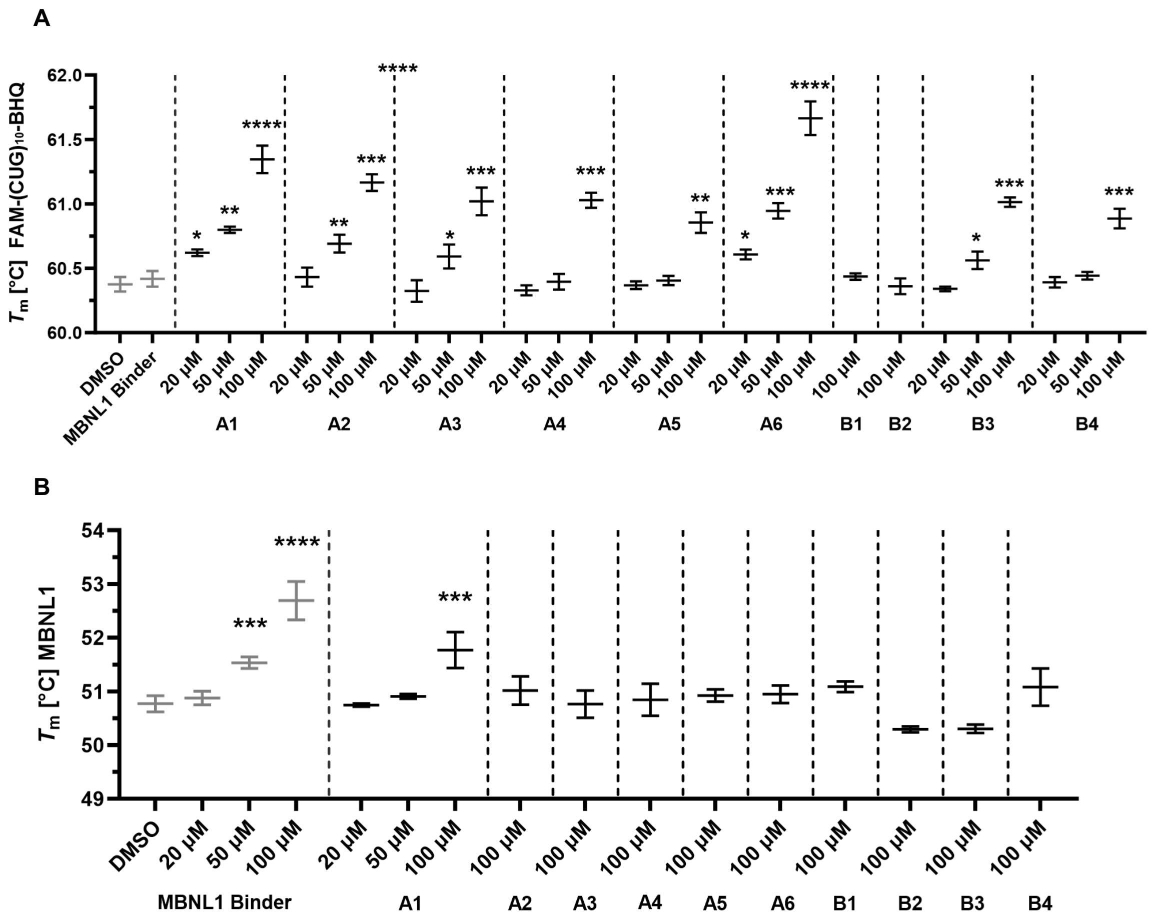

Figure S9. Differential scanning fluorimetry (DSF) analysis of compound engagement with FAM-$(CUG)_{10}$-BHQ or MBNL1 protein.

(A) Melting temperatures (T_ms) for r(CUG) repeats in the presence and absence of various small molecules, as obtained from the negative first derivative graph of a DSF melting profile from FAM-$(CUG)_{10}$-BHQ. The RNA was incubated with varying concentrations of compounds identified in these studies (100 µM, 50 µM and 20 µM) or with 100 µM of an MBNL1 binder (**Figure S3B**), which was used as a negative control.

(B) Melting temperatures (T_ms) for MBNL1 in the presence and absence of various small molecules, as obtained from the negative first derivative graph of a DSF melting profile from MBNL1 protein with SYPRO Orange dye. MBNL1 was incubated with varying concentrations of compounds identified in these studies (100 µM, 50 µM and 20 µM) or with the MBNL1 binder (**Figure S3B**).

Data are reported as mean ± SD (n = 3 biological replicates). Statistical significance was determined relative to DMSO using a two-tailed unpaired Student's t-test with significance thresholds: *, $p < 0.05$; **, $p < 0.01$; ***, $p < 0.001$; and ****, $p < 0.0001$.

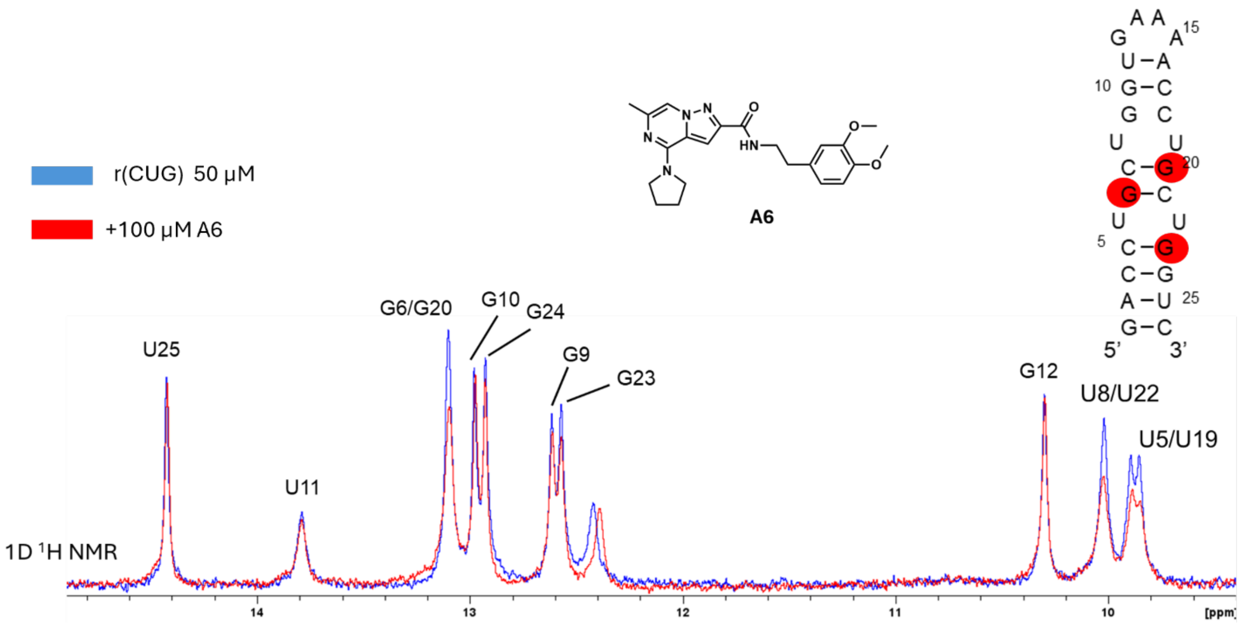

Figure S10. **1D imino proton spectra collected in the presence (red) and absence (blue) of A6 confirms that the small molecule binds the 1×1 nucleotide UU internal loop present in r(CUG)^{exp}.** Spectra were collected in 5 mM KH_2PO_4/K_2HPO_4, pH 6.0, and 50 mM NaCl using 50 µM of RNA in the presence and absence of 100 µM of **A6**.

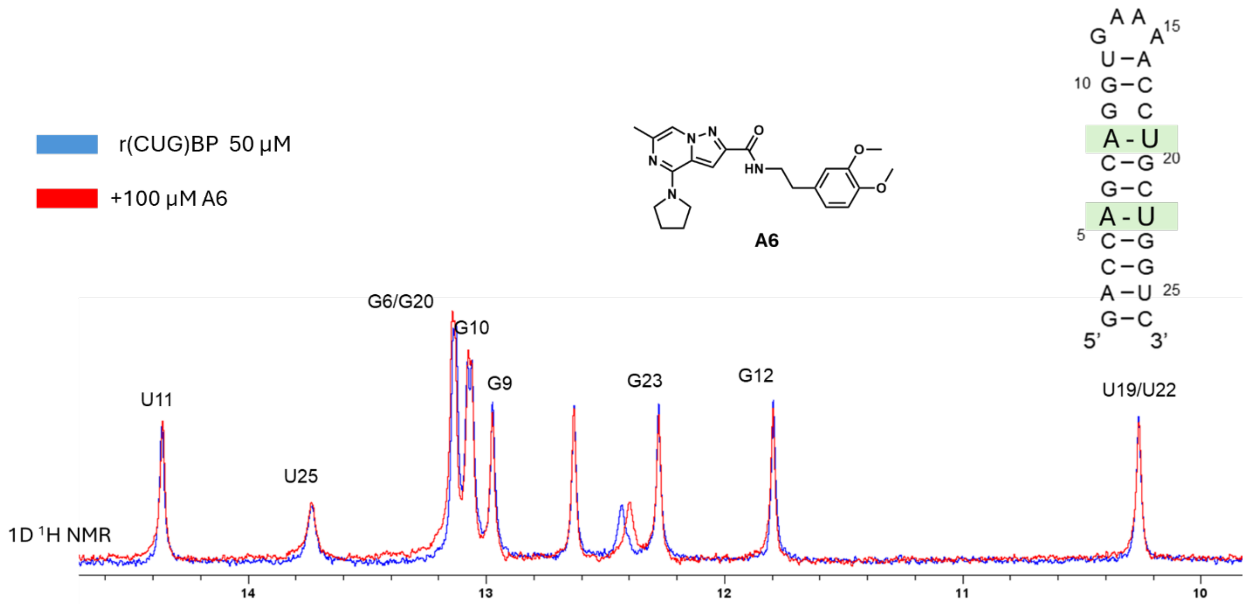

Figure S11. **1D imino proton spectra collected in the presence (red) and absence (blue) of A6 confirms that the small molecule does not bind to the base pair RNA.** Spectra were collected in 5 mM KH_2PO_4/K_2HPO_4, pH 6.0, and 50 mM NaCl using 50 µM of RNA in the presence and absence of 100µM of **A6**.

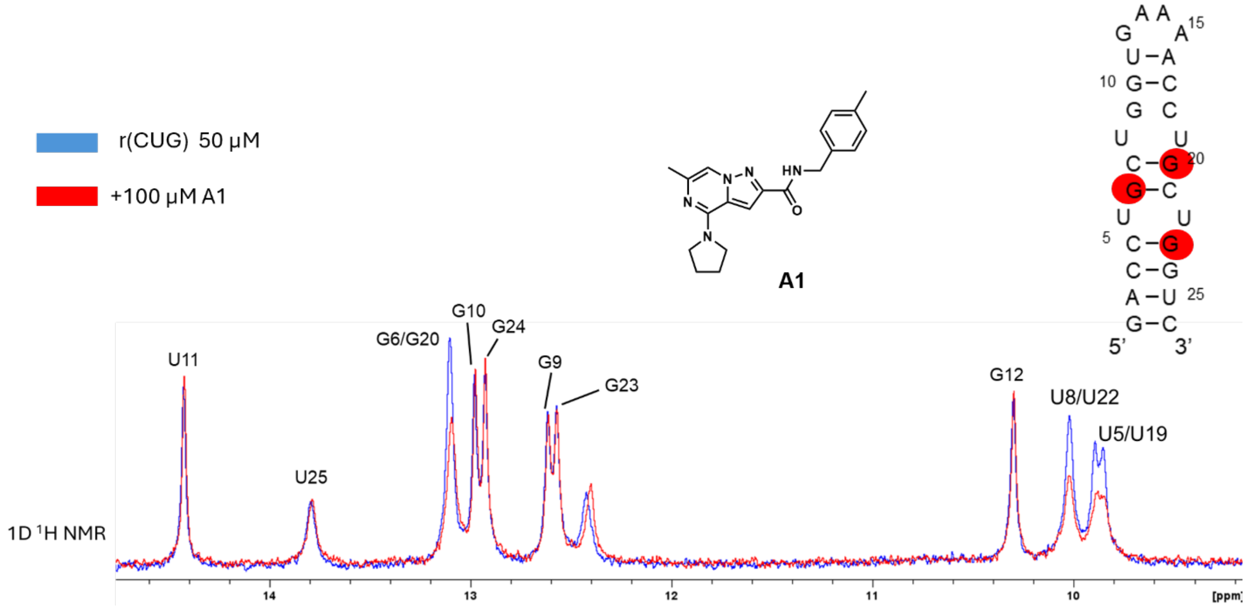

Figure S12. **1D imino proton spectra collected in the presence (red) and absence (blue) of A1 confirms that the small molecule binds the 1×1 nucleotide UU internal loop present in r(CUG)^{exp}.** Spectra were collected in 5 mM KH_2PO_4/K_2HPO_4, pH 6.0, and 50 mM NaCl using 50 mM of RNA in the presence and absence of 100 µM of **A1**.

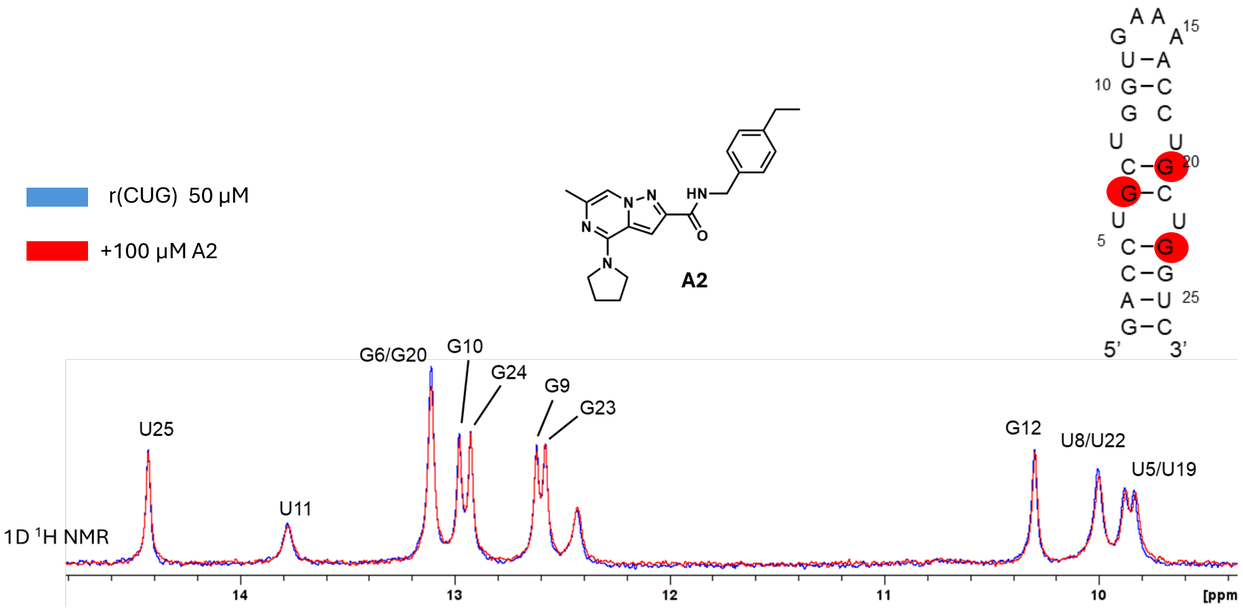

Figure S13. **1D imino proton spectra collected in the presence (red) and absence (blue) of A2 confirms that the small molecule binds the 1×1 nucleotide UU internal loop present in r(CUG)^{exp}.** Spectra were collected in 5 mM KH_2PO_4/K_2HPO_4, pH 6.0, and 50 mM NaCl using 50 mM of RNA in the presence and absence of 100 μM of **A2**.

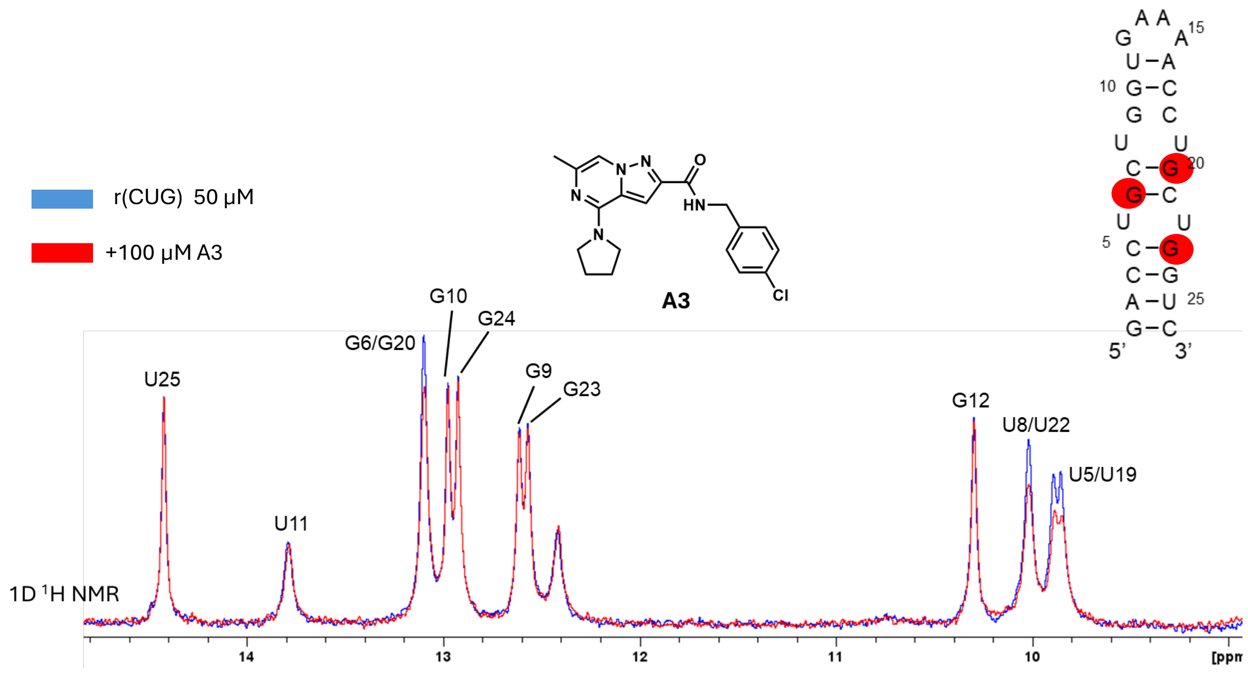

Figure S14. **1D imino proton spectra collected in the presence (red) and absence (blue) of A3 confirms that the small molecule binds the 1×1 nucleotide UU internal loop present in r(CUG)^{exp}.** Spectra were collected in 5 mM KH_2PO_4/K_2HPO_4, pH 6.0, and 50 mM NaCl using 50 mM of RNA in the presence and absence of 100 μM of **A3**.

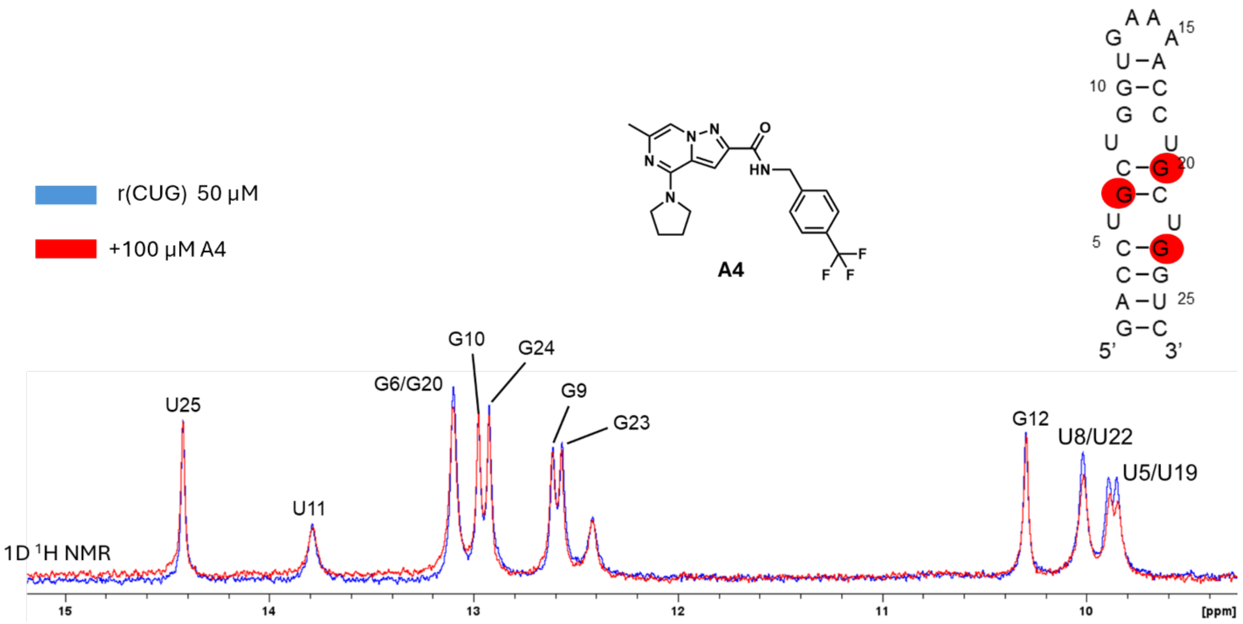

Figure S15. **1D imino proton spectra collected in the presence (red) and absence (blue) of A4 confirms that the small molecule binds the 1×1 nucleotide UU internal loop present in r(CUG)^{exp}.** Spectra were collected in 5 mM KH_2PO_4/K_2HPO_4, pH 6.0, and 50 mM NaCl using 50 mM of RNA in the presence and absence of 100 µM of **A4**.

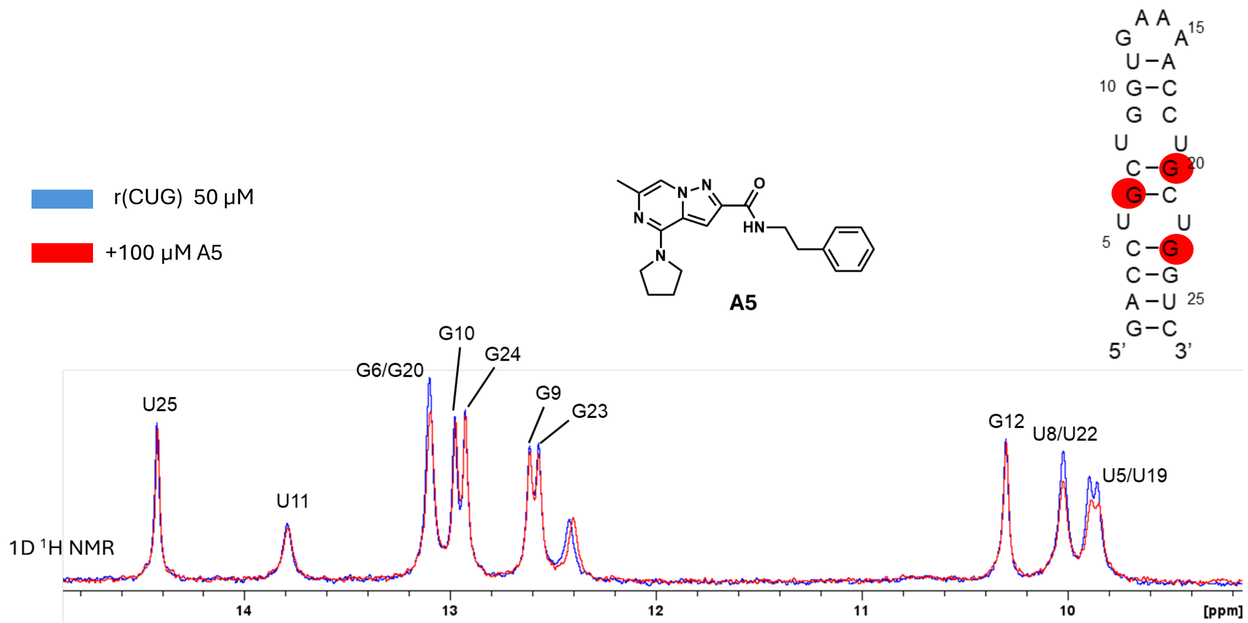

Figure S16. **1D imino proton spectra collected in the presence (red) and absence (blue) of A5 confirms that the small molecule binds the 1×1 nucleotide UU internal loop present in r(CUG)^{exp}.** Spectra were collected in 5 mM KH_2PO_4/K_2HPO_4, pH 6.0, and 50 mM NaCl using 50 mM of RNA in the presence and absence of 100 μM of **A5**.

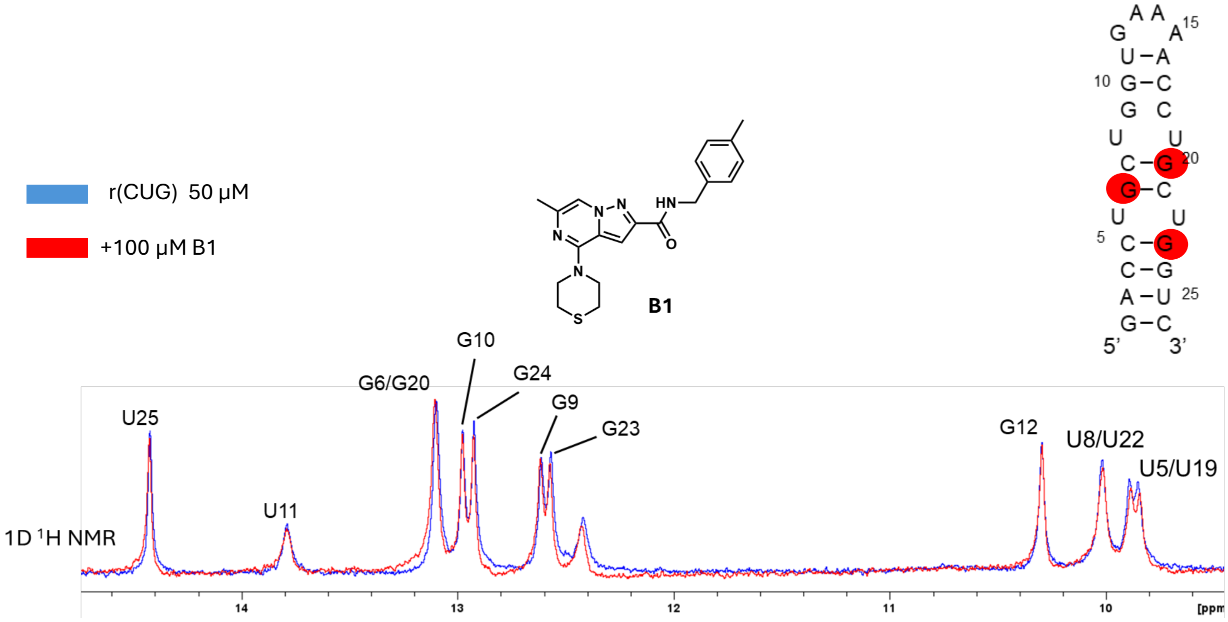

Figure S17. **1D imino proton spectra collected in the presence (red) and absence (blue) of B1 confirms that the small molecule binds the 1×1 nucleotide UU internal loop present in r(CUG)^{exp}.** Spectra were collected in 5 mM KH_2PO_4/K_2HPO_4, pH 6.0, and 50 mM NaCl using 50 mM of RNA in the presence and absence of 100 μM of **B1**.

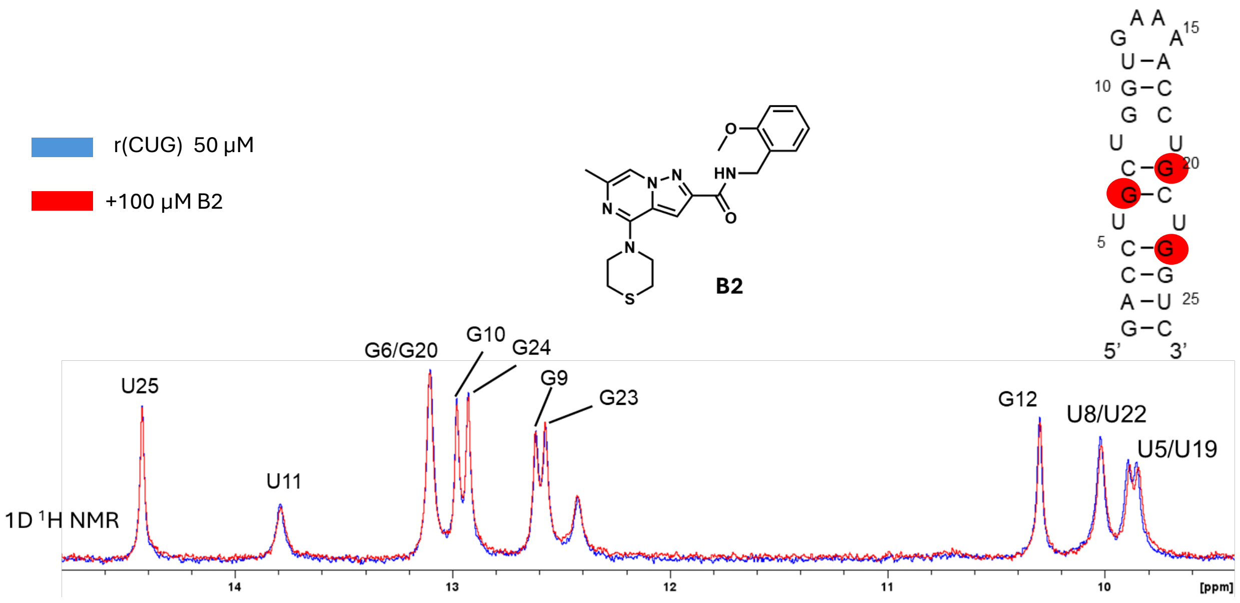

Figure S18. **1D imino proton spectra collected in the presence (red) and absence (blue) of B2 confirms that the small molecule binds the 1×1 nucleotide UU internal loop present in r(CUG)^{exp}.** Spectra were collected in 5 mM KH_2PO_4/K_2HPO_4, pH 6.0, and 50 mM NaCl using 50 mM of RNA in the presence and absence of 100 μM of **B2**.

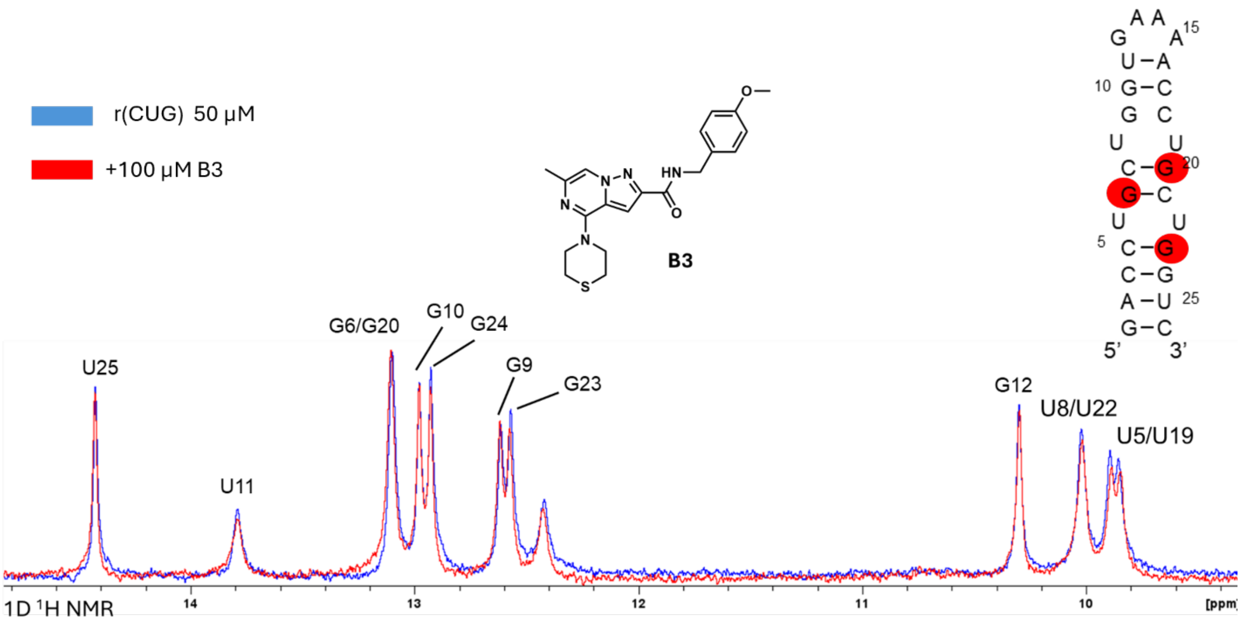

Figure S19. **1D imino proton spectra collected in the presence (red) and absence (blue) of B3 confirms that the small molecule binds the 1×1 nucleotide UU internal loop present in r(CUG)^{exp}.** Spectra were collected in 5 mM KH_2PO_4/K_2HPO_4, pH 6.0, and 50 mM NaCl using 50 mM of RNA in the presence and absence of 100 μM of **B3**.

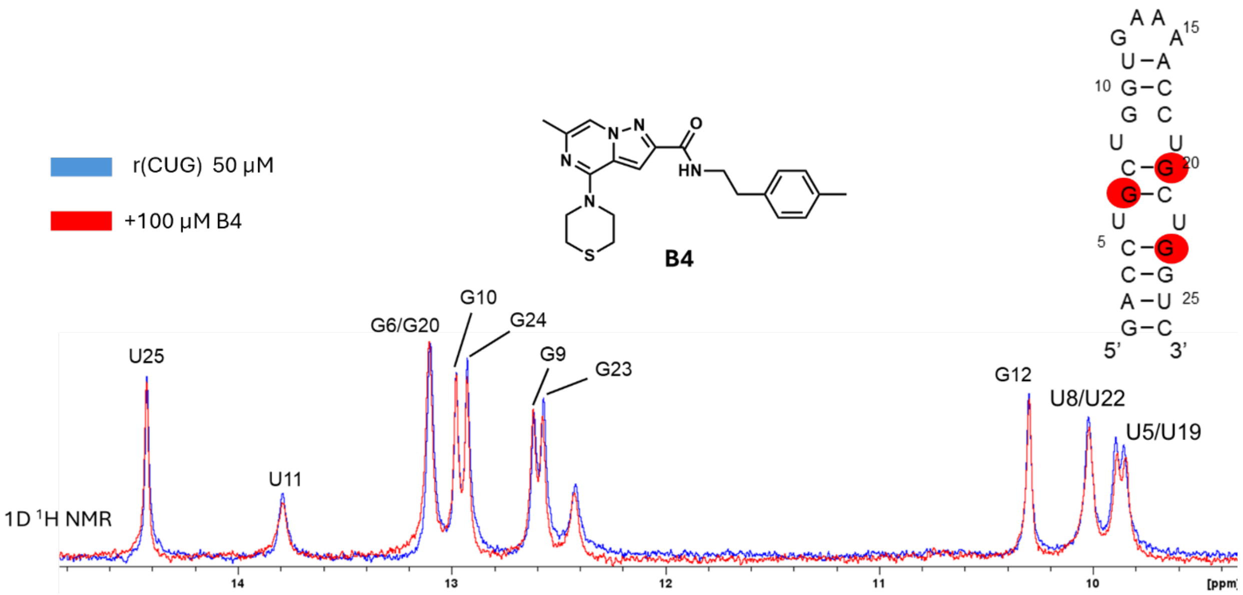

Figure S20. **1D imino proton spectra collected in the presence (red) and absence (blue) of B4 confirms that the small molecule binds the 1×1 nucleotide UU internal loop present in r(CUG)^{exp}.** Spectra were collected in 5 mM KH_2PO_4/K_2HPO_4, pH 6.0, and 50 mM NaCl using 50 mM of RNA in the presence and absence of 100 µM of **B4**.

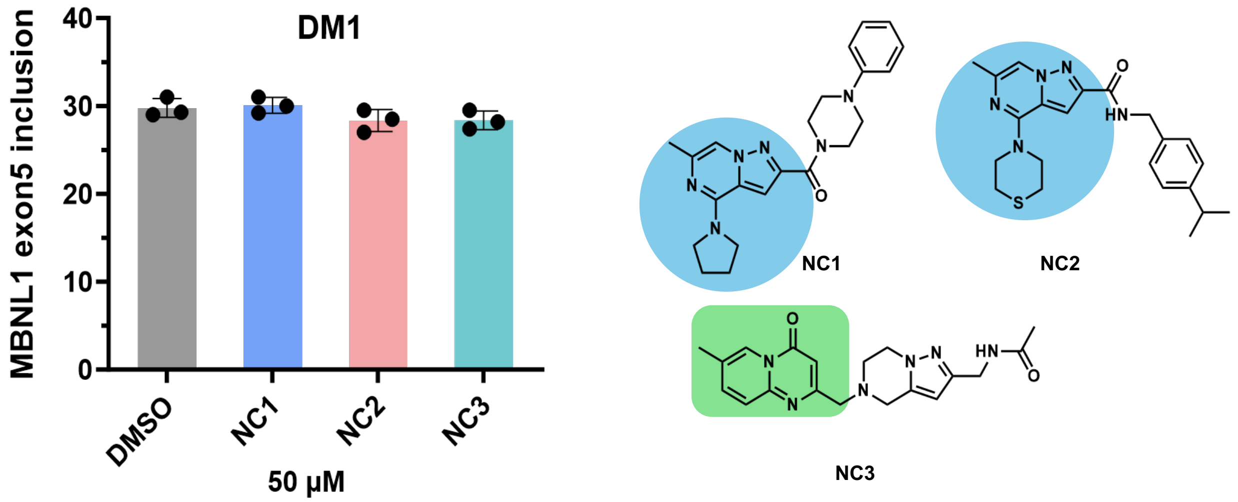

Figure S21. Evaluation of DM1 patient-derived myotubes treated with 50 μM negative control compounds.
Effect of three negative control compounds (50 μM) with distinct chemical cores that showed no activity in the NanoBRET assay on *MBNL1* exon 5 alternative splicing in DM1 patient-derived myotubes (n = 3 biological replicates).

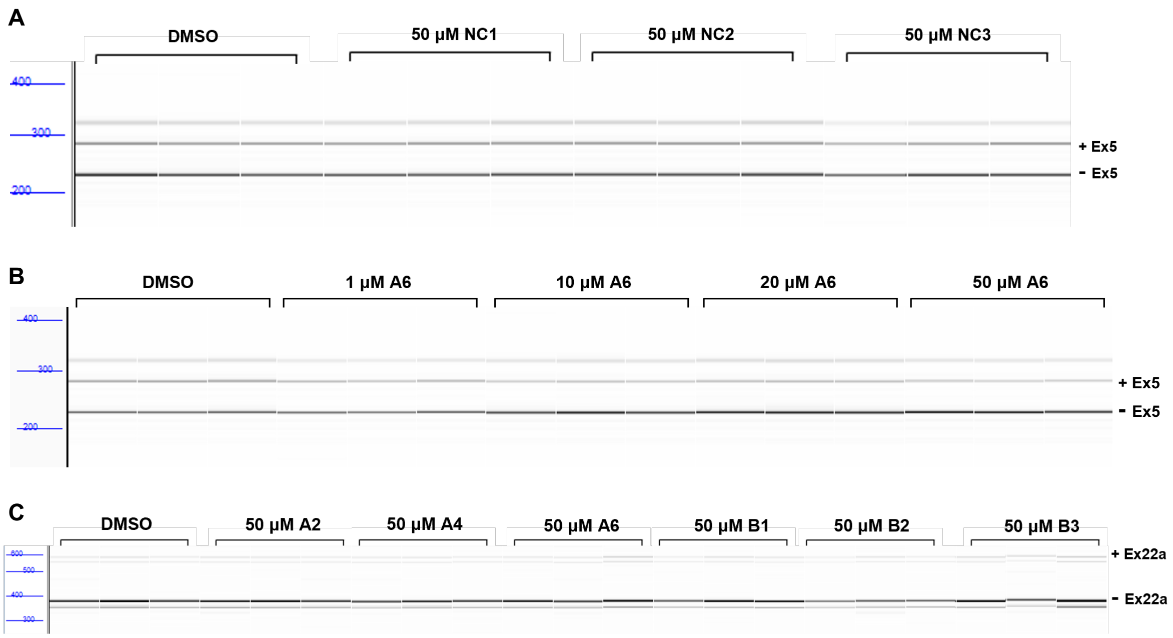

Figure S22. Fragment analyzer gel of *MBNL1* exon 5 and *MAP4K4* exon 22a splicing event in DM1 patient-derived myotubes

(A) Effect of three negative control compounds on MBNL1 exon 5 splicing, as assessed by end-point RT-PCR and fragment analyzer. Patient-derived myotubes were treated with 50 µM of **NC1**, **NC2** and **NC3**. Exon 5 inclusion is indicated by "+ Ex5" while exclusion is indicated by "-Ex5" (n = 3 biological replicates).

(B) Effect of **A6** on MBNL1 exon 5 splicing, as assessed by end-point RT-PCR and fragment analyzer. DM1 patient-derived myotubes were treated with 1, 10, 20, 50 µM of **A6**. Exon 5 inclusion is indicated by "+ Ex5" while exclusion is indicated by "-Ex5" (n = 3 biological replicates).

(C) Effect of six compounds on NOVA-regulated *MAP4K4* exon 22a splicing at 50 µM, as assessed by end-point RT-PCR and fragment analyzer. Exon 22a inclusion is indicated by "+ Ex22a" while exclusion is indicated by "-Ex22a" (n = 3 biological replicates).

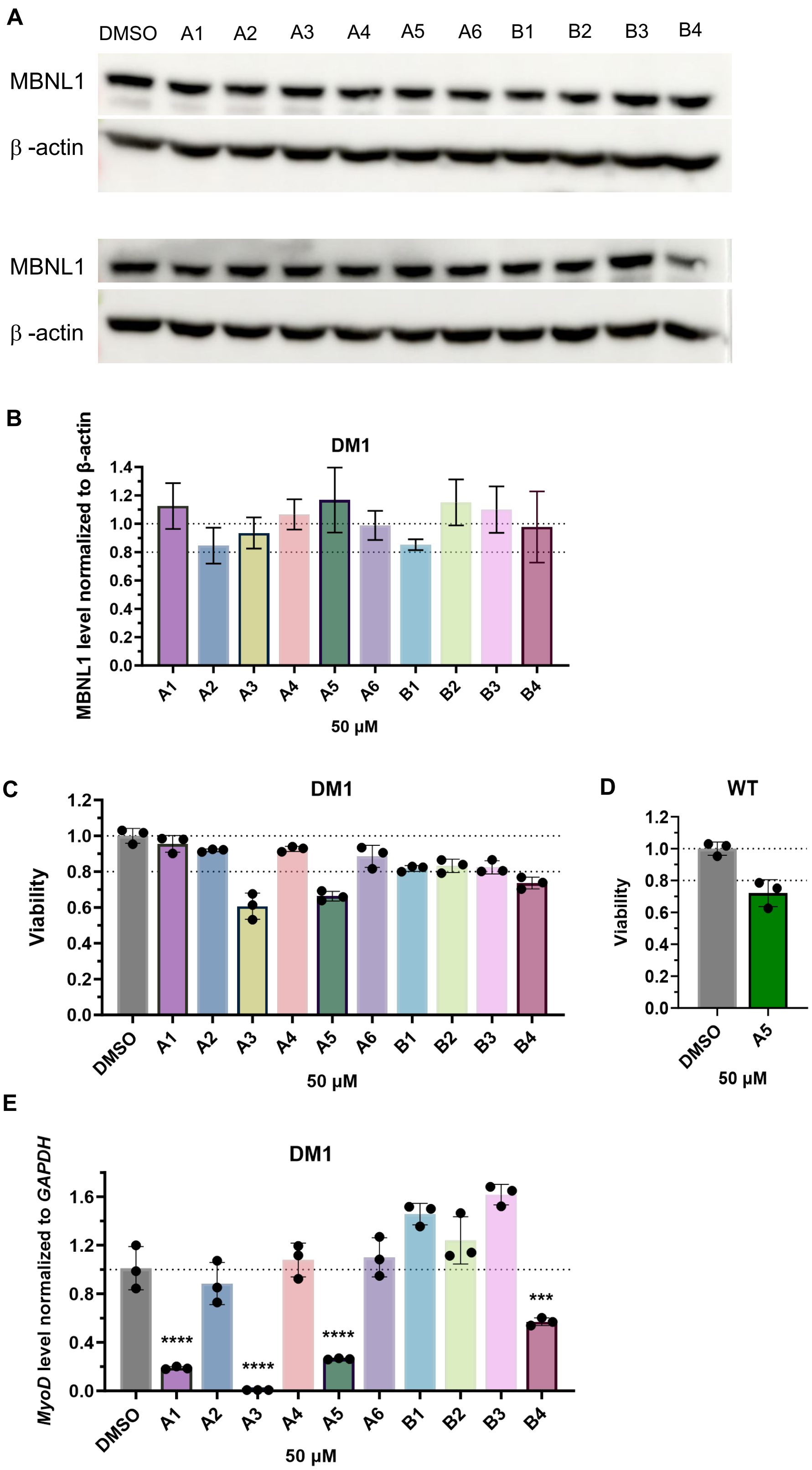

Figure S23. Evaluation of *MyoD* transcript and MBNL1 protein levels in DM1 patient-derived and WT myotubes upon treatment with 50 µM of various compounds.

(A) Effect of various compounds on MBNL1 expressio*n* levels upon treatment of DM1 patient-derived myotubes determined by Western blot (n = 2 biological replicates).

(B) Quantification of MBNL1 band intensities (n = 2) from panel (A). Signals were first normalized to the corresponding β-actin and then expressed relative to the DMSO-treated group (set to 1). Data are reported as mean ± standard deviation.

(C) Viability of DM1 patient-derived myotubes following treatment with 50 μM of the indicated small molecules. Viability was measured using a luminescence-based ATP assay and normalized to the DMSO-treated cells. Data are reported as mean ± standard deviation (n = 3 biological replicates).

(D) Viability of WT myotubes upon treatment with 50 μM of the **A5**. Data are reported as mean ± standard deviation (n = 3 biological replicates).

(E) Effect of various compounds on *MyoD* transcript levels upon treatment of DM1 patient-derived myotubes, as determined by RT-qPCR (normalized to *GAPDH*; n = 3 biological replicates).
Statistical significance was determined relative to DMSO-treated samples using a two-tailed unpaired Student's t-test with significance thresholds: *, $p < 0.05$; **, $p < 0.01$; ***, $p < 0.001$; and ****, $p < 0.0001$.

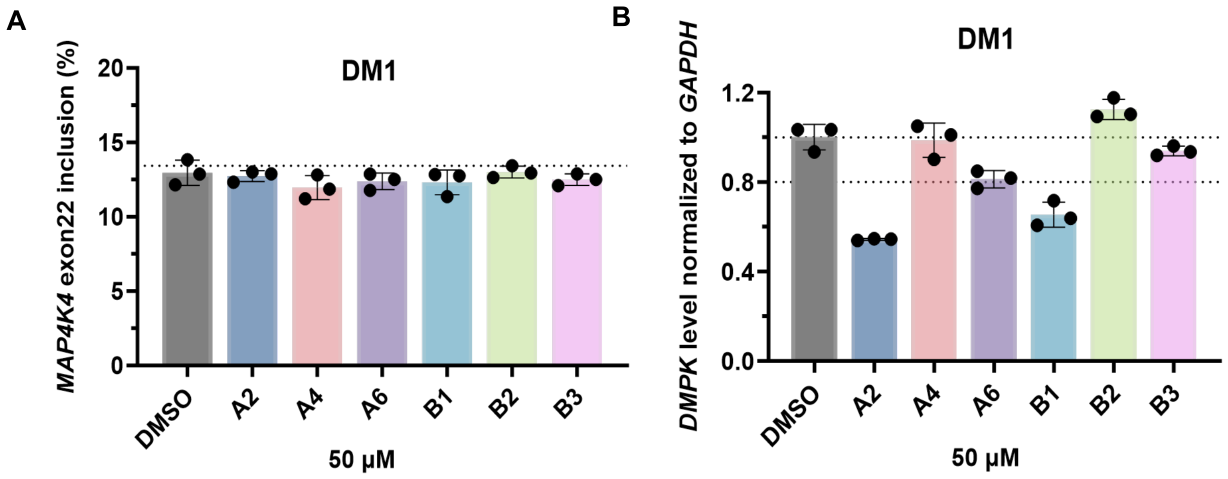

Figure S24. Evaluation of a non-MBNL1-regulated splicing event, *DMPK* transcript levels, in DM1 patient-derived myotubes treated with 50 μM of compound.

(A) Effect of the six compounds that improve DM1-associated alternative splicing events on NOVA-regulated *MAP4K4* exon 22a splicing (n = 3 biological replicates).

(B) Effect of various compound on *DMPK* transcript levels upon treatment of DM1 patient-derived myotubes, as determined by RT-qPCR (normalized to *GAPDH*; n = 3 biological replicates). Compounds **A2** and **B1** reduced *DMPK* abundance by > 20%, suggesting they might inhibit transcription, while the others had no effect.

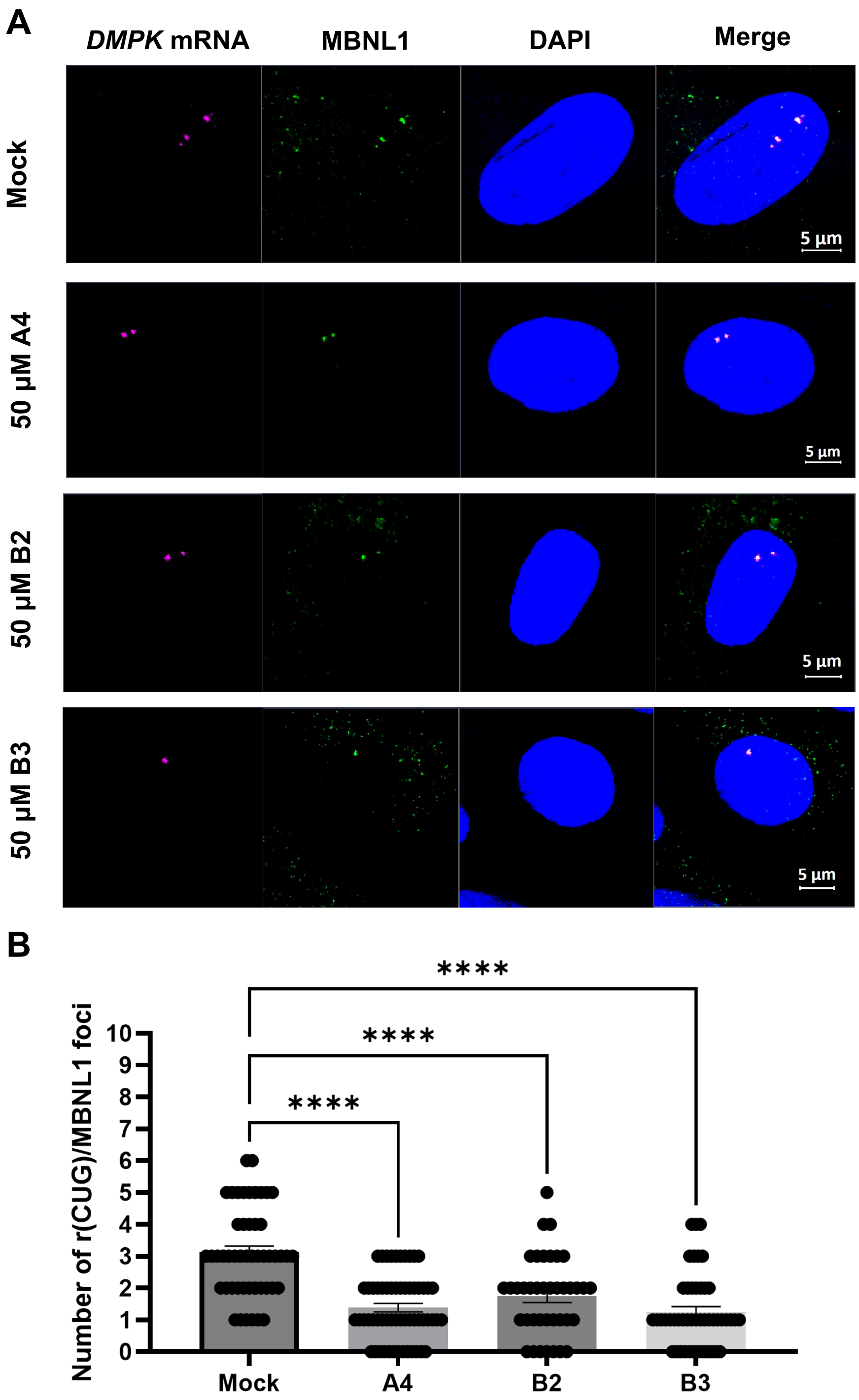

Figure S25: Effect of A4, B2, and B3 on the formation of nuclear foci in DM1 patient-derived myotubes.

(A) Representative microscopic images of r(CUG)^{exp}–MBNL1 foci imaged by RNA fluorescence in situ hybridization (FISH) and anti-MBNL1 immunostaining upon treatment of DM1 patient-derived myotubes with 50 μM compound or vehicle (0.1% (v/v) DMSO). Endogenous MBNL1 was

detected using an anti-MBNL1 antibody (green), and r(CUG)^{exp} was imaged using smFISH probes targeting *DMPK* CDS. DAPI staining (blue) was used to visualize nuclei. Scale bar is 5 μM.

(B) Quantification of r(CUG)$^{exp-}$containing foci in the nucleus of DM1 myotubes (with 40 nuclei quantified/replicate; n = 3 biological replicates). *, $p < 0.05$; and ****, $p < 0.0001$, as determined by Student's t-test. Data are reported as the mean ± SEM.

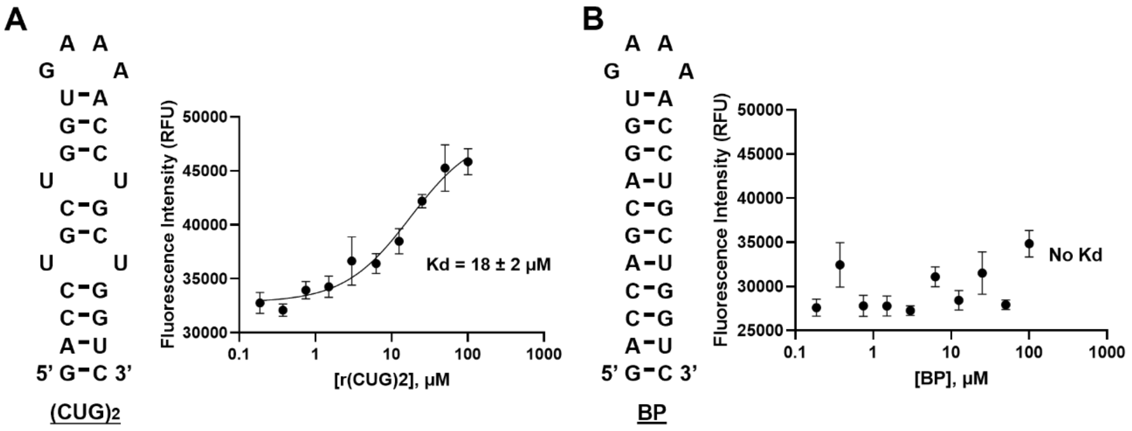

Figure S26: Affinity of A6 for a r(CUG) repeat and a fully paired RNA using a fluorescence-based binding assay

(A) Secondary structures of the RNA used in binding assays and the change in fluorescence of **A6** (100 nM) as a function of RNA concentration, yielding a dissociation constant (K_d) of 17 ± 2 μM.

(B) Titration of the control RNA (BP) with **A6** (100 nM) did not produce a significant change in fluorescence, suggesting no measurable binding affinity.

All measurements were conducted in 1× TR-FRET Assay Buffer lacking Tween-20 (20 mM HEPES, pH 7.5, 110 mM KCl,10 mM NaCl, 2 mM $MgCl_2$, 2 mM $CaCl_2$, 5 mM dithiothreitol (DTT), and 0.1% (w/v) BSA). Data are reported as mean ± standard deviation (n = 3 independent measurements).

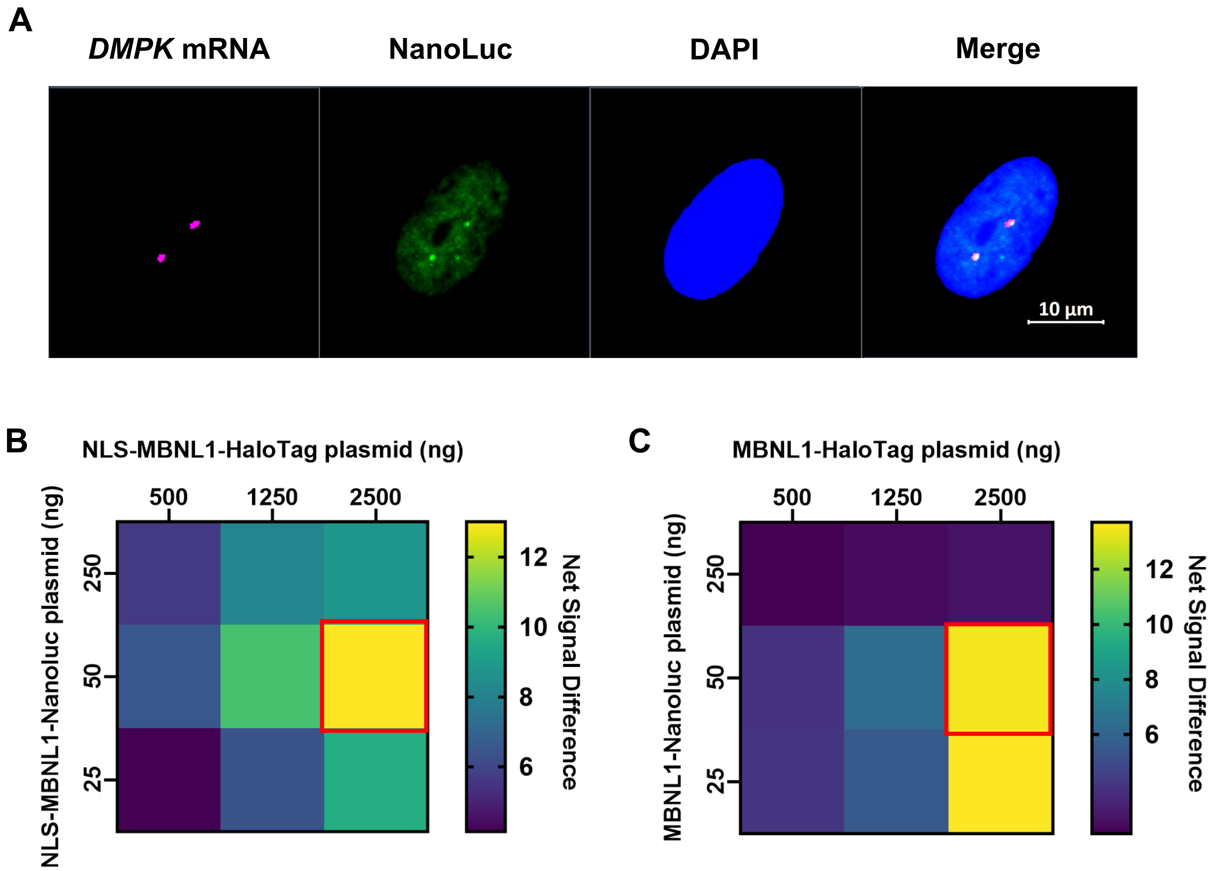

Figure S27 Heat-map of NanoBRET window sizes across NLS-MBNL1 plasmid ratios and MBNL1 plasmid ratios.

(A) IFA for NanoLuc (green) and smRNA-FISH for DMPK mRNA (magenta). Representative images showing the colocalization of NLS-MBNL1-NanoLuc with $r(CUG)^{exp}$ in HeLa480 cells. NLS-MBNL1-NanoLuc was detected using an anti-NanoLuc antibody (green), and $r(CUG)^{exp}$ was imaged using smFISH probes targeting DMPK exons 11 - 15. DAPI staining (blue) was used to visualize nuclei. Scale bar is 10 μM.

(B) Heat maps summarizing assay window sizes—defined as the decrease in NanoBRET signal after treatment with 10 μM CAG25 VivoMorpholino—obtained at the indicated DNA ratios of NLS-MBNL1-HaloTag to NLS-MBNL1-NanoLuc. The optimal configuration (50 ng NLS-MBNL1-NanoLuc : 2500 ng NLS-MBNL1-HaloTag) produced the largest window (~13 mBU; highlighted in red).

(C) Heat maps summarizing assay window sizes—defined as the decrease in NanoBRET signal after treatment with 10 μM CAG25 VivoMorpholino—obtained at the indicated DNA ratios of MBNL1-HaloTag to MBNL1-NanoLuc. The optimal configuration (50 ng MBNL1-NanoLuc : 2500 ng MBNL1-HaloTag) produced the largest window (~13.5 mBU; highlighted in red).

COMPOUND CHARACTERIZATION

General

NMR spectra were acquired by a 400 UltraShieldTM (Bruker) (400 MHz for ^{1}H) or an AscendTM 600 (Bruker) (150 MHz for ^{13}C). Chemical shifts are expressed in ppm relative to trimethyl silane (TMS) for ^{1}H and residual solvent for ^{13}C as internal standards. Coupling constants (J values) are reported in Hz. High resolution mass spectra were recorded on 4800 Plus MALDI TOF/TOF Analyzer (Applied Biosystems) using the α-cyano-4- hydroxycinnamic acid matrix. Purities of products were analyzed by HPLC (Agilent 1290 Infinity) using a SunFire® C18 3.5 μm column (4.6x150 mm) with a flow rate of 1 mL/min.

Compound A6:

^{1}H NMR (400 MHz, $CDCl_3$): δ= 7.44 (s, 1H), 7.30 (d, J = 0.92, 1H), 7.09 (t, J = 5.9 Hz, 1H), 6.85-6.76 (m, 3H), 3.88 (s, 3H), 3.86 (s, 3H), 3.89-3.80 (m, 4H), 3.70 (q, J = 6.8 Hz, 2H), 2.89 (t, J = 6.8 Hz, 2H), 2.29 (d, J = 0.88 Hz, 3H), 2.07-2.01 (m, 4H); ^{13}C NMR (150 MHz, $CDCl_3$):161.92, 149.90, 148.98, 147.67, 145.11, 139.41, 131.51, 127.57, 120.68, 112.07, 111.42, 108.71, 101.05, 55.93, 55.82, 48.58, 40.65, 35.55, 25.42, 21.28 ;δ= HR-MS (MALDI): Calcd. for C22H28N5O3$^+$ [M+H]$^+$, 410.2187; found, 410.2167

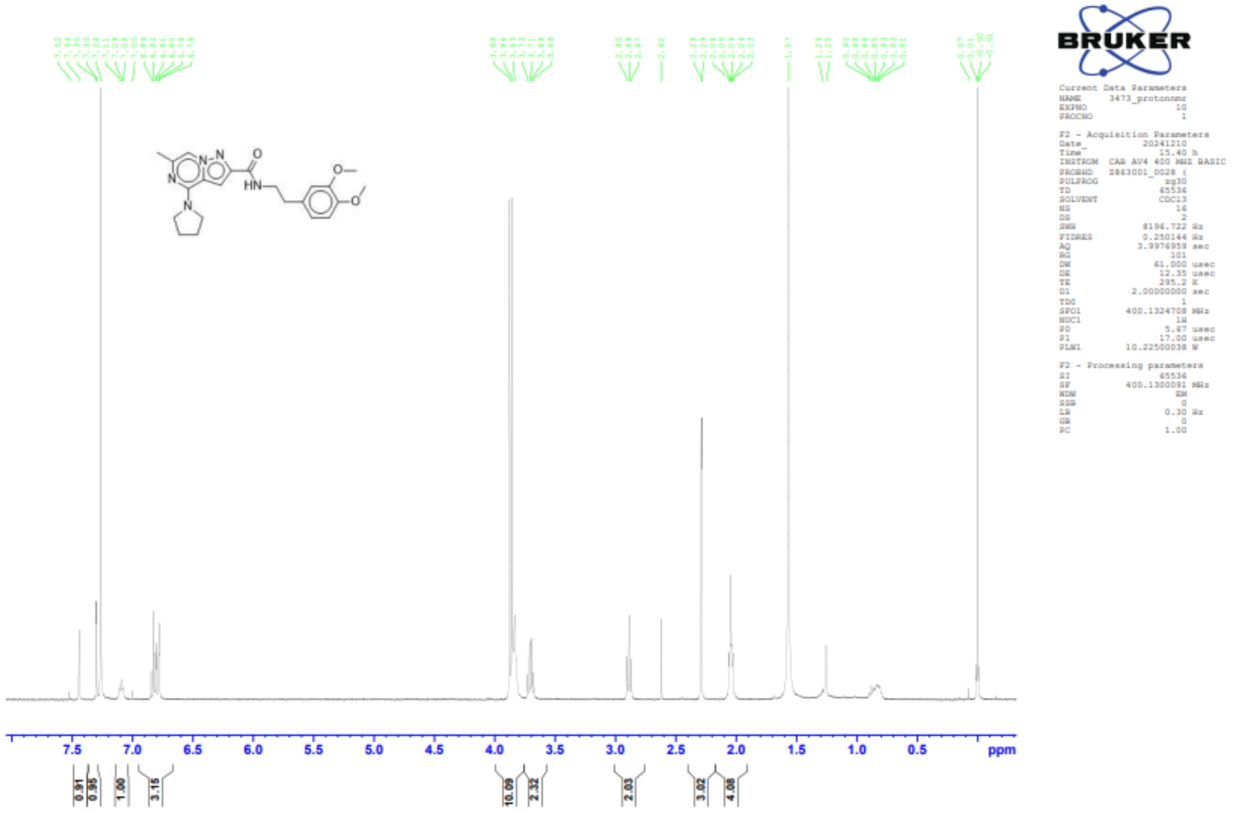

Figure S28. ^{1}H NMR spectrum of **A6.**

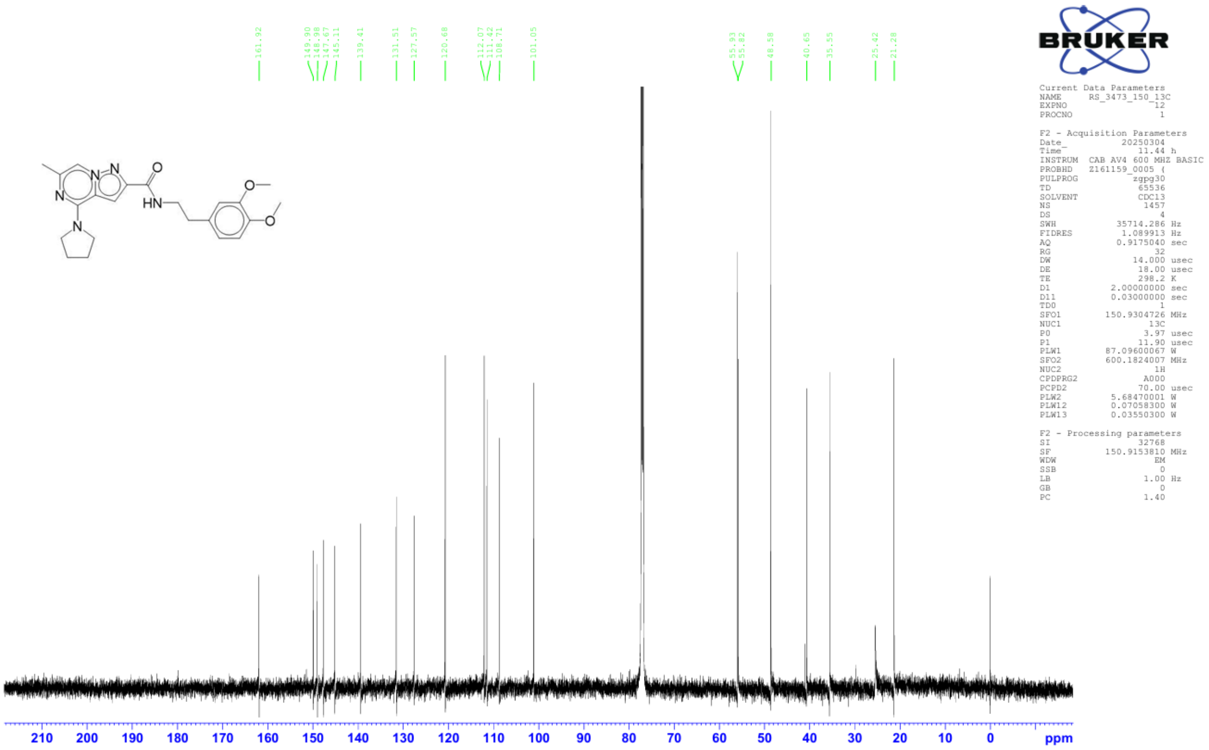

Figure S29. ^{13}C NMR spectrum of **A6**.

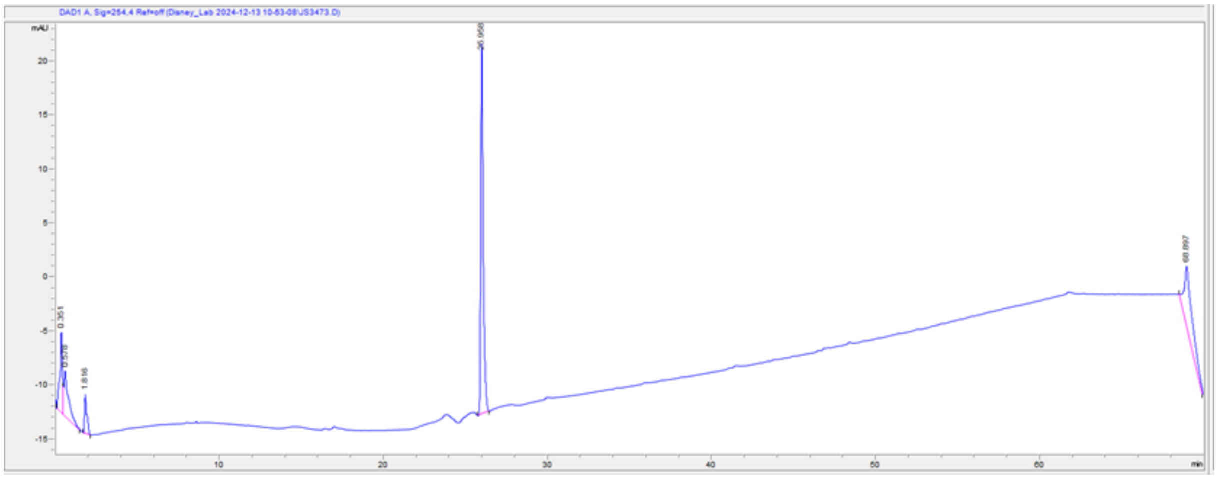

Figure S30. Analytical HPLC trace of **A6**: 0-100%/60 min MeOH/H2O (0.1% (v/v) TFA), where absorbance was measured at 254 nm.

Compound A1:

^{1}H NMR (400 MHz, $CDCl_3$): δ= 7.43 (s, 1H), 7.34 (s, 1H), 7.30-7.20 (m, 2H), 7.19-7.13 (m, 3H), 4.62 (d, *J* = 5.9 Hz, 2H), 3.88-3.81 (m, 4H), 2.34 (s, 3H), 2.29 (s, 3H), 2.08-2.02 (m, 4H); HR-MS (MALDI): Calcd. for $C_{20}H_{24}N_5O^+$ $[M+H]^+$, 350.1975; found, 350.1995

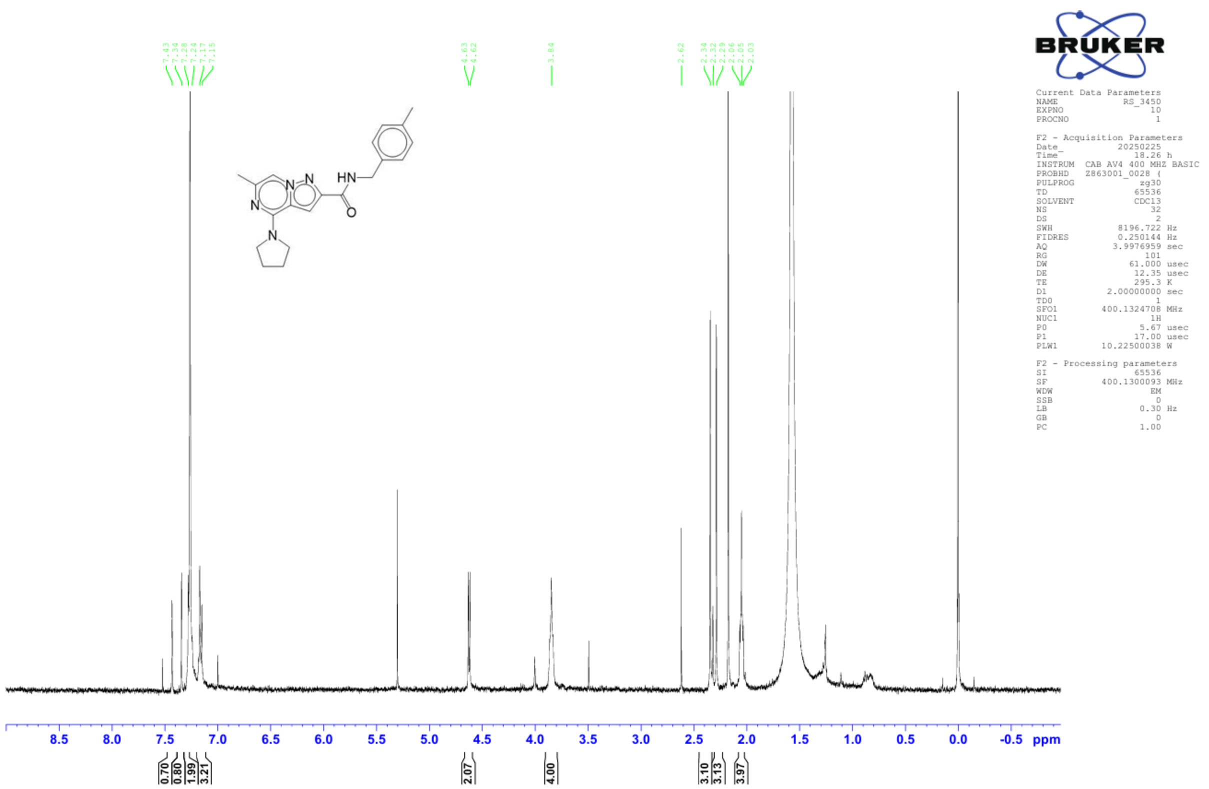

Figure S31. ^{1}H NMR spectrum of **A1**.

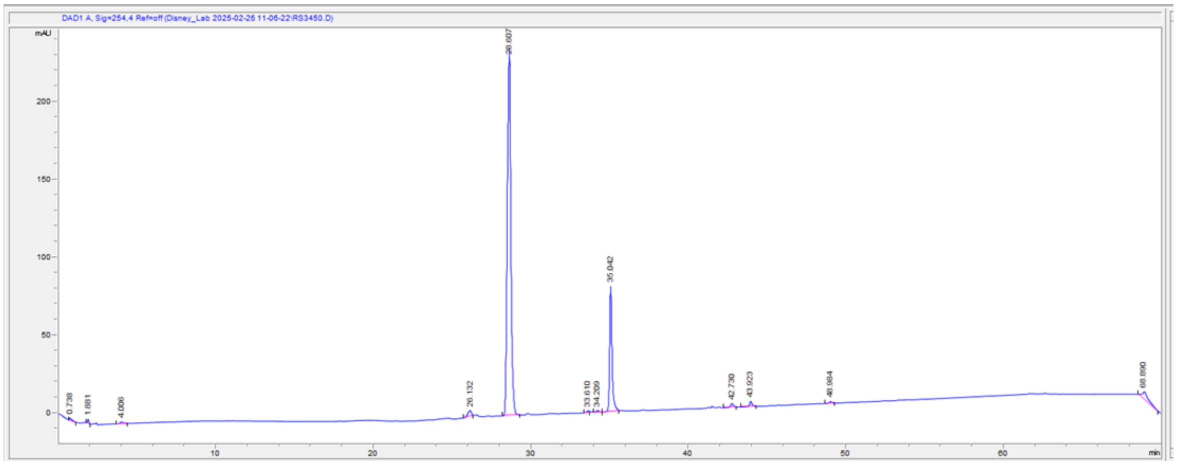

Figure S32. Analytical HPLC trace of **A1**: 0-100%/60 min MeOH/H2O (0.1% (v/v) TFA), where absorbance was measured at 254 nm.

Compound A2:
^{1}H NMR (400 MHz, $CDCl_3$): δ= 7.43 (s, 1H), 7.34 (d, *J* = 1.1 Hz, 1H), 7.30 (d, *J* = 8.2 Hz, 2H), 7.32-7.23 (m, 1H), 7.19 (d, *J* = 8.2 Hz, 2H), 4.63 (d, *J* = 5.9 Hz, 2H), 3.90-3.78 (m, 4H), 2.64 (q, *J* = 7.2 Hz, 2H), 2.28 (d, *J* = 1.0 Hz, 3H), 2.09-2.00 (m, 4H), 1.24 (t, *J* = 7.6 Hz, 3H); HR-MS (MALDI): Calcd. for $C_{21}H_{26}N_5O^+$ $[M+H]^+$, 364.2132; found, 364.2101

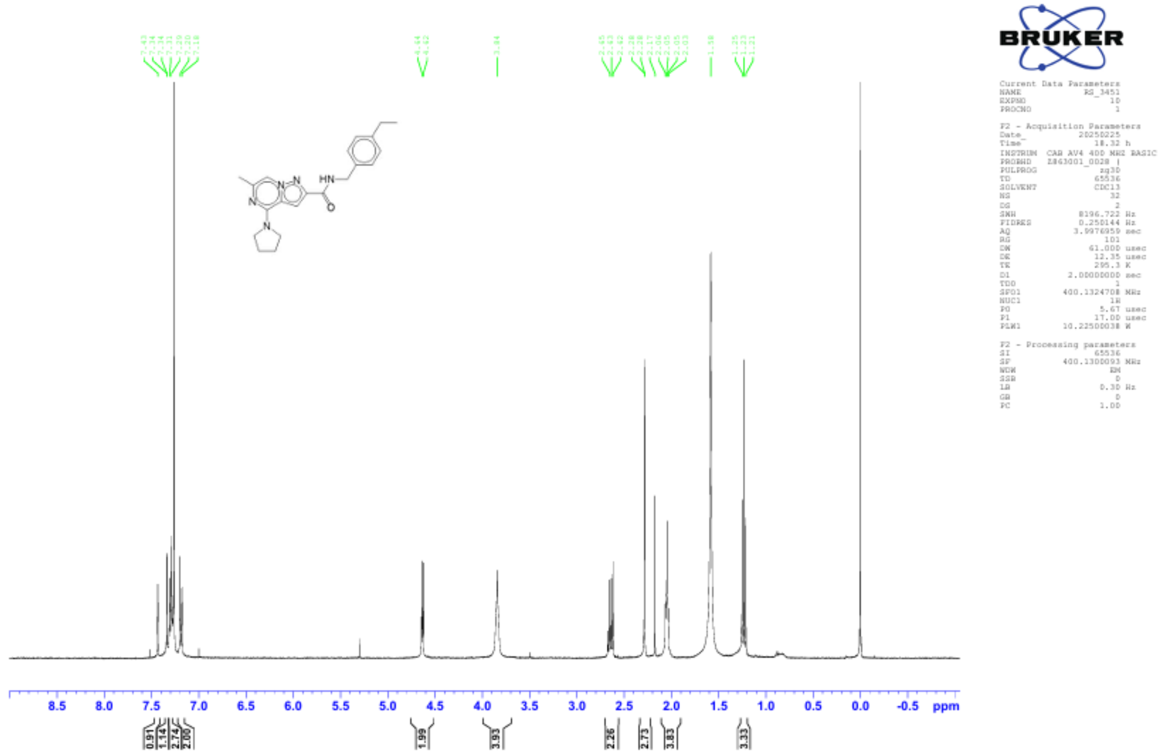

Figure S33. ^{1}H NMR spectrum of **A2**.

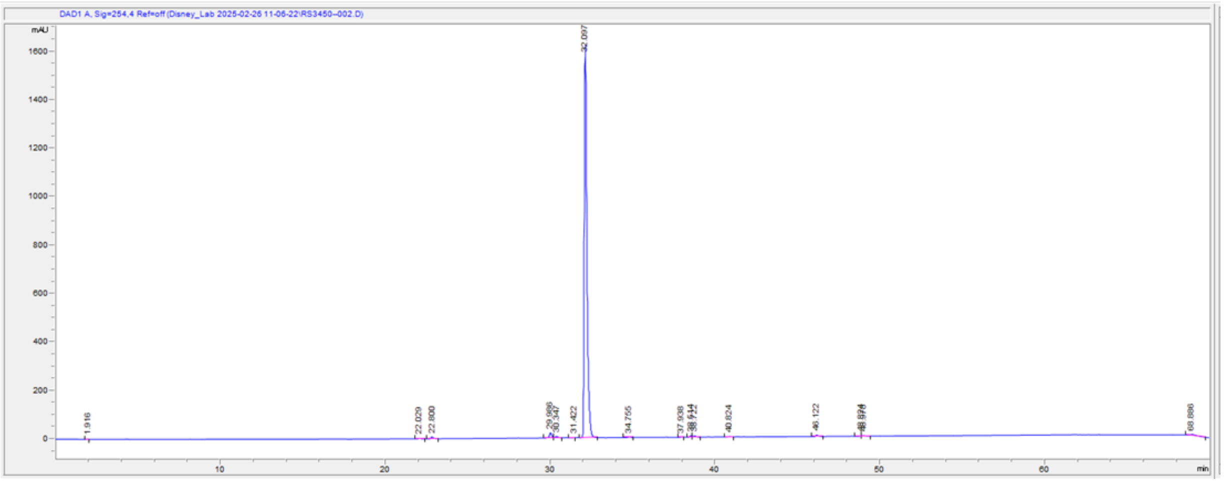

Figure S34. Analytical HPLC trace of **A2**: 0-100%/60 min MeOH/H2O (0.1% (v/v) TFA), where absorbance was measured at 254 nm.

Compound A3:

^{1}H NMR (400 MHz, $CDCl_3$): δ= 7.45 (s, 1H), 7.38-7.29 (m, 6H), 4.63 (d, *J* = 6.1 Hz, 2H), 3.88-3.80 (m, 4H), 2.29 (d, *J* = 1.0 Hz, 3H), 2.08-2.02 (m, 4H); HR-MS (MALDI): Calcd. for $C_{19}H_{21}ClN_5O^+$ $[M+H]^+$, 370.1429; found, 370.1451

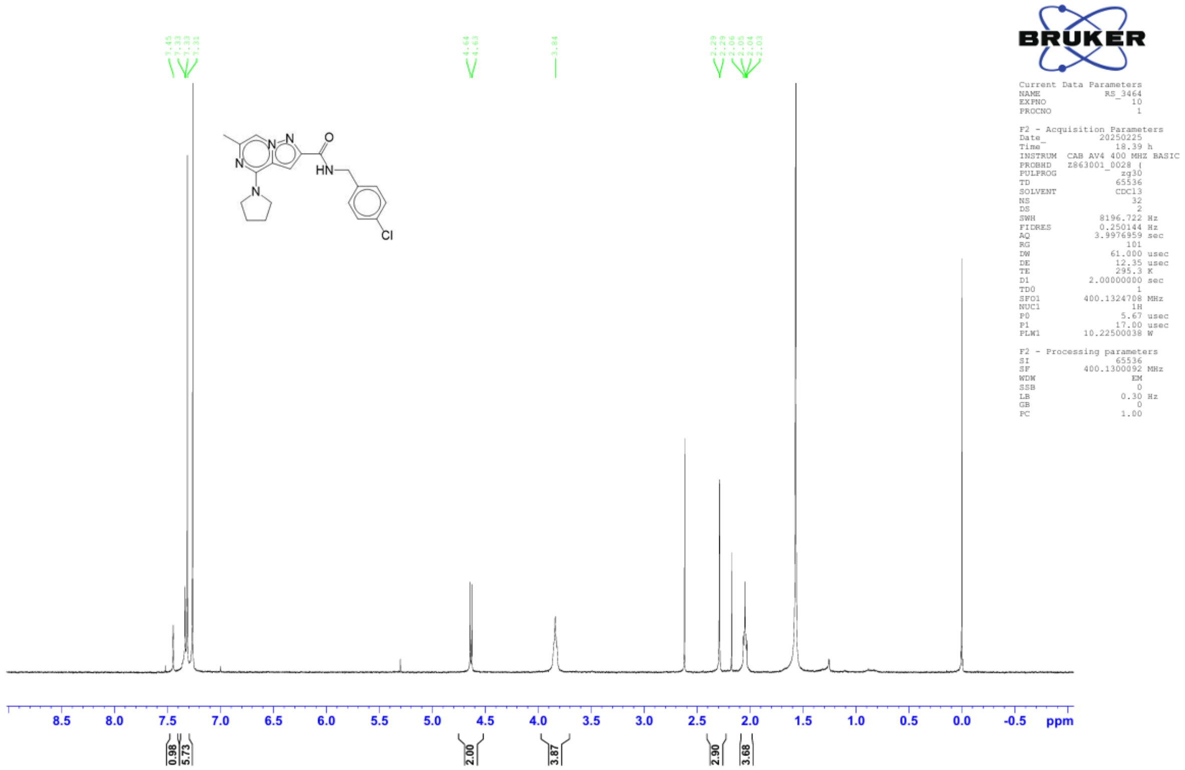

Figure S35. ^{1}H NMR spectrum of **A3**.

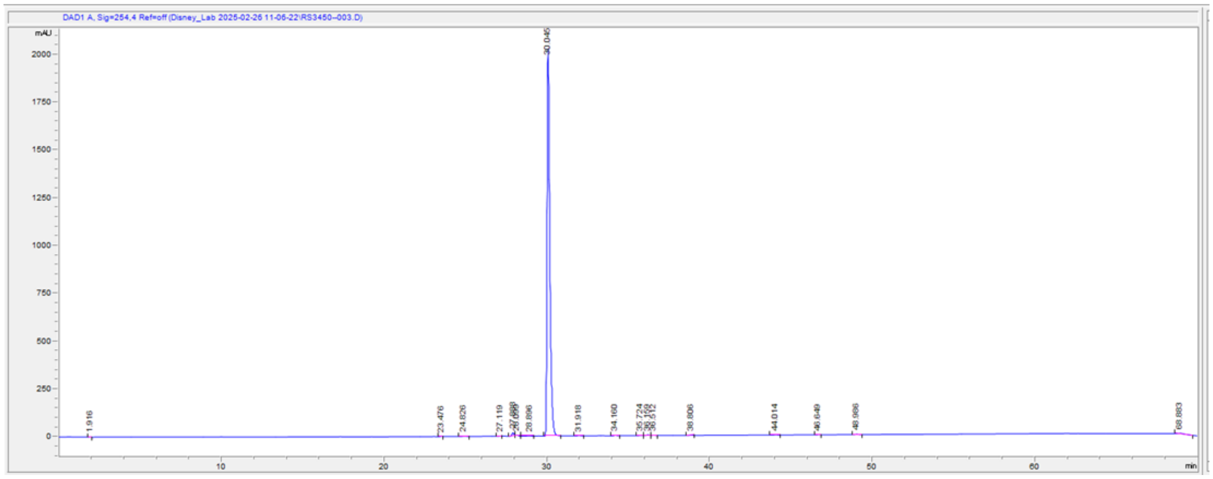

Figure S36. Analytical HPLC trace of **A3**: 0-100%/60 min MeOH/H2O (0.1% (v/v) TFA), where absorbance was measured at 254 nm.

Compound A4:

^{1}H NMR (400 MHz, $CDCl_3$): δ= 7.60 (d, *J* = 8.3 Hz, 2H), 7.49 (d, *J* = 8.3 Hz, 2H), 7.45 (s, 1H), 7.45-7.38 (m, 1H), 7.34 (s, 1H), 4.73 (d, *J* = 6.3 Hz, 2H), 3.88-3.79 (m, 4H), 2.29 (s, 3H), 2.08-2.02 (m, 4H); HR-MS (MALDI): Calcd. for $C_{20}H_{21}F_3N_5O^+$ $[M+H]^+$, 404.1693; found, 404.1660

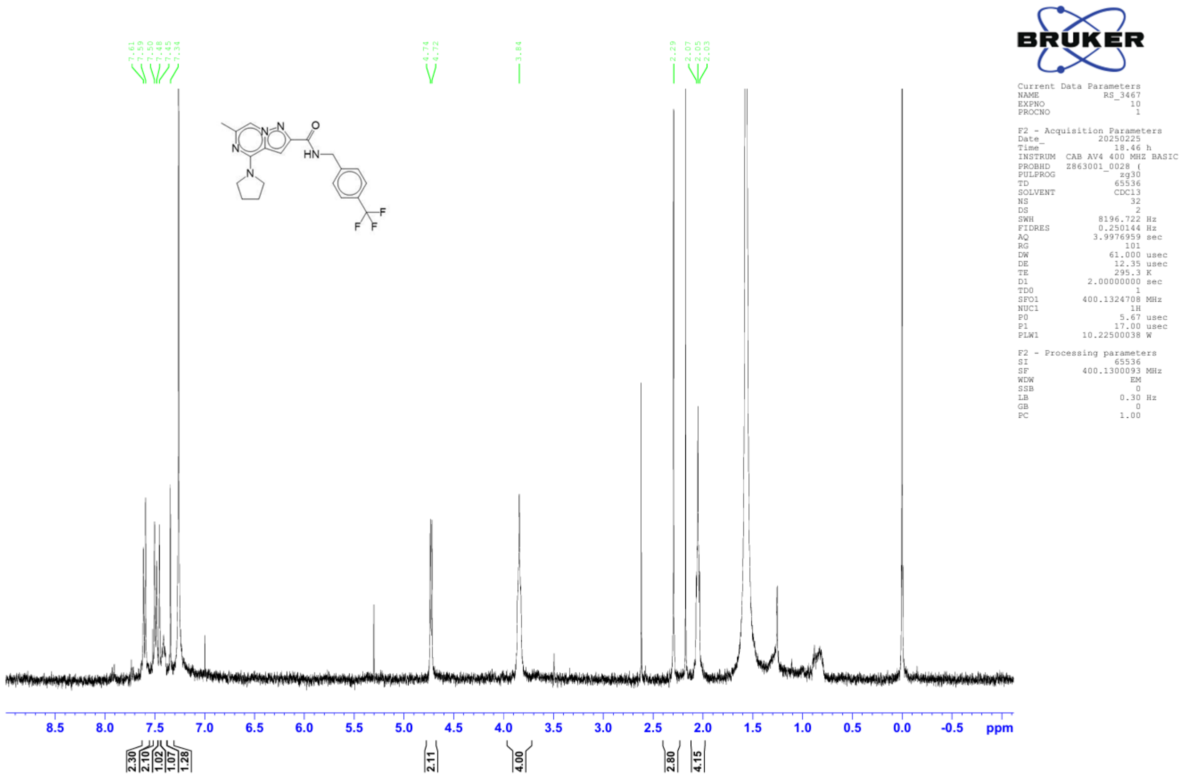

Figure S37. ^{1}H NMR spectrum of **A4**.

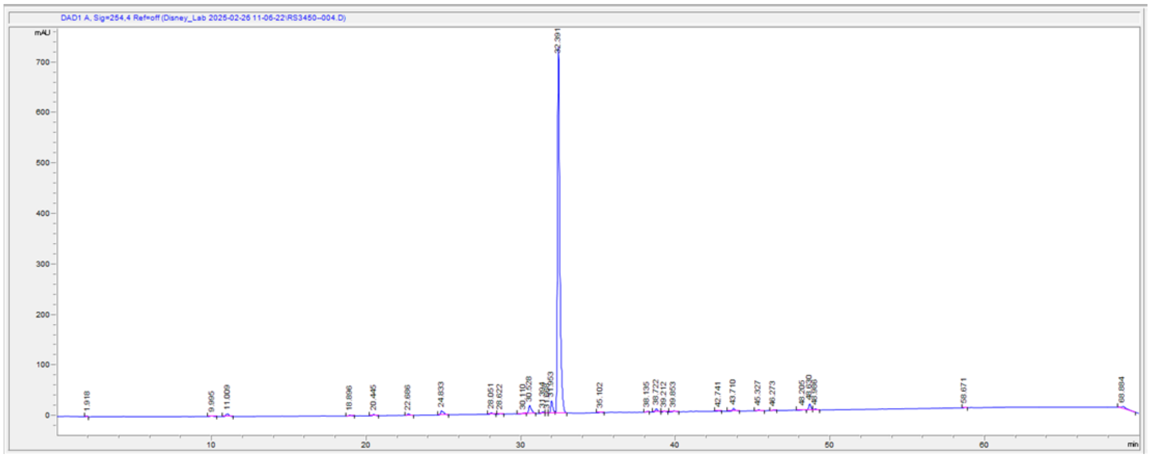

Figure S38. Analytical HPLC trace of **A4**: 0-100%/60 min MeOH/H2O (0.1% (v/v) TFA), where absorbance was measured at 254 nm.

Compound A5:

^{1}H NMR (400 MHz, $CDCl_3$): δ= 7.45 (s, 1H), 7.39-7.20 (m, 6H), 7.13-7.05 (m, 1H), 3.91-3.78 (m, 4H), 3.73 (q, *J* = 6.8 Hz, 2H), 2.95 (t, *J* = 7.2 Hz, 2H), 2.29 (d, *J* = 1.0 Hz, 3H), 2.10-1.98 (m, 4H); HR-MS (MALDI): Calcd. for $C_{20}H_{24}N_5O^+$ $[M+H]^+$, 350.1975; found, 350.1930

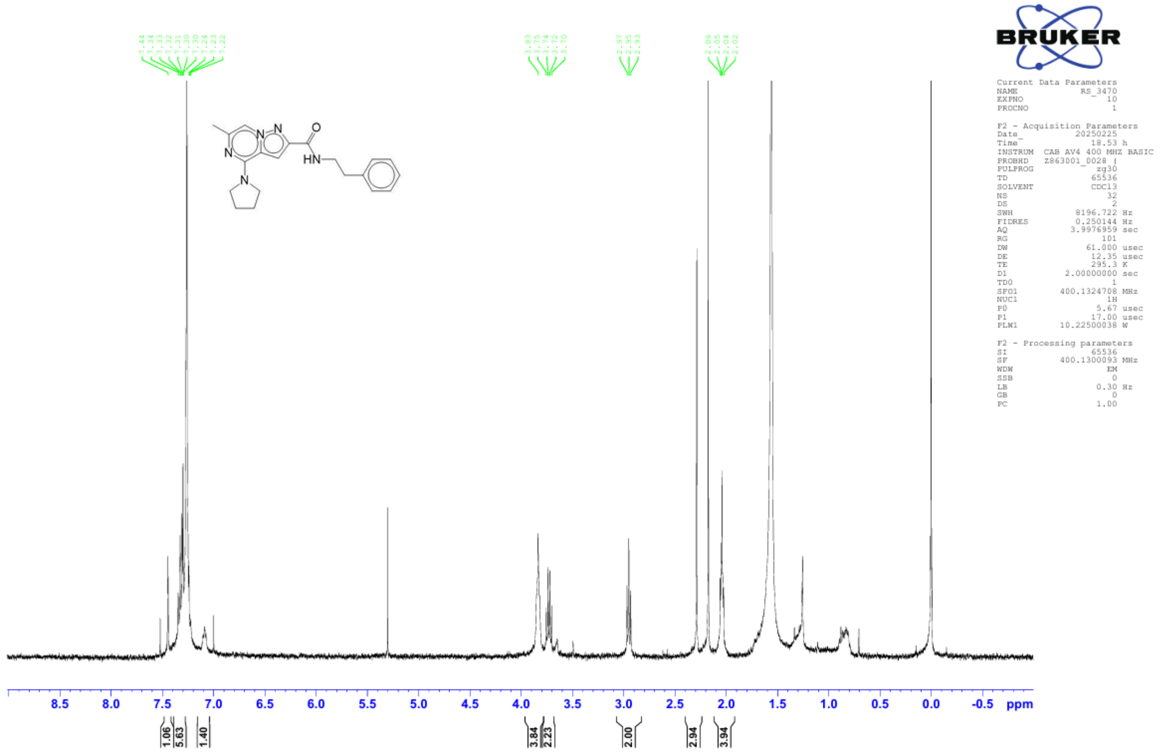

Figure S39. ^{1}H NMR spectrum of **A5**.

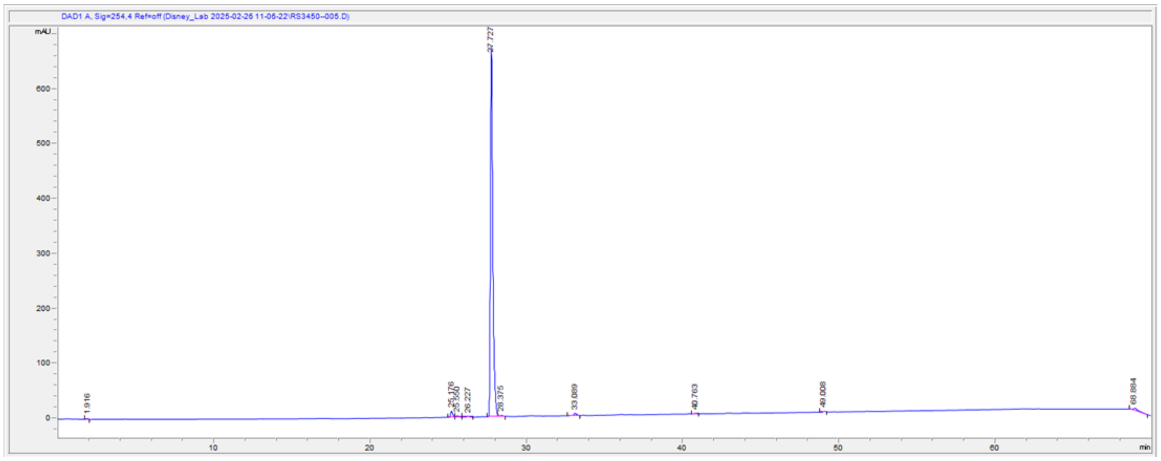

Figure S40. Analytical HPLC trace of **A5**: 0-100%/60 min MeOH/H2O (0.1% (v/v) TFA), where absorbance was measured at 254 nm.

Compound B1:
^{1}H NMR (400 MHz, $CDCl_3$): δ= 7.60-7.57 (m, 1H), 7.32-7.24 (m, 3H), 7.18-7.14 (m, 3H), 4.63 (d, *J* = 5.9, 2H), 4.15-4.10 (m, 4H), 2.80-2.75 (m, 4H), 2.34 (s, 3H), 2.31 (d, *J* = 1.0 Hz, 3H); HR-MS (MALDI): Calcd. for $C_{20}H_{24}N_5OS^+$ $[M+H]^+$, 382.1696; found, 382.1608

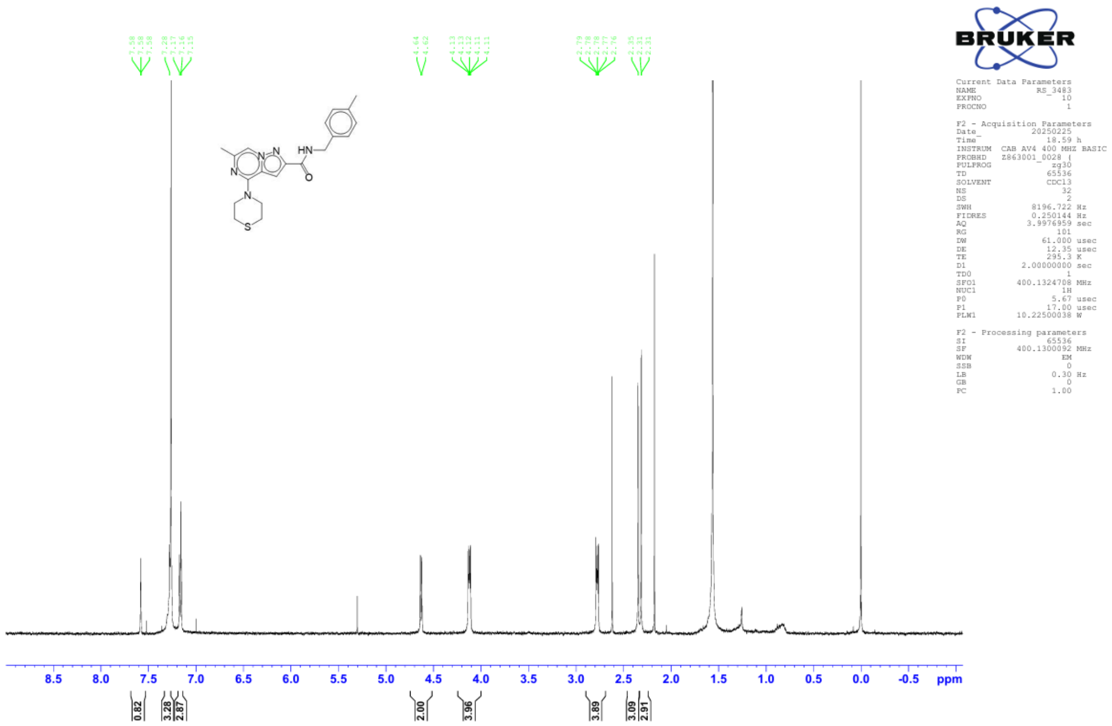

Figure S41. ^{1}H NMR spectrum of **B1**.

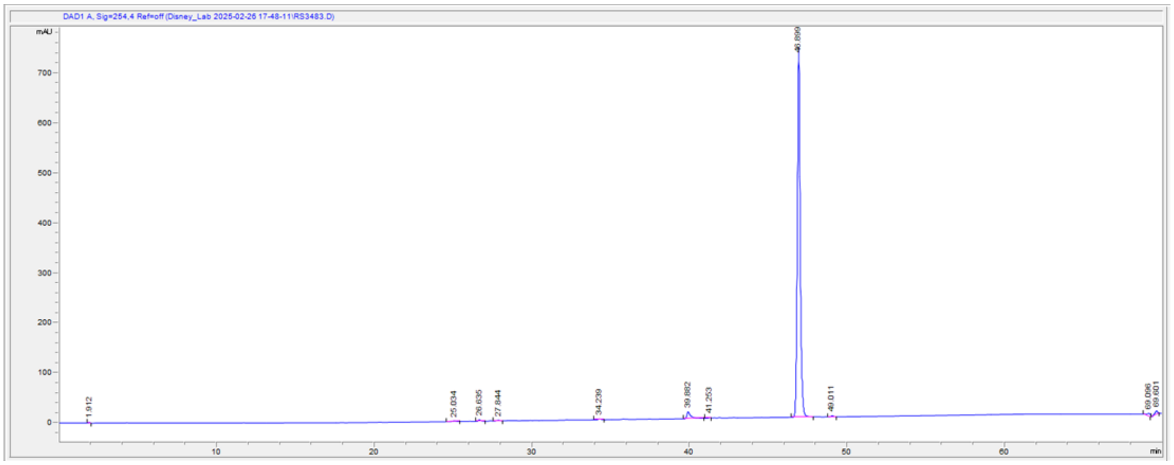

Figure S42. Analytical HPLC trace of **B1**: 0-100%/60 min MeOH/H2O (0.1% (v/v) TFA), where absorbance was measured at 254 nm.

Compound B2:

^{1}H NMR (400 MHz, $CDCl_3$): δ= 7.61 (s, 1H), 7.50-7.43 (m, 1H), 7.36 (dd, *J* = 7.4, 1.5 Hz, 1H), 7.29 (dd, *J* = 8.0, 2.0 Hz, 1H), 7.13 (d, *J* = 0.88 Hz, 1H), 6.96-6.88 (m, 2H), 4.67 (d, *J* = 6.0 Hz, 2H), 4.14-4.08 (m, 4H), 3.90 (s, 3H), 2.79-2.74 (m, 4H), 2.31 (d, *J* = 1.0 Hz, 3H); HR-MS (MALDI): Calcd. for C20H24N5O2S^{+} [M+H]$^{+}$, 398.1645; found, 398.1585

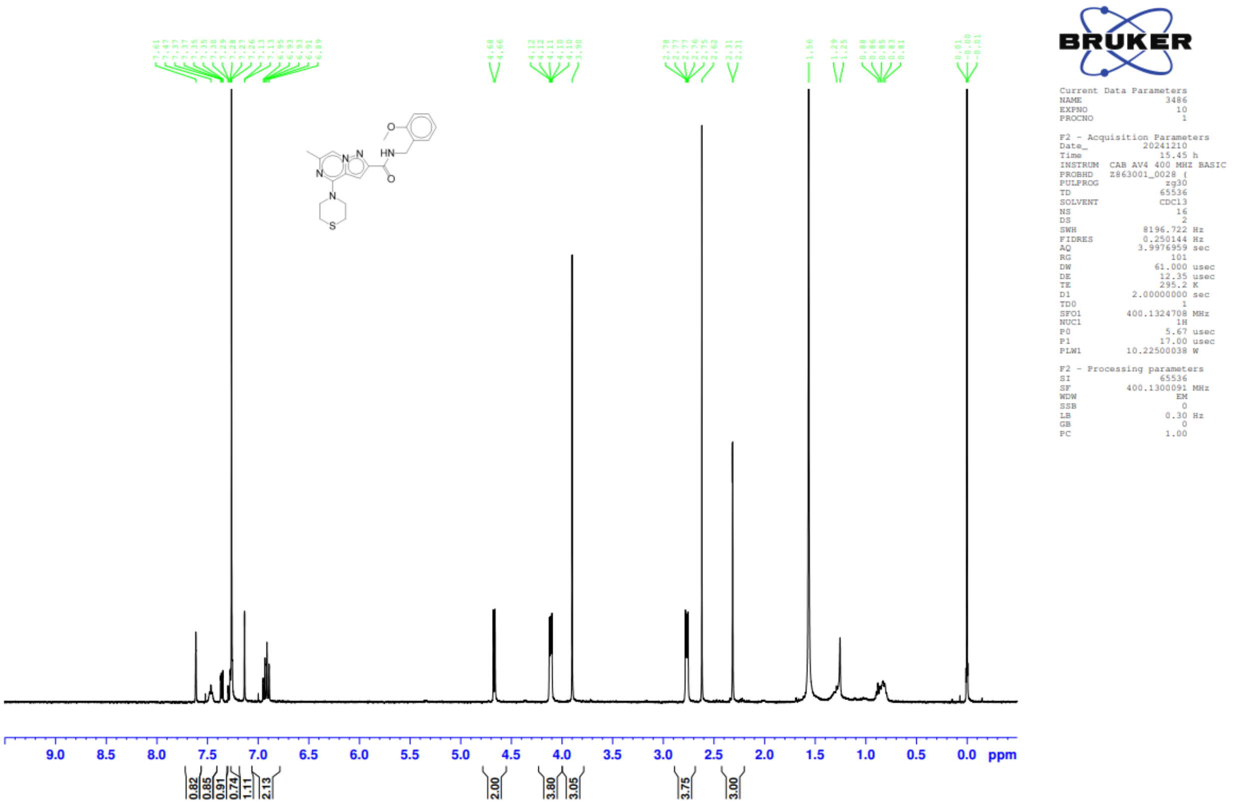

Figure S43. ^{1}H NMR spectrum of **B2**.

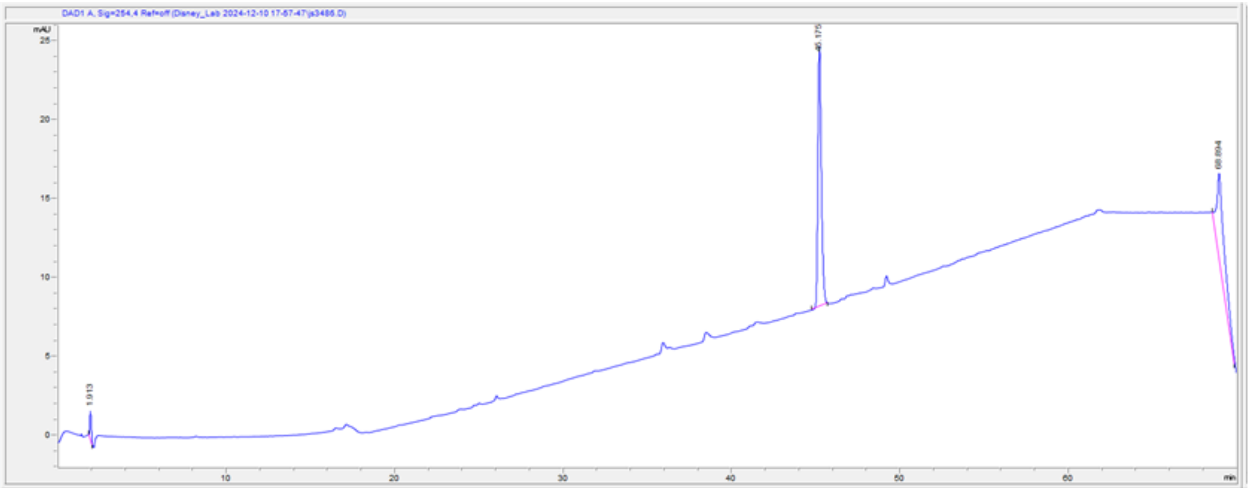

Figure S44. Analytical HPLC trace of **B2**: 0-100%/60 min MeOH/H2O (0.1% (v/v) TFA), where absorbance was measured at 254 nm.

Compound B3:
^{1}H NMR (400 MHz, $CDCl_3$): δ= 7.59-7.57 (m, 1H), 7.30 (dd, *J* = 6.6, 2.1 Hz, 2H), 7.33-7.23 (m, 1H), 7.16 (d, *J* = 1.3 Hz, 1H), 6.89 (dd, *J* = 6.6, 2.1 Hz, 2H), 4.60 (d, *J* = 5.9 Hz, 2H), 4.15-4.09 (m, 4H), 3.80 (s, 3H), 2.75-2.80 (m, 4H), 2.31 (d, *J* = 1.1 Hz, 3H); HR-MS (MALDI): Calcd. for $C_{20}H_{24}N_5O_2S^+$ [M+H]$^+$, 398.1645; found, 398.1597

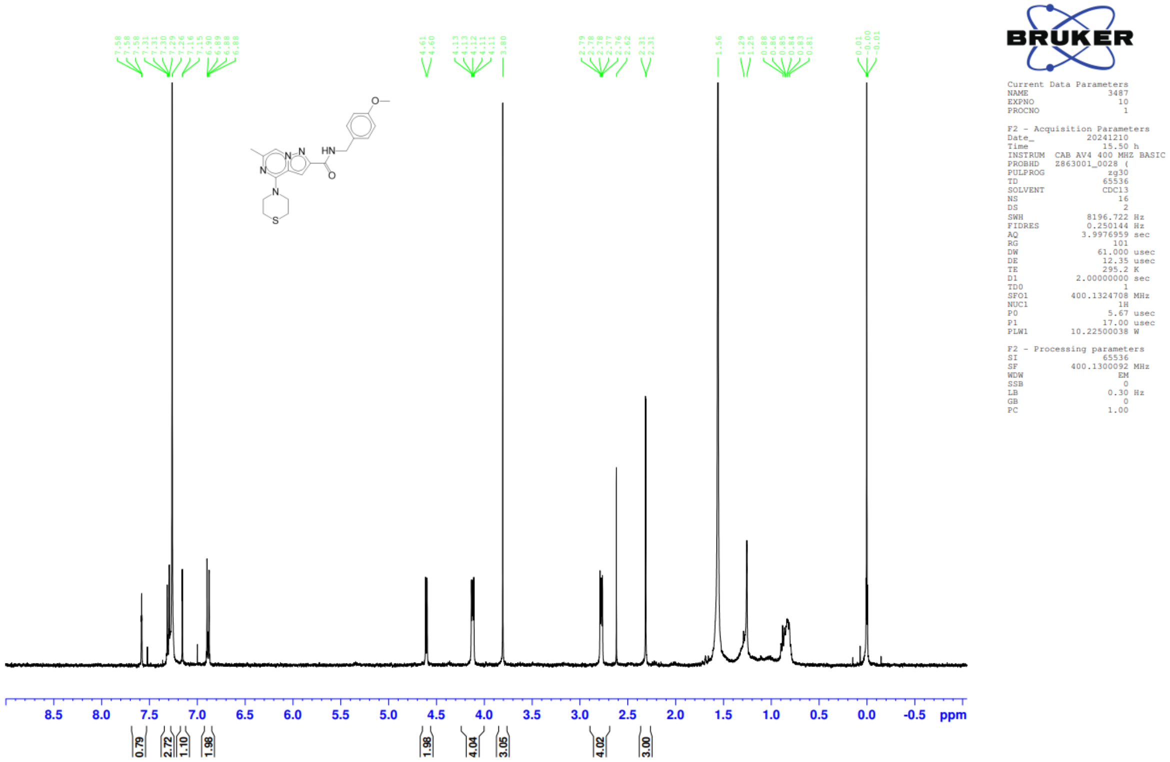

Figure S45. ^{1}H NMR spectrum of **B3**.

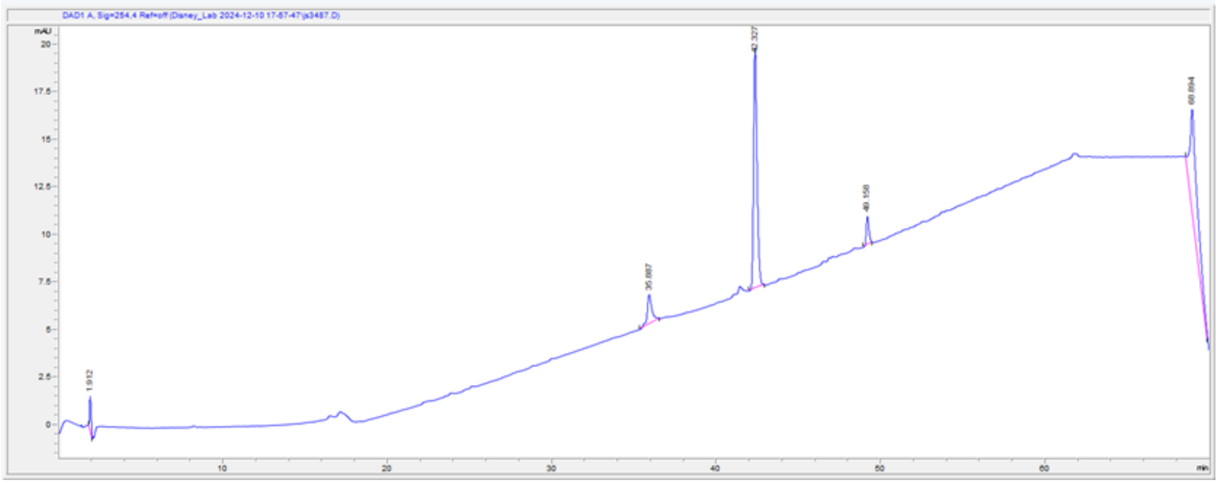

Figure S46. Analytical HPLC trace of **B3**: 0-100%/60 min MeOH/H2O (0.1% (v/v) TFA), where absorbance was measured at 254 nm.

Compound B4:
^{1}H NMR (400 MHz, $CDCl_3$): δ= 7.61-7.58 (m, 1H), 7.20-7.05 (m, 6H), 4.17-4.08 (m, 4H), 3.71 (q, *J* = 6.8 Hz, 2H), 2.91 (t, *J* = 7.1 Hz, 2H), 2.81-2.73 (m, 4H), 2.33 (s, 3H), 2.32 (d, *J* = 0.9 Hz, 3H); HR-MS (MALDI): Calcd. for $C_{21}H_{26}N_5OS^+$ $[M+H]^+$, 396.1853; found, 396.1878

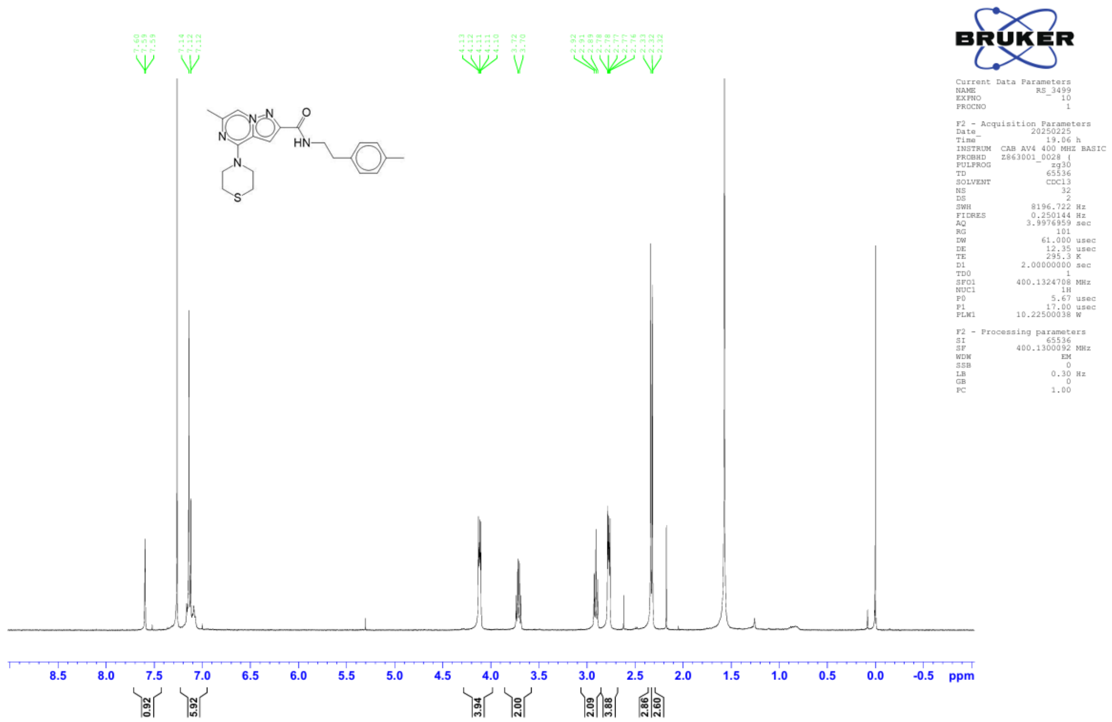

Figure S47. ^{1}H NMR spectrum of **B4**.

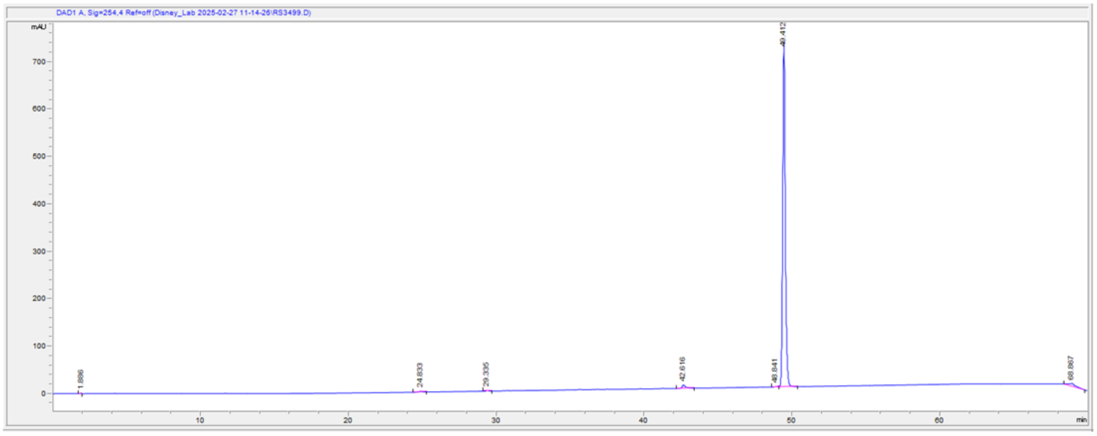

Figure S48. Analytical HPLC trace of **B4**: 0-100%/60 min MeOH/H_2O (0.1% (v/v) TFA) where absorbance was measured at 254 nm.

REFERENCES

(1) Reddy, K.; Jenquin, J. R.; McConnell, O. L.; Cleary, J. D.; Richardson, J. I.; Pinto, B. S.; Haerle, M. C.; Delgado, E.; Planco, L.; Nakamori, M.; et al. A CTG repeat-selective chemical screen identifies microtubule inhibitors as selective modulators of toxic CUG RNA levels. *Proc Natl Acad Sci U S A* **2019**, *116* (42), 20991-21000.
(2) Childs-Disney, J. L.; Stepniak-Konieczna, E.; Tran, T.; Yildirim, I.; Park, H.; Chen, C. Z.; Hoskins, J.; Southall, N.; Marugan, J. J.; Patnaik, S.; et al. Induction and reversal of myotonic dystrophy type 1 pre-mRNA splicing defects by small molecules. *Nat Commun* **2013**, *4*, 2044.
(3) Zhang, J. H.; Chung, T. D.; Oldenburg, K. R. A simple statistical parameter for use in evaluation and validation of high throughput screening assays. *J Biomol Screen* **1999**, *4* (2), 67-73.
